# Supplementary material for: Large gains in schooling and income are possible from minimizing adverse birth outcomes in 121 low- and middle-income countries: A modelling study
Source: PLOS Glob Public Health. 2022 Jun 8;2(6):e0000218. doi: 10.1371/journal.pgph.0000218 (PMC10021521; doi:10.1371/journal.pgph.0000218)
Supplement: S1 Table — (DOCX) [file pgph.0000218.s001.docx]

**Supplement 1: Table: Data sources**

| Data input | Source |
| --- | --- |
| Birth outcome prevalence | |
| Low birthweight (LBW) | Lee et al. 2013 (supplementary material).  Impute the GBD subregion mean for missing data. |
| Preterm birth (PTB) | Chawanpaiboon 2019 and Blencowe 2019  Impute the GBD subregion mean for missing data. |
| Small-for-gestational age (SGA) | Lee et al. 2013 (supplementary material). |
| Data for estimating human capital outcomes | |
| Birth cohorts and survival probabilities to age 5 and 25 | United Nations Population Division World Population Prospects 2019. |
| Annual wages | [World Indicators Database](https://data.worldbank.org/indicator/NY.GDP.MKTP.KD) (2/3 of GDP in 2010 constant US$) |
| Survival probabilities | Institute for Health Metrics Life tables (and United Nations Population Division World Population Prospects 2019 |
