# Supplementary material for: Large gains in schooling and income are possible from minimizing adverse birth outcomes in 121 low- and middle-income countries: A modelling study
Source: PLOS Glob Public Health. 2022 Jun 8;2(6):e0000218. doi: 10.1371/journal.pgph.0000218 (PMC10021521; doi:10.1371/journal.pgph.0000218)
Supplement: S1 Text — (DOCX) [file pgph.0000218.s002.docx]

**Supplement 1 text: Working example**

Working example:

Below we provide an illustrative example of the impact of shifting the prevalence of low birthweight in Ethiopia from 20.3% (estimated prevalence in 2015) to the theoretical minimum of 3.2%. Numbers will vary slightly from those displayed in the supplemental country profiles as the latter are simulated with propagated uncertainty.

Step 1, calculate the reduction in LBW prevalence:

Absolute fractional reduction in prevalence of low birthweight

= (Current prevalence – theoretical minimum prevalence) /100

= (20.3-3.2)/100 = 0.171 fractional reduction in LBW

Step 2, estimate the impact of birth outcome on expected additional attained years of schooling:

Absolute reduction in LBW prevalence * difference in expected years of schooling between LBW and non-LBW children

= 0.171 * 0.2894 = 0.0494874 additional years of schooling per child

Step 3, estimate the impact on total years of schooling attained:

Additional years of schooling per child * size of birth cohort * survival probability until age 25

= 0.0494874 * 17,572,000 * 0.89651 ≈ 779,598 additional school years per Ethiopian birth cohort experiencing a theoretical minimum prevalence of LBW.

Step 4, estimate the impact on lifetime wages per birth cohort (20 to 59yrs) (in USD millions):

Total school years gained per birth cohort * country-specific net present value of lifetime wages for a 40 year working period * country-specific returns in % income per additional year of schooling = 779,598 * ($321.76 annual wage * 27.06 income multiplier for net present value) * 0.1247 = $US 846,441,631
