# Supplementary material for: Large gains in schooling and income are possible from minimizing adverse birth outcomes in 121 low- and middle-income countries: A modelling study
Source: PLOS Glob Public Health. 2022 Jun 8;2(6):e0000218. doi: 10.1371/journal.pgph.0000218 (PMC10021521; doi:10.1371/journal.pgph.0000218)
Supplement: S1 Data — (DOCX) [file pgph.0000218.s003.docx]

**S1 Data: Country-specific estimates**

**Afghanistan**

**Super region:** North Africa and Middle East**; Sub region:** North Africa and Middle East

**Number of live births^3^:** 6,024,000

**Probability of survival to age 25^2^**: 0.89

**GDP per capita 2010 US dollars (estimated annual wage)^3^:**  574

**GDP per capita 2011 International dollars (estimated annual wage)^3^:**  1,767

| **Birth outcome** | **Current**  **prevalence (%)** | **TMRED^1^ (%)** | **Total school years gained per birth cohort**  **(in 1000s)** | **Increase in lifetime earnings in USD per child (20 to 59yrs)** | **Benefits by cohorts (20 to 59yrs)** **Lifetime wages**  **(in USD millions)** |
| --- | --- | --- | --- | --- | --- |
| **LBW** | 20.0 (16.0,24.0) | 3.2 | 260.3 (72.4,456.4) | 34.5 (9.7,61.2) | 208.1 (58.2,368.4) |
| **PTB** | 10.4 (8.7,11.9) | 5.5 | 80.1 (11.3,172.9) | 10.6 (1.6,23.1) | 63.8 (9.5,139.4) |
| **SGA** | 40.0 (34.6,45.6) | 10 | 652.7 (317.1,1061.7) | 86.8 (42.1,145.5) | 522.6 (253.8,876.4) |

Impact of sub-optimal prevalences in low birthweight (LBW), preterm birth (PTB), or small for gestational age (SGA) on human capital and labor market outcomes in Afghanistan.

^1^ TMRED = Theoretical Minimum Risk Exposure Distribution (TMRED)

^2^ Source: United National Population Division World Population Prospects 2019.

^3^ Country specific annual wage data from World Indicators Database. Average yearly wage was estimated to be 2/3 of the gross domestic product in 2010 constant US dollars and 2011 International dollars, adjusted for purchasing power parity.

**Algeria**

**Super region:** North Africa and Middle East**; Sub region:** North Africa and Middle East

**Number of live births^3^:** 5,159,000

**Probability of survival to age 25^2^**: 0.96

**GDP per capita 2010 US dollars (estimated annual wage)^3^:**  4,777

**GDP per capita 2011 International dollars (estimated annual wage)^3^:**  13,774

| **Birth outcome** | **Current**  **prevalence (%)** | **TMRED^1^ (%)** | **Total school years gained per birth cohort**  **(in 1000s)** | **Increase in lifetime earnings in USD per child (20 to 59yrs)** | **Benefits by cohorts (20 to 59yrs)** **Lifetime wages**  **(in USD millions)** |
| --- | --- | --- | --- | --- | --- |
| **LBW** | 7.3 (5.7,9.6) | 3.2 | 56.1 (14.6,115.9) | 63.1 (14.7,145.3) | 325.6 (75.6,749.8) |
| **PTB** | 13.4 (6.3,30.9) | 5.5 | 103.8 (0.0,397.8) | 117.3 (0.0,479.3) | 605.1 (0.0,2472.8) |
| **SGA** | 10.6 (7.7,14.2) | 10 | 12.8 (0.0,90.2) | 13.4 (0.0,105.7) | 69.0 (0.0,545.2) |

Impact of sub-optimal prevalences in low birthweight (LBW), preterm birth (PTB), or small for gestational age (SGA) on human capital and labor market outcomes in Algeria.

^1^ TMRED = Theoretical Minimum Risk Exposure Distribution (TMRED)

^2^ Source: United National Population Division World Population Prospects 2019.

^3^ Country specific annual wage data from World Indicators Database. Average yearly wage was estimated to be 2/3 of the gross domestic product in 2010 constant US dollars and 2011 International dollars, adjusted for purchasing power parity.

**Angola**

**Super region:** Sub-Saharan Africa**; Sub region:** Central Sub-Saharan Africa

**Number of live births^3^:** 6,215,000

**Probability of survival to age 25^2^**: 0.86

**GDP per capita 2010 US dollars (estimated annual wage)^3^:**  3,748

**GDP per capita 2011 International dollars (estimated annual wage)^3^:**  6,645

| **Birth outcome** | **Current**  **prevalence (%)** | **TMRED^1^ (%)** | **Total school years gained per birth cohort**  **(in 1000s)** | **Increase in lifetime earnings in USD per child (20 to 59yrs)** | **Benefits by cohorts (20 to 59yrs)** **Lifetime wages**  **(in USD millions)** |
| --- | --- | --- | --- | --- | --- |
| **LBW** | 15.3 (11.8,21.4) | 3.2 | 183.1 (48.7,349.1) | 196.5 (52.4,397.2) | 1221.2 (325.4,2468.4) |
| **PTB** | 12.0 (8.6,16.7) | 5.5 | 100.8 (12.5,254.9) | 106.7 (12.4,284.1) | 663.1 (77.0,1765.5) |
| **SGA** | 25.6 (21.9,29.5) | 10 | 337.5 (151.9,564.3) | 361.2 (148.2,653.1) | 2244.6 (920.8,4059.3) |

Impact of sub-optimal prevalences in low birthweight (LBW), preterm birth (PTB), or small for gestational age (SGA) on human capital and labor market outcomes in Angola.

^1^ TMRED = Theoretical Minimum Risk Exposure Distribution (TMRED)

^2^ Source: United National Population Division World Population Prospects 2019.

^3^ Country specific annual wage data from World Indicators Database. Average yearly wage was estimated to be 2/3 of the gross domestic product in 2010 constant US dollars and 2011 International dollars, adjusted for purchasing power parity.

**Argentina**

**Super region:** Latin America and Caribbean**; Sub region:** Southern Latin America

**Number of live births^3^:** 3,776,000

**Probability of survival to age 25^2^**: 0.97

**GDP per capita 2010 US dollars (estimated annual wage)^3^:**  10,568

**GDP per capita 2011 International dollars (estimated annual wage)^3^:**  19,244

| **Birth outcome** | **Current**  **prevalence (%)** | **TMRED^1^ (%)** | **Total school years gained per birth cohort**  **(in 1000s)** | **Increase in lifetime earnings in USD per child (20 to 59yrs)** | **Benefits by cohorts (20 to 59yrs)** **Lifetime wages**  **(in USD millions)** |
| --- | --- | --- | --- | --- | --- |
| **LBW** | 7.3 (7.1,7.6) | 3.2 | 43.9 (12.8,72.5) | 267.9 (75.9,461.6) | 1011.4 (286.7,1742.8) |
| **PTB** | 8.4 (6.7,10.2) | 5.5 | 32.2 (4.3,72.5) | 198.4 (27.2,451.3) | 749.3 (102.5,1703.9) |
| **SGA** | 11.3 (8.2,15.0) | 10 | 16.9 (0.0,77.8) | 104.2 (0.0,481.5) | 393.5 (0.0,1818.2) |

Impact of sub-optimal prevalences in low birthweight (LBW), preterm birth (PTB), or small for gestational age (SGA) on human capital and labor market outcomes in Argentina.

^1^ TMRED = Theoretical Minimum Risk Exposure Distribution (TMRED)

^2^ Source: United National Population Division World Population Prospects 2019.

^3^ Country specific annual wage data from World Indicators Database. Average yearly wage was estimated to be 2/3 of the gross domestic product in 2010 constant US dollars and 2011 International dollars, adjusted for purchasing power parity.

**Armenia**

**Super region:** Central Europe, Eastern Europe, Central Asia**; Sub region:** Central Asia

**Number of live births^3^:** 209,000

**Probability of survival to age 25^2^**: 0.98

**GDP per capita 2010 US dollars (estimated annual wage)^3^:**  3,924

**GDP per capita 2011 International dollars (estimated annual wage)^3^:**  8,172

| **Birth outcome** | **Current**  **prevalence (%)** | **TMRED^1^ (%)** | **Total school years gained per birth cohort**  **(in 1000s)** | **Increase in lifetime earnings in USD per child (20 to 59yrs)** | **Benefits by cohorts (20 to 59yrs)** **Lifetime wages**  **(in USD millions)** |
| --- | --- | --- | --- | --- | --- |
| **LBW** | 9.0 (8.2,9.7) | 3.2 | 3.4 (1.0,5.8) | 56.5 (14.4,116.3) | 11.8 (3.0,24.3) |
| **PTB** | 10.4 (8.7,11.9) | 5.5 | 3.0 (0.4,6.2) | 51.0 (7.0,131.3) | 10.7 (1.5,27.4) |
| **SGA** | 16.4 (11.5,22.5) | 10 | 5.0 (0.5,11.1) | 84.5 (6.3,217.4) | 17.7 (1.3,45.4) |

Impact of sub-optimal prevalences in low birthweight (LBW), preterm birth (PTB), or small for gestational age (SGA) on human capital and labor market outcomes in Armenia.

^1^ TMRED = Theoretical Minimum Risk Exposure Distribution (TMRED)

^2^ Source: United National Population Division World Population Prospects 2019.

^3^ Country specific annual wage data from World Indicators Database. Average yearly wage was estimated to be 2/3 of the gross domestic product in 2010 constant US dollars and 2011 International dollars, adjusted for purchasing power parity.

**Azerbaijan**

**Super region:** Central Europe, Eastern Europe, Central Asia**; Sub region:** Central Asia

**Number of live births^3^:** 844,000

**Probability of survival to age 25^2^**: 0.96

**GDP per capita 2010 US dollars (estimated annual wage)^3^:**  6,064

**GDP per capita 2011 International dollars (estimated annual wage)^3^:**  16,829

| **Birth outcome** | **Current**  **prevalence (%)** | **TMRED^1^ (%)** | **Total school years gained per birth cohort**  **(in 1000s)** | **Increase in lifetime earnings in USD per child (20 to 59yrs)** | **Benefits by cohorts (20 to 59yrs)** **Lifetime wages**  **(in USD millions)** |
| --- | --- | --- | --- | --- | --- |
| **LBW** | 7.3 (6.4,8.2) | 3.2 | 9.4 (2.8,16.9) | 47.5 (13.5,89.3) | 40.1 (11.4,75.4) |
| **PTB** | 10.4 (8.7,11.9) | 5.5 | 12.3 (1.9,25.1) | 60.8 (9.3,128.2) | 51.3 (7.8,108.2) |
| **SGA** | 16.2 (11.4,22.5) | 10 | 19.2 (1.5,46.2) | 95.7 (6.8,233.1) | 80.8 (5.7,196.7) |

Impact of sub-optimal prevalences in low birthweight (LBW), preterm birth (PTB), or small for gestational age (SGA) on human capital and labor market outcomes in Azerbaijan.

^1^ TMRED = Theoretical Minimum Risk Exposure Distribution (TMRED)

^2^ Source: United National Population Division World Population Prospects 2019.

^3^ Country specific annual wage data from World Indicators Database. Average yearly wage was estimated to be 2/3 of the gross domestic product in 2010 constant US dollars and 2011 International dollars, adjusted for purchasing power parity.

**Bangladesh**

**Super region:** South Asia**; Sub region:** South Asia

**Number of live births^3^:** 14,732,000

**Probability of survival to age 25^2^**: 0.95

**GDP per capita 2010 US dollars (estimated annual wage)^3^:**  1,002

**GDP per capita 2011 International dollars (estimated annual wage)^3^:**  3,232

| **Birth outcome** | **Current**  **prevalence (%)** | **TMRED^1^ (%)** | **Total school years gained per birth cohort**  **(in 1000s)** | **Increase in lifetime earnings in USD per child (20 to 59yrs)** | **Benefits by cohorts (20 to 59yrs)** **Lifetime wages**  **(in USD millions)** |
| --- | --- | --- | --- | --- | --- |
| **LBW** | 27.8 (19.6,38.5) | 3.2 | 948.2 (270.9,1837.5) | 82.9 (23.5,161.0) | 1221.3 (346.1,2371.3) |
| **PTB** | 19.1 (13.2,26.2) | 5.5 | 566.6 (70.3,1255.3) | 49.3 (6.3,109.2) | 726.3 (92.3,1609.2) |
| **SGA** | 39.6 (35.7,45.6) | 10 | 1671.3 (815.9,2670.3) | 146.0 (70.2,236.5) | 2150.3 (1034.6,3483.6) |

Impact of sub-optimal prevalences in low birthweight (LBW), preterm birth (PTB), or small for gestational age (SGA) on human capital and labor market outcomes in Bangladesh.

^1^ TMRED = Theoretical Minimum Risk Exposure Distribution (TMRED)

^2^ Source: United National Population Division World Population Prospects 2019.

^3^ Country specific annual wage data from World Indicators Database. Average yearly wage was estimated to be 2/3 of the gross domestic product in 2010 constant US dollars and 2011 International dollars, adjusted for purchasing power parity.

**Belize**

**Super region:** Latin America and Caribbean**; Sub region:** Caribbean

**Number of live births^3^:** 40,000

**Probability of survival to age 25^2^**: 0.96

**GDP per capita 2010 US dollars (estimated annual wage)^3^:**  4,402

**GDP per capita 2011 International dollars (estimated annual wage)^3^:**  7,977

| **Birth outcome** | **Current**  **prevalence (%)** | **TMRED^1^ (%)** | **Total school years gained per birth cohort**  **(in 1000s)** | **Increase in lifetime earnings in USD per child (20 to 59yrs)** | **Benefits by cohorts (20 to 59yrs)** **Lifetime wages**  **(in USD millions)** |
| --- | --- | --- | --- | --- | --- |
| **LBW** | 8.6 (6.8,11.1) | 3.2 | 0.6 (0.2,1.1) | 113.8 (29.1,213.1) | 4.6 (1.2,8.5) |
| **PTB** | 9.8 (8.6,11.3) | 5.5 | 0.5 (0.1,1.0) | 100.2 (15.5,207.8) | 4.0 (0.6,8.3) |
| **SGA** | 20.0 (15.4,25.6) | 10 | 1.5 (0.6,2.8) | 297.8 (112.0,556.5) | 11.9 (4.5,22.3) |

Impact of sub-optimal prevalences in low birthweight (LBW), preterm birth (PTB), or small for gestational age (SGA) on human capital and labor market outcomes in Belize.

^1^ TMRED = Theoretical Minimum Risk Exposure Distribution (TMRED)

^2^ Source: United National Population Division World Population Prospects 2019.

^3^ Country specific annual wage data from World Indicators Database. Average yearly wage was estimated to be 2/3 of the gross domestic product in 2010 constant US dollars and 2011 International dollars, adjusted for purchasing power parity.

**Benin**

**Super region:** Sub-Saharan Africa**; Sub region:** Western Sub-Saharan Africa

**Number of live births^3^:** 2,066,000

**Probability of survival to age 25^2^**: 0.84

**GDP per capita 2010 US dollars (estimated annual wage)^3^:**  828

**GDP per capita 2011 International dollars (estimated annual wage)^3^:**  1,987

| **Birth outcome** | **Current**  **prevalence (%)** | **TMRED^1^ (%)** | **Total school years gained per birth cohort**  **(in 1000s)** | **Increase in lifetime earnings in USD per child (20 to 59yrs)** | **Benefits by cohorts (20 to 59yrs)** **Lifetime wages**  **(in USD millions)** |
| --- | --- | --- | --- | --- | --- |
| **LBW** | 16.9 (13.3,21.3) | 3.2 | 67.7 (18.4,123.7) | 30.2 (8.7,57.1) | 62.4 (17.9,118.0) |
| **PTB** | 9.3 (6.0,13.6) | 5.5 | 19.6 (0.0,52.9) | 8.6 (0.0,26.1) | 17.7 (0.0,54.0) |
| **SGA** | 25.8 (21.5,29.4) | 10 | 111.5 (50.6,181.1) | 49.5 (21.7,88.6) | 102.4 (44.8,183.0) |

Impact of sub-optimal prevalences in low birthweight (LBW), preterm birth (PTB), or small for gestational age (SGA) on human capital and labor market outcomes in Benin.

^1^ TMRED = Theoretical Minimum Risk Exposure Distribution (TMRED)

^2^ Source: United National Population Division World Population Prospects 2019.

^3^ Country specific annual wage data from World Indicators Database. Average yearly wage was estimated to be 2/3 of the gross domestic product in 2010 constant US dollars and 2011 International dollars, adjusted for purchasing power parity.

**Bhutan**

**Super region:** South Asia**; Sub region:** South Asia

**Number of live births^3^:** 66,000

**Probability of survival to age 25^2^**: 0.94

**GDP per capita 2010 US dollars (estimated annual wage)^3^:**  2,844

**GDP per capita 2011 International dollars (estimated annual wage)^3^:**  8,380

| **Birth outcome** | **Current**  **prevalence (%)** | **TMRED^1^ (%)** | **Total school years gained per birth cohort**  **(in 1000s)** | **Increase in lifetime earnings in USD per child (20 to 59yrs)** | **Benefits by cohorts (20 to 59yrs)** **Lifetime wages**  **(in USD millions)** |
| --- | --- | --- | --- | --- | --- |
| **LBW** | 11.7 (8.2,18.5) | 3.2 | 1.5 (0.3,3.2) | 89.3 (20.2,191.1) | 5.9 (1.3,12.6) |
| **PTB** | 10.4 (8.7,11.9) | 5.5 | 0.9 (0.2,1.9) | 56.6 (8.6,110.8) | 3.7 (0.6,7.3) |
| **SGA** | 21.7 (16.5,28.8) | 10 | 2.8 (1.0,5.6) | 171.3 (63.8,331.6) | 11.3 (4.2,21.9) |

Impact of sub-optimal prevalences in low birthweight (LBW), preterm birth (PTB), or small for gestational age (SGA) on human capital and labor market outcomes in Bhutan.

^1^ TMRED = Theoretical Minimum Risk Exposure Distribution (TMRED)

^2^ Source: United National Population Division World Population Prospects 2019.

^3^ Country specific annual wage data from World Indicators Database. Average yearly wage was estimated to be 2/3 of the gross domestic product in 2010 constant US dollars and 2011 International dollars, adjusted for purchasing power parity.

**Bolivia**

**Super region:** Latin America and Caribbean**; Sub region:** Andean Latin America

**Number of live births^3^:** 1,233,000

**Probability of survival to age 25^2^**: 0.91

**GDP per capita 2010 US dollars (estimated annual wage)^3^:**  2,361

**GDP per capita 2011 International dollars (estimated annual wage)^3^:**  6,444

| **Birth outcome** | **Current**  **prevalence (%)** | **TMRED^1^ (%)** | **Total school years gained per birth cohort**  **(in 1000s)** | **Increase in lifetime earnings in USD per child (20 to 59yrs)** | **Benefits by cohorts (20 to 59yrs)** **Lifetime wages**  **(in USD millions)** |
| --- | --- | --- | --- | --- | --- |
| **LBW** | 7.2 (5.7,9.3) | 3.2 | 12.4 (3.1,25.8) | 28.8 (7.6,61.2) | 35.5 (9.4,75.4) |
| **PTB** | 9.8 (8.6,11.3) | 5.5 | 14.8 (2.5,30.5) | 34.6 (4.8,73.6) | 42.7 (6.0,90.8) |
| **SGA** | 9.8 (6.1,12.6) | 10 | 0.0 (0.0,13.8) | 0.0 (0.0,34.4) | 0.0 (0.0,42.4) |

Impact of sub-optimal prevalences in low birthweight (LBW), preterm birth (PTB), or small for gestational age (SGA) on human capital and labor market outcomes in Bolivia.

^1^ TMRED = Theoretical Minimum Risk Exposure Distribution (TMRED)

^2^ Source: United National Population Division World Population Prospects 2019.

^3^ Country specific annual wage data from World Indicators Database. Average yearly wage was estimated to be 2/3 of the gross domestic product in 2010 constant US dollars and 2011 International dollars, adjusted for purchasing power parity.

**Botswana**

**Super region:** Sub-Saharan Africa**; Sub region:** Southern Sub-Saharan Africa

**Number of live births^3^:** 281,000

**Probability of survival to age 25^2^**: 0.94

**GDP per capita 2010 US dollars (estimated annual wage)^3^:**  7,614

**GDP per capita 2011 International dollars (estimated annual wage)^3^:**  15,660

| **Birth outcome** | **Current**  **prevalence (%)** | **TMRED^1^ (%)** | **Total school years gained per birth cohort**  **(in 1000s)** | **Increase in lifetime earnings in USD per child (20 to 59yrs)** | **Benefits by cohorts (20 to 59yrs)** **Lifetime wages**  **(in USD millions)** |
| --- | --- | --- | --- | --- | --- |
| **LBW** | 15.6 (12.3,20.6) | 3.2 | 9.5 (3.8,10.6) | 733.0 (294.2,841.3) | 206.0 (82.7,236.4) |
| **PTB** | 12.0 (8.6,16.7) | 5.5 | 5.0 (0.7,12.1) | 387.5 (51.1,921.5) | 108.9 (14.4,258.9) |
| **SGA** | 20.4 (15.3,25.5) | 10 | 10.8 (4.2,19.8) | 847.0 (329.6,1533.6) | 238.0 (92.6,430.9) |

Impact of sub-optimal prevalences in low birthweight (LBW), preterm birth (PTB), or small for gestational age (SGA) on human capital and labor market outcomes in Botswana.

^1^ TMRED = Theoretical Minimum Risk Exposure Distribution (TMRED)

^2^ Source: United National Population Division World Population Prospects 2019.

^3^ Country specific annual wage data from World Indicators Database. Average yearly wage was estimated to be 2/3 of the gross domestic product in 2010 constant US dollars and 2011 International dollars, adjusted for purchasing power parity.

**Brazil**

**Super region:** Latin America and Caribbean**; Sub region:** Tropical Latin America

**Number of live births^3^:** 14,672,000

**Probability of survival to age 25^2^**: 0.96

**GDP per capita 2010 US dollars (estimated annual wage)^3^:**  11,431

**GDP per capita 2011 International dollars (estimated annual wage)^3^:**  14,807

| **Birth outcome** | **Current**  **prevalence (%)** | **TMRED^1^ (%)** | **Total school years gained per birth cohort**  **(in 1000s)** | **Increase in lifetime earnings in USD per child (20 to 59yrs)** | **Benefits by cohorts (20 to 59yrs)** **Lifetime wages**  **(in USD millions)** |
| --- | --- | --- | --- | --- | --- |
| **LBW** | 8.4 (8.3,8.5) | 3.2 | 212.9 (63.6,358.6) | 398.2 (113.3,677.3) | 5841.8 (1662.8,9936.9) |
| **PTB** | 11.2 (9.4,12.9) | 5.5 | 249.7 (41.6,498.1) | 464.1 (74.0,974.0) | 6809.5 (1085.2,14289.9) |
| **SGA** | 13.4 (9.9,17.6) | 10 | 187.4 (0.0,465.8) | 348.6 (0.0,895.8) | 5114.5 (0.0,13143.6) |

Impact of sub-optimal prevalences in low birthweight (LBW), preterm birth (PTB), or small for gestational age (SGA) on human capital and labor market outcomes in Brazil.

^1^ TMRED = Theoretical Minimum Risk Exposure Distribution (TMRED)

^2^ Source: United National Population Division World Population Prospects 2019.

^3^ Country specific annual wage data from World Indicators Database. Average yearly wage was estimated to be 2/3 of the gross domestic product in 2010 constant US dollars and 2011 International dollars, adjusted for purchasing power parity.

**Burkina Faso**

**Super region:** Sub-Saharan Africa**; Sub region:** Western Sub-Saharan Africa

**Number of live births^3^:** 3,725,000

**Probability of survival to age 25^2^**: 0.85

**GDP per capita 2010 US dollars (estimated annual wage)^3^:**  645

**GDP per capita 2011 International dollars (estimated annual wage)^3^:**  1,596

| **Birth outcome** | **Current**  **prevalence (%)** | **TMRED^1^ (%)** | **Total school years gained per birth cohort**  **(in 1000s)** | **Increase in lifetime earnings in USD per child (20 to 59yrs)** | **Benefits by cohorts (20 to 59yrs)** **Lifetime wages**  **(in USD millions)** |
| --- | --- | --- | --- | --- | --- |
| **LBW** | 13.1 (9.7,16.3) | 3.2 | 88.6 (24.0,166.4) | 17.2 (4.9,35.3) | 64.1 (18.2,131.6) |
| **PTB** | 12.0 (8.6,16.7) | 5.5 | 62.0 (7.9,150.6) | 12.2 (1.5,30.3) | 45.3 (5.8,113.0) |
| **SGA** | 20.8 (17.5,24.1) | 10 | 137.8 (60.4,237.4) | 26.8 (11.0,49.5) | 100.0 (40.9,184.5) |

Impact of sub-optimal prevalences in low birthweight (LBW), preterm birth (PTB), or small for gestational age (SGA) on human capital and labor market outcomes in Burkina Faso.

^1^ TMRED = Theoretical Minimum Risk Exposure Distribution (TMRED)

^2^ Source: United National Population Division World Population Prospects 2019.

^3^ Country specific annual wage data from World Indicators Database. Average yearly wage was estimated to be 2/3 of the gross domestic product in 2010 constant US dollars and 2011 International dollars, adjusted for purchasing power parity.

**Burundi**

**Super region:** Sub-Saharan Africa**; Sub region:** Eastern Sub-Saharan Africa

**Number of live births^3^:** 2,164,000

**Probability of survival to age 25^2^**: 0.86

**GDP per capita 2010 US dollars (estimated annual wage)^3^:**  228

**GDP per capita 2011 International dollars (estimated annual wage)^3^:**  716

| **Birth outcome** | **Current**  **prevalence (%)** | **TMRED^1^ (%)** | **Total school years gained per birth cohort**  **(in 1000s)** | **Increase in lifetime earnings in USD per child (20 to 59yrs)** | **Benefits by cohorts (20 to 59yrs)** **Lifetime wages**  **(in USD millions)** |
| --- | --- | --- | --- | --- | --- |
| **LBW** | 15.1 (10.9,19.4) | 3.2 | 62.6 (18.0,119.7) | 13.4 (3.7,26.1) | 29.0 (7.9,56.4) |
| **PTB** | 12.0 (8.6,16.7) | 5.5 | 35.6 (3.4,87.3) | 7.7 (0.8,18.5) | 16.6 (1.6,40.0) |
| **SGA** | 23.6 (20.1,27.6) | 10 | 102.3 (46.8,169.9) | 21.9 (10.3,37.1) | 47.5 (22.3,80.4) |

Impact of sub-optimal prevalences in low birthweight (LBW), preterm birth (PTB), or small for gestational age (SGA) on human capital and labor market outcomes in Burundi.

^1^ TMRED = Theoretical Minimum Risk Exposure Distribution (TMRED)

^2^ Source: United National Population Division World Population Prospects 2019.

^3^ Country specific annual wage data from World Indicators Database. Average yearly wage was estimated to be 2/3 of the gross domestic product in 2010 constant US dollars and 2011 International dollars, adjusted for purchasing power parity.

**CaboVerde**

**Super region:** Sub-Saharan Africa**; Sub region:** Western Sub-Saharan Africa

**Number of live births^3^:** 53,000

**Probability of survival to age 25^2^**: 0.96

**GDP per capita 2010 US dollars (estimated annual wage)^3^:**  3,415

**GDP per capita 2011 International dollars (estimated annual wage)^3^:**  6,007

| **Birth outcome** | **Current**  **prevalence (%)** | **TMRED^1^ (%)** | **Total school years gained per birth cohort**  **(in 1000s)** | **Increase in lifetime earnings in USD per child (20 to 59yrs)** | **Benefits by cohorts (20 to 59yrs)** **Lifetime wages**  **(in USD millions)** |
| --- | --- | --- | --- | --- | --- |
| **LBW** | 6.0 (0.7,11.3) | 3.2 | 0.4 (0.0,1.4) | 27.4 (0.0,106.1) | 1.5 (0.0,5.6) |
| **PTB** | 12.0 (8.6,16.7) | 5.5 | 1.0 (0.1,2.3) | 69.5 (8.8,171.5) | 3.7 (0.5,9.1) |
| **SGA** | 10.8 (7.6,14.6) | 10 | 0.1 (0.0,0.9) | 9.7 (0.0,68.2) | 0.5 (0.0,3.6) |

Impact of sub-optimal prevalences in low birthweight (LBW), preterm birth (PTB), or small for gestational age (SGA) on human capital and labor market outcomes in CaboVerde.

^1^ TMRED = Theoretical Minimum Risk Exposure Distribution (TMRED)

^2^ Source: United National Population Division World Population Prospects 2019.

^3^ Country specific annual wage data from World Indicators Database. Average yearly wage was estimated to be 2/3 of the gross domestic product in 2010 constant US dollars and 2011 International dollars, adjusted for purchasing power parity.

**Cambodia**

**Super region:** Southeast Asia, East Asia, and Oceania**; Sub region:** Southeast Asia

**Number of live births^3^:** 1,832,000

**Probability of survival to age 25^2^**: 0.94

**GDP per capita 2010 US dollars (estimated annual wage)^3^:**  1,025

**GDP per capita 2011 International dollars (estimated annual wage)^3^:**  3,290

| **Birth outcome** | **Current**  **prevalence (%)** | **TMRED^1^ (%)** | **Total school years gained per birth cohort**  **(in 1000s)** | **Increase in lifetime earnings in USD per child (20 to 59yrs)** | **Benefits by cohorts (20 to 59yrs)** **Lifetime wages**  **(in USD millions)** |
| --- | --- | --- | --- | --- | --- |
| **LBW** | 12.1 (7.6,16.4) | 3.2 | 42.5 (10.8,86.7) | 26.5 (4.1,67.8) | 48.6 (7.5,124.3) |
| **PTB** | 10.4 (8.7,11.9) | 5.5 | 25.9 (3.8,52.8) | 16.3 (1.6,42.4) | 29.9 (2.9,77.8) |
| **SGA** | 18.7 (13.8,25.3) | 10 | 60.0 (17.9,119.0) | 37.7 (6.7,98.3) | 69.1 (12.3,180.1) |

Impact of sub-optimal prevalences in low birthweight (LBW), preterm birth (PTB), or small for gestational age (SGA) on human capital and labor market outcomes in Cambodia.

^1^ TMRED = Theoretical Minimum Risk Exposure Distribution (TMRED)

^2^ Source: United National Population Division World Population Prospects 2019.

^3^ Country specific annual wage data from World Indicators Database. Average yearly wage was estimated to be 2/3 of the gross domestic product in 2010 constant US dollars and 2011 International dollars, adjusted for purchasing power parity.

**Cameroon**

**Super region:** Sub-Saharan Africa**; Sub region:** Western Sub-Saharan Africa

**Number of live births^3^:** 4,437,000

**Probability of survival to age 25^2^**: 0.84

**GDP per capita 2010 US dollars (estimated annual wage)^3^:**  1,440

**GDP per capita 2011 International dollars (estimated annual wage)^3^:**  3,224

| **Birth outcome** | **Current**  **prevalence (%)** | **TMRED^1^ (%)** | **Total school years gained per birth cohort**  **(in 1000s)** | **Increase in lifetime earnings in USD per child (20 to 59yrs)** | **Benefits by cohorts (20 to 59yrs)** **Lifetime wages**  **(in USD millions)** |
| --- | --- | --- | --- | --- | --- |
| **LBW** | 12.0 (9.3,15.2) | 3.2 | 92.5 (25.5,176.4) | 33.1 (8.8,69.5) | 147.0 (38.8,308.2) |
| **PTB** | 12.0 (8.6,16.7) | 5.5 | 72.3 (8.5,166.1) | 26.1 (3.1,64.1) | 115.8 (13.9,284.6) |
| **SGA** | 21.2 (17.6,24.8) | 10 | 171.0 (73.6,282.5) | 60.6 (24.1,110.9) | 268.8 (107.0,492.1) |

Impact of sub-optimal prevalences in low birthweight (LBW), preterm birth (PTB), or small for gestational age (SGA) on human capital and labor market outcomes in Cameroon.

^1^ TMRED = Theoretical Minimum Risk Exposure Distribution (TMRED)

^2^ Source: United National Population Division World Population Prospects 2019.

^3^ Country specific annual wage data from World Indicators Database. Average yearly wage was estimated to be 2/3 of the gross domestic product in 2010 constant US dollars and 2011 International dollars, adjusted for purchasing power parity.

**Central African Republic**

**Super region:** Sub-Saharan Africa**; Sub region:** Central Sub-Saharan Africa

**Number of live births^3^:** 825,000

**Probability of survival to age 25^2^**: 0.79

**GDP per capita 2010 US dollars (estimated annual wage)^3^:**  349

**GDP per capita 2011 International dollars (estimated annual wage)^3^:**  703

| **Birth outcome** | **Current**  **prevalence (%)** | **TMRED^1^ (%)** | **Total school years gained per birth cohort**  **(in 1000s)** | **Increase in lifetime earnings in USD per child (20 to 59yrs)** | **Benefits by cohorts (20 to 59yrs)** **Lifetime wages**  **(in USD millions)** |
| --- | --- | --- | --- | --- | --- |
| **LBW** | 14.5 (11.3,18.1) | 3.2 | 21.1 (6.4,38.1) | 15.2 (4.1,30.7) | 12.5 (3.4,25.3) |
| **PTB** | 12.0 (8.6,16.7) | 5.5 | 12.4 (1.6,30.2) | 9.2 (1.1,24.1) | 7.6 (0.9,19.9) |
| **SGA** | 27.0 (22.9,31.5) | 10 | 44.4 (18.9,73.1) | 32.6 (13.2,58.5) | 26.9 (10.9,48.3) |

Impact of sub-optimal prevalences in low birthweight (LBW), preterm birth (PTB), or small for gestational age (SGA) on human capital and labor market outcomes in Central African Republic.

^1^ TMRED = Theoretical Minimum Risk Exposure Distribution (TMRED)

^2^ Source: United National Population Division World Population Prospects 2019.

^3^ Country specific annual wage data from World Indicators Database. Average yearly wage was estimated to be 2/3 of the gross domestic product in 2010 constant US dollars and 2011 International dollars, adjusted for purchasing power parity.

**Chad**

**Super region:** Sub-Saharan Africa**; Sub region:** Western Sub-Saharan Africa

**Number of live births^3^:** 3,239,000

**Probability of survival to age 25^2^**: 0.78

**GDP per capita 2010 US dollars (estimated annual wage)^3^:**  956

**GDP per capita 2011 International dollars (estimated annual wage)^3^:**  2,052

| **Birth outcome** | **Current**  **prevalence (%)** | **TMRED^1^ (%)** | **Total school years gained per birth cohort**  **(in 1000s)** | **Increase in lifetime earnings in USD per child (20 to 59yrs)** | **Benefits by cohorts (20 to 59yrs)** **Lifetime wages**  **(in USD millions)** |
| --- | --- | --- | --- | --- | --- |
| **LBW** | 21.7 (16.4,27.1) | 3.2 | 132.8 (37.3,243.8) | 43.2 (10.7,84.4) | 140.0 (34.7,273.3) |
| **PTB** | 12.0 (8.6,16.7) | 5.5 | 47.2 (5.6,115.3) | 15.5 (2.0,40.0) | 50.3 (6.4,129.6) |
| **SGA** | 36.8 (31.6,42.5) | 10 | 272.9 (126.9,432.9) | 89.7 (39.6,153.9) | 290.4 (128.3,498.5) |

Impact of sub-optimal prevalences in low birthweight (LBW), preterm birth (PTB), or small for gestational age (SGA) on human capital and labor market outcomes in Chad.

^1^ TMRED = Theoretical Minimum Risk Exposure Distribution (TMRED)

^2^ Source: United National Population Division World Population Prospects 2019.

^3^ Country specific annual wage data from World Indicators Database. Average yearly wage was estimated to be 2/3 of the gross domestic product in 2010 constant US dollars and 2011 International dollars, adjusted for purchasing power parity.

**China**

**Super region:** Southeast Asia, East Asia, and Oceania**; Sub region:** East Asia

**Number of live births^3^:** 84,890,000

**Probability of survival to age 25^2^**: 0.98

**GDP per capita 2010 US dollars (estimated annual wage)^3^:**  6,484

**GDP per capita 2011 International dollars (estimated annual wage)^3^:**  13,535

| **Birth outcome** | **Current**  **prevalence (%)** | **TMRED^1^ (%)** | **Total school years gained per birth cohort**  **(in 1000s)** | **Increase in lifetime earnings in USD per child (20 to 59yrs)** | **Benefits by cohorts (20 to 59yrs)** **Lifetime wages**  **(in USD millions)** |
| --- | --- | --- | --- | --- | --- |
| **LBW** | 5.0 (3.9,6.4) | 3.2 | 404.7 (80.3,940.4) | 23.0 (3.9,54.9) | 1953.9 (335.2,4660.9) |
| **PTB** | 6.9 (5.8,7.9) | 5.5 | 333.4 (19.0,854.0) | 18.6 (1.1,48.6) | 1581.5 (95.8,4125.9) |
| **SGA** | 6.5 (3.8,10.7) | 10 | 0.0 (0.0,0.0) | 0.0 (0.0,0.0) | 0.0 (0.0,0.0) |

Impact of sub-optimal prevalences in low birthweight (LBW), preterm birth (PTB), or small for gestational age (SGA) on human capital and labor market outcomes in China.

^1^ TMRED = Theoretical Minimum Risk Exposure Distribution (TMRED)

^2^ Source: United National Population Division World Population Prospects 2019.

^3^ Country specific annual wage data from World Indicators Database. Average yearly wage was estimated to be 2/3 of the gross domestic product in 2010 constant US dollars and 2011 International dollars, adjusted for purchasing power parity.

**Colombia**

**Super region:** Latin America and Caribbean**; Sub region:** Central Latin America

**Number of live births^3^:** 3,697,000

**Probability of survival to age 25^2^**: 0.96

**GDP per capita 2010 US dollars (estimated annual wage)^3^:**  7,572

**GDP per capita 2011 International dollars (estimated annual wage)^3^:**  13,115

| **Birth outcome** | **Current**  **prevalence (%)** | **TMRED^1^ (%)** | **Total school years gained per birth cohort**  **(in 1000s)** | **Increase in lifetime earnings in USD per child (20 to 59yrs)** | **Benefits by cohorts (20 to 59yrs)** **Lifetime wages**  **(in USD millions)** |
| --- | --- | --- | --- | --- | --- |
| **LBW** | 10.0 (7.7,13.2) | 3.2 | 66.6 (17.3,131.4) | 232.8 (60.9,478.8) | 860.7 (225.0,1770.0) |
| **PTB** | 14.5 (11.4,18.2) | 5.5 | 96.5 (12.9,202.8) | 338.7 (43.4,745.9) | 1252.2 (160.5,2757.6) |
| **SGA** | 14.2 (10.7,18.3) | 10 | 57.8 (3.6,132.7) | 199.8 (12.8,480.1) | 738.8 (47.2,1775.0) |

Impact of sub-optimal prevalences in low birthweight (LBW), preterm birth (PTB), or small for gestational age (SGA) on human capital and labor market outcomes in Colombia.

^1^ TMRED = Theoretical Minimum Risk Exposure Distribution (TMRED)

^2^ Source: United National Population Division World Population Prospects 2019.

^3^ Country specific annual wage data from World Indicators Database. Average yearly wage was estimated to be 2/3 of the gross domestic product in 2010 constant US dollars and 2011 International dollars, adjusted for purchasing power parity.

**Comoros**

**Super region:** Sub-Saharan Africa**; Sub region:** Eastern Sub-Saharan Africa

**Number of live births^3^:** 132,000

**Probability of survival to age 25^2^**: 0.89

**GDP per capita 2010 US dollars (estimated annual wage)^3^:**  1,352

**GDP per capita 2011 International dollars (estimated annual wage)^3^:**  2,494

| **Birth outcome** | **Current**  **prevalence (%)** | **TMRED^1^ (%)** | **Total school years gained per birth cohort**  **(in 1000s)** | **Increase in lifetime earnings in USD per child (20 to 59yrs)** | **Benefits by cohorts (20 to 59yrs)** **Lifetime wages**  **(in USD millions)** |
| --- | --- | --- | --- | --- | --- |
| **LBW** | 23.7 (18.9,29.8) | 3.2 | 6.8 (2.0,13.2) | 140.4 (43.1,281.2) | 18.5 (5.7,37.1) |
| **PTB** | 12.0 (8.6,16.7) | 5.5 | 2.2 (0.3,5.4) | 46.5 (6.7,114.3) | 6.1 (0.9,15.1) |
| **SGA** | 37.6 (30.8,43.8) | 10 | 12.9 (6.0,21.2) | 270.8 (120.3,466.1) | 35.7 (15.9,61.5) |

Impact of sub-optimal prevalences in low birthweight (LBW), preterm birth (PTB), or small for gestational age (SGA) on human capital and labor market outcomes in Comoros.

^1^ TMRED = Theoretical Minimum Risk Exposure Distribution (TMRED)

^2^ Source: United National Population Division World Population Prospects 2019.

^3^ Country specific annual wage data from World Indicators Database. Average yearly wage was estimated to be 2/3 of the gross domestic product in 2010 constant US dollars and 2011 International dollars, adjusted for purchasing power parity.

**Congo**

**Super region:** Sub-Saharan Africa**; Sub region:** Central Sub-Saharan Africa

**Number of live births^3^:** 857,000

**Probability of survival to age 25^2^**: 0.91

**GDP per capita 2010 US dollars (estimated annual wage)^3^:**  3,010

**GDP per capita 2011 International dollars (estimated annual wage)^3^:**  5,248

| **Birth outcome** | **Current**  **prevalence (%)** | **TMRED^1^ (%)** | **Total school years gained per birth cohort**  **(in 1000s)** | **Increase in lifetime earnings in USD per child (20 to 59yrs)** | **Benefits by cohorts (20 to 59yrs)** **Lifetime wages**  **(in USD millions)** |
| --- | --- | --- | --- | --- | --- |
| **LBW** | 13.1 (8.3,17.9) | 3.2 | 21.7 (5.4,40.8) | 132.0 (32.3,265.6) | 113.1 (27.7,227.7) |
| **PTB** | 12.0 (8.6,16.7) | 5.5 | 15.3 (1.5,36.1) | 94.8 (9.6,234.0) | 81.2 (8.2,200.5) |
| **SGA** | 24.6 (20.5,28.2) | 10 | 46.3 (19.3,74.0) | 282.5 (115.9,506.7) | 242.1 (99.3,434.2) |

Impact of sub-optimal prevalences in low birthweight (LBW), preterm birth (PTB), or small for gestational age (SGA) on human capital and labor market outcomes in Congo.

^1^ TMRED = Theoretical Minimum Risk Exposure Distribution (TMRED)

^2^ Source: United National Population Division World Population Prospects 2019.

^3^ Country specific annual wage data from World Indicators Database. Average yearly wage was estimated to be 2/3 of the gross domestic product in 2010 constant US dollars and 2011 International dollars, adjusted for purchasing power parity.

**Costa Rica**

**Super region:** Latin America and Caribbean**; Sub region:** Central Latin America

**Number of live births^3^:** 351,000

**Probability of survival to age 25^2^**: 0.98

**GDP per capita 2010 US dollars (estimated annual wage)^3^:**  9,219

**GDP per capita 2011 International dollars (estimated annual wage)^3^:**  14,617

| **Birth outcome** | **Current**  **prevalence (%)** | **TMRED^1^ (%)** | **Total school years gained per birth cohort**  **(in 1000s)** | **Increase in lifetime earnings in USD per child (20 to 59yrs)** | **Benefits by cohorts (20 to 59yrs)** **Lifetime wages**  **(in USD millions)** |
| --- | --- | --- | --- | --- | --- |
| **LBW** | 7.5 (7.4,7.5) | 3.2 | 4.3 (1.3,7.1) | 189.6 (56.6,332.7) | 66.5 (19.9,116.8) |
| **PTB** | 9.8 (8.6,11.3) | 5.5 | 4.5 (0.7,9.3) | 195.4 (32.2,429.3) | 68.6 (11.3,150.7) |
| **SGA** | 11.3 (7.7,15.3) | 10 | 1.7 (0.0,8.1) | 75.0 (0.0,383.0) | 26.3 (0.0,134.4) |

Impact of sub-optimal prevalences in low birthweight (LBW), preterm birth (PTB), or small for gestational age (SGA) on human capital and labor market outcomes in Costa Rica.

^1^ TMRED = Theoretical Minimum Risk Exposure Distribution (TMRED)

^2^ Source: United National Population Division World Population Prospects 2019.

^3^ Country specific annual wage data from World Indicators Database. Average yearly wage was estimated to be 2/3 of the gross domestic product in 2010 constant US dollars and 2011 International dollars, adjusted for purchasing power parity.

**Cote d’lvoire**

**Super region:** Sub-Saharan Africa**; Sub region:** Western Sub-Saharan Africa

**Number of live births^3^:** 4,451,000

**Probability of survival to age 25^2^**: 0.84

**GDP per capita 2010 US dollars (estimated annual wage)^3^:**  1,462

**GDP per capita 2011 International dollars (estimated annual wage)^3^:**  3,225

| **Birth outcome** | **Current**  **prevalence (%)** | **TMRED^1^ (%)** | **Total school years gained per birth cohort**  **(in 1000s)** | **Increase in lifetime earnings in USD per child (20 to 59yrs)** | **Benefits by cohorts (20 to 59yrs)** **Lifetime wages**  **(in USD millions)** |
| --- | --- | --- | --- | --- | --- |
| **LBW** | 15.5 (13.7,22.4) | 3.2 | 129.9 (37.1,241.5) | 51.4 (9.9,112.6) | 228.9 (44.0,501.0) |
| **PTB** | 12.0 (8.6,16.7) | 5.5 | 71.1 (8.3,171.2) | 27.5 (3.7,78.1) | 122.5 (16.3,347.5) |
| **SGA** | 31.0 (26.7,35.4) | 10 | 318.0 (143.9,502.1) | 124.6 (47.5,234.5) | 554.4 (211.6,1044.0) |

Impact of sub-optimal prevalences in low birthweight (LBW), preterm birth (PTB), or small for gestational age (SGA) on human capital and labor market outcomes in Cote d’lvoire.

^1^ TMRED = Theoretical Minimum Risk Exposure Distribution (TMRED)

^2^ Source: United National Population Division World Population Prospects 2019.

^3^ Country specific annual wage data from World Indicators Database. Average yearly wage was estimated to be 2/3 of the gross domestic product in 2010 constant US dollars and 2011 International dollars, adjusted for purchasing power parity.

**Cuba**

**Super region:** Latin America and Caribbean**; Sub region:** Caribbean

**Number of live births^3^:** 579,000

**Probability of survival to age 25^2^**: 0.98

**GDP per capita 2010 US dollars (estimated annual wage)^3^:**  6,523

**GDP per capita 2011 International dollars (estimated annual wage)^3^:**  18,083

| **Birth outcome** | **Current**  **prevalence (%)** | **TMRED^1^ (%)** | **Total school years gained per birth cohort**  **(in 1000s)** | **Increase in lifetime earnings in USD per child (20 to 59yrs)** | **Benefits by cohorts (20 to 59yrs)** **Lifetime wages**  **(in USD millions)** |
| --- | --- | --- | --- | --- | --- |
| **LBW** | 5.3 (5.2,5.4) | 3.2 | 3.5 (1.0,5.9) | 69.5 (21.0,120.1) | 40.3 (12.1,69.5) |
| **PTB** | 6.0 (1.9,10.1) | 5.5 | 0.4 (0.0,9.7) | 7.5 (0.0,193.8) | 4.3 (0.0,112.2) |
| **SGA** | 8.4 (5.7,11.9) | 10 | 0.0 (0.0,3.8) | 0.0 (0.0,67.1) | 0.0 (0.0,38.9) |

Impact of sub-optimal prevalences in low birthweight (LBW), preterm birth (PTB), or small for gestational age (SGA) on human capital and labor market outcomes in Cuba.

^1^ TMRED = Theoretical Minimum Risk Exposure Distribution (TMRED)

^2^ Source: United National Population Division World Population Prospects 2019.

^3^ Country specific annual wage data from World Indicators Database. Average yearly wage was estimated to be 2/3 of the gross domestic product in 2010 constant US dollars and 2011 International dollars, adjusted for purchasing power parity.

**Djibouti**

**Super region:** Sub-Saharan Africa**; Sub region:** Eastern Sub-Saharan Africa

**Number of live births^3^:** 103,000

**Probability of survival to age 25^2^**: 0.91

**GDP per capita 2010 US dollars (estimated annual wage)^3^:**  1,343

**GDP per capita 2011 International dollars (estimated annual wage)^3^:**  10,215

| **Birth outcome** | **Current**  **prevalence (%)** | **TMRED^1^ (%)** | **Total school years gained per birth cohort**  **(in 1000s)** | **Increase in lifetime earnings in USD per child (20 to 59yrs)** | **Benefits by cohorts (20 to 59yrs)** **Lifetime wages**  **(in USD millions)** |
| --- | --- | --- | --- | --- | --- |
| **LBW** | 20.0 (14.4,25.6) | 3.2 | 4.4 (1.2,8.2) | 115.1 (32.4,220.3) | 11.9 (3.3,22.7) |
| **PTB** | 12.0 (8.6,16.7) | 5.5 | 1.7 (0.2,4.2) | 45.2 (6.0,115.4) | 4.7 (0.6,11.9) |
| **SGA** | 32.3 (27.0,37.0) | 10 | 8.3 (4.0,14.0) | 220.7 (102.2,370.7) | 22.7 (10.5,38.2) |

Impact of sub-optimal prevalences in low birthweight (LBW), preterm birth (PTB), or small for gestational age (SGA) on human capital and labor market outcomes in Djibouti.

^1^ TMRED = Theoretical Minimum Risk Exposure Distribution (TMRED)

^2^ Source: United National Population Division World Population Prospects 2019.

^3^ Country specific annual wage data from World Indicators Database. Average yearly wage was estimated to be 2/3 of the gross domestic product in 2010 constant US dollars and 2011 International dollars, adjusted for purchasing power parity.

**Dominican Republic**

**Super region:** Latin America and Caribbean**; Sub region:** Caribbean

**Number of live births^3^:** 1,040,000

**Probability of survival to age 25^2^**: 0.94

**GDP per capita 2010 US dollars (estimated annual wage)^3^:**  6,720

**GDP per capita 2011 International dollars (estimated annual wage)^3^:**  13,717

| **Birth outcome** | **Current**  **prevalence (%)** | **TMRED^1^ (%)** | **Total school years gained per birth cohort**  **(in 1000s)** | **Increase in lifetime earnings in USD per child (20 to 59yrs)** | **Benefits by cohorts (20 to 59yrs)** **Lifetime wages**  **(in USD millions)** |
| --- | --- | --- | --- | --- | --- |
| **LBW** | 11.3 (8.7,15.0) | 3.2 | 22.2 (6.1,42.5) | 251.7 (66.5,490.4) | 261.7 (69.2,510.0) |
| **PTB** | 9.8 (8.6,11.3) | 5.5 | 13.0 (1.9,27.2) | 147.9 (21.4,308.8) | 153.8 (22.3,321.1) |
| **SGA** | 13.4 (9.9,18.0) | 10 | 12.8 (0.0,34.7) | 147.1 (0.0,390.8) | 153.0 (0.0,406.4) |

Impact of sub-optimal prevalences in low birthweight (LBW), preterm birth (PTB), or small for gestational age (SGA) on human capital and labor market outcomes in Dominican Republic  .

^1^ TMRED = Theoretical Minimum Risk Exposure Distribution (TMRED)

^2^ Source: United National Population Division World Population Prospects 2019.

^3^ Country specific annual wage data from World Indicators Database. Average yearly wage was estimated to be 2/3 of the gross domestic product in 2010 constant US dollars and 2011 International dollars, adjusted for purchasing power parity.

**Democratic Republic of Congo**

**Super region:** Sub-Saharan Africa**; Sub region:** Central Sub-Saharan Africa

**Number of live births^3^:** 17,169,000

**Probability of survival to age 25^2^**: 0.83

**GDP per capita 2010 US dollars (estimated annual wage)^3^:**  411

**GDP per capita 2011 International dollars (estimated annual wage)^3^:**  812

| **Birth outcome** | **Current**  **prevalence (%)** | **TMRED^1^ (%)** | **Total school years gained per birth cohort**  **(in 1000s)** | **Increase in lifetime earnings in USD per child (20 to 59yrs)** | **Benefits by cohorts (20 to 59yrs)** **Lifetime wages**  **(in USD millions)** |
| --- | --- | --- | --- | --- | --- |
| **LBW** | 10.8 (8.5,15.1) | 3.2 | 309.2 (87.6,587.5) | 13.0 (3.6,27.7) | 223.4 (61.1,475.9) |
| **PTB** | 9.8 (7.1,13.2) | 5.5 | 178.8 (20.9,464.1) | 7.6 (0.8,20.3) | 129.6 (13.4,349.3) |
| **SGA** | 21.9 (18.0,26.0) | 10 | 684.5 (295.5,1199.6) | 28.6 (12.5,54.2) | 491.1 (214.1,930.2) |

Impact of sub-optimal prevalences in low birthweight (LBW), preterm birth (PTB), or small for gestational age (SGA) on human capital and labor market outcomes in Democratic Republic of Congo.

^1^ TMRED = Theoretical Minimum Risk Exposure Distribution (TMRED)

^2^ Source: United National Population Division World Population Prospects 2019.

^3^ Country specific annual wage data from World Indicators Database. Average yearly wage was estimated to be 2/3 of the gross domestic product in 2010 constant US dollars and 2011 International dollars, adjusted for purchasing power parity.

**Democratic Republic of Korea**

**Super region:** Southeast Asia, East Asia, and Oceania**; Sub region:** East Asia

**Number of live births^3^:** 1,776,000

**Probability of survival to age 25^2^**: 0.96

**GDP per capita 2010 US dollars (estimated annual wage)^3^:**  523

**GDP per capita 2011 International dollars (estimated annual wage)^3^:**  1,559

| **Birth outcome** | **Current**  **prevalence (%)** | **TMRED^1^ (%)** | **Total school years gained per birth cohort**  **(in 1000s)** | **Increase in lifetime earnings in USD per child (20 to 59yrs)** | **Benefits by cohorts (20 to 59yrs)** **Lifetime wages**  **(in USD millions)** |
| --- | --- | --- | --- | --- | --- |
| **LBW** | 6.7 (5.5,8.0) | 3.2 | 16.7 (4.8,31.5) | 3.7 (1.0,7.2) | 6.5 (1.8,12.9) |
| **PTB** | 10.4 (8.7,11.9) | 5.5 | 25.5 (3.5,52.0) | 5.6 (0.8,11.9) | 9.9 (1.4,21.1) |
| **SGA** | 16.0 (11.1,22.4) | 10 | 38.7 (0.0,90.4) | 8.6 (0.0,20.4) | 15.2 (0.0,36.2) |

Impact of sub-optimal prevalences in low birthweight (LBW), preterm birth (PTB), or small for gestational age (SGA) on human capital and labor market outcomes in Democratic Republic of Korea.

^1^ TMRED = Theoretical Minimum Risk Exposure Distribution (TMRED)

^2^ Source: United National Population Division World Population Prospects 2019.

^3^ Country specific annual wage data from World Indicators Database. Average yearly wage was estimated to be 2/3 of the gross domestic product in 2010 constant US dollars and 2011 International dollars, adjusted for purchasing power parity.

**Ecuador**

**Super region:** Latin America and Caribbean**; Sub region:** Andean Latin America

**Number of live births^3^:** 1,681,000

**Probability of survival to age 25^2^**: 0.96

**GDP per capita 2010 US dollars (estimated annual wage)^3^:**  5,331

**GDP per capita 2011 International dollars (estimated annual wage)^3^:**  10,704

| **Birth outcome** | **Current**  **prevalence (%)** | **TMRED^1^ (%)** | **Total school years gained per birth cohort**  **(in 1000s)** | **Increase in lifetime earnings in USD per child (20 to 59yrs)** | **Benefits by cohorts (20 to 59yrs)** **Lifetime wages**  **(in USD millions)** |
| --- | --- | --- | --- | --- | --- |
| **LBW** | 11.2 (8.7,14.2) | 3.2 | 36.5 (9.6,68.9) | 139.7 (37.3,274.7) | 234.8 (62.7,461.7) |
| **PTB** | 8.0 (4.3,13.3) | 5.5 | 10.7 (0.0,43.2) | 40.6 (0.0,169.1) | 68.3 (0.0,284.2) |
| **SGA** | 9.7 (6.9,13.7) | 10 | 0.0 (0.0,20.8) | 0.0 (0.0,82.2) | 0.0 (0.0,138.2) |

Impact of sub-optimal prevalences in low birthweight (LBW), preterm birth (PTB), or small for gestational age (SGA) on human capital and labor market outcomes in Ecuador.

^1^ TMRED = Theoretical Minimum Risk Exposure Distribution (TMRED)

^2^ Source: United National Population Division World Population Prospects 2019.

^3^ Country specific annual wage data from World Indicators Database. Average yearly wage was estimated to be 2/3 of the gross domestic product in 2010 constant US dollars and 2011 International dollars, adjusted for purchasing power parity.

**Egypt**

**Super region:** North Africa and Middle East**; Sub region:** North Africa and Middle East

**Number of live births^3^:** 12,922,000

**Probability of survival to age 25^2^**: 0.97

**GDP per capita 2010 US dollars (estimated annual wage)^3^:**  2,704

**GDP per capita 2011 International dollars (estimated annual wage)^3^:**  10,243

| **Birth outcome** | **Current**  **prevalence (%)** | **TMRED^1^ (%)** | **Total school years gained per birth cohort**  **(in 1000s)** | **Increase in lifetime earnings in USD per child (20 to 59yrs)** | **Benefits by cohorts (20 to 59yrs)** **Lifetime wages**  **(in USD millions)** |
| --- | --- | --- | --- | --- | --- |
| **LBW** | 6.0 (2.0,10.0) | 3.2 | 90.3 (0.0,290.5) | 17.7 (0.0,56.1) | 228.2 (0.0,724.6) |
| **PTB** | 13.4 (6.3,30.9) | 5.5 | 283.0 (0.0,1012.2) | 55.5 (0.0,198.8) | 717.1 (0.0,2569.5) |
| **SGA** | 9.9 (6.8,13.4) | 10 | 0.0 (0.0,170.4) | 0.0 (0.0,32.8) | 0.0 (0.0,424.4) |

Impact of sub-optimal prevalences in low birthweight (LBW), preterm birth (PTB), or small for gestational age (SGA) on human capital and labor market outcomes in Egypt.

^1^ TMRED = Theoretical Minimum Risk Exposure Distribution (TMRED)

^2^ Source: United National Population Division World Population Prospects 2019.

^3^ Country specific annual wage data from World Indicators Database. Average yearly wage was estimated to be 2/3 of the gross domestic product in 2010 constant US dollars and 2011 International dollars, adjusted for purchasing power parity.

**ElSalvador**

**Super region:** Latin America and Caribbean**; Sub region:** Central Latin America

**Number of live births^3^:** 589,000

**Probability of survival to age 25^2^**: 0.94

**GDP per capita 2010 US dollars (estimated annual wage)^3^:**  3,315

**GDP per capita 2011 International dollars (estimated annual wage)^3^:**  6,979

| **Birth outcome** | **Current**  **prevalence (%)** | **TMRED^1^ (%)** | **Total school years gained per birth cohort**  **(in 1000s)** | **Increase in lifetime earnings in USD per child (20 to 59yrs)** | **Benefits by cohorts (20 to 59yrs)** **Lifetime wages**  **(in USD millions)** |
| --- | --- | --- | --- | --- | --- |
| **LBW** | 10.3 (8.0,13.3) | 3.2 | 11.0 (2.8,20.9) | 105.6 (29.1,211.9) | 62.2 (17.2,124.8) |
| **PTB** | 9.6 (7.4,12.1) | 5.5 | 6.6 (0.8,16.2) | 63.5 (8.2,154.9) | 37.4 (4.8,91.3) |
| **SGA** | 15.3 (11.2,20.8) | 10 | 11.6 (0.8,26.4) | 114.9 (8.8,256.3) | 67.7 (5.2,150.9) |

Impact of sub-optimal prevalences in low birthweight (LBW), preterm birth (PTB), or small for gestational age (SGA) on human capital and labor market outcomes in ElSalvador.

^1^ TMRED = Theoretical Minimum Risk Exposure Distribution (TMRED)

^2^ Source: United National Population Division World Population Prospects 2019.

^3^ Country specific annual wage data from World Indicators Database. Average yearly wage was estimated to be 2/3 of the gross domestic product in 2010 constant US dollars and 2011 International dollars, adjusted for purchasing power parity.

**Equatorial Guinea**

**Super region:** Sub-Saharan Africa**; Sub region:** Central Sub-Saharan Africa

**Number of live births^3^:** 215,000

**Probability of survival to age 25^2^**: 0.84

**GDP per capita 2010 US dollars (estimated annual wage)^3^:**  14,080

**GDP per capita 2011 International dollars (estimated annual wage)^3^:**  27,709

| **Birth outcome** | **Current**  **prevalence (%)** | **TMRED^1^ (%)** | **Total school years gained per birth cohort**  **(in 1000s)** | **Increase in lifetime earnings in USD per child (20 to 59yrs)** | **Benefits by cohorts (20 to 59yrs)** **Lifetime wages**  **(in USD millions)** |
| --- | --- | --- | --- | --- | --- |
| **LBW** | 13.6 (8.8,18.4) | 3.2 | 5.3 (1.4,10.3) | 613.7 (172.3,1276.0) | 132.0 (37.1,274.3) |
| **PTB** | 11.7 (10.6,12.8) | 5.5 | 3.5 (0.5,6.6) | 403.4 (55.4,816.9) | 86.7 (11.9,175.6) |
| **SGA** | 25.5 (21.5,29.6) | 10 | 11.5 (5.2,18.7) | 1323.8 (541.9,2354.2) | 284.6 (116.5,506.2) |

Impact of sub-optimal prevalences in low birthweight (LBW), preterm birth (PTB), or small for gestational age (SGA) on human capital and labor market outcomes in Equatorial Guinea.

^1^ TMRED = Theoretical Minimum Risk Exposure Distribution (TMRED)

^2^ Source: United National Population Division World Population Prospects 2019.

^3^ Country specific annual wage data from World Indicators Database. Average yearly wage was estimated to be 2/3 of the gross domestic product in 2010 constant US dollars and 2011 International dollars, adjusted for purchasing power parity.

**Eritrea**

**Super region:** Sub-Saharan Africa**; Sub region:** Eastern Sub-Saharan Africa

**Number of live births^3^:** 528,000

**Probability of survival to age 25^2^**: 0.92

**GDP per capita 2010 US dollars (estimated annual wage)^3^:**  668

**GDP per capita 2011 International dollars (estimated annual wage)^3^:**  1,962

| **Birth outcome** | **Current**  **prevalence (%)** | **TMRED^1^ (%)** | **Total school years gained per birth cohort**  **(in 1000s)** | **Increase in lifetime earnings in USD per child (20 to 59yrs)** | **Benefits by cohorts (20 to 59yrs)** **Lifetime wages**  **(in USD millions)** |
| --- | --- | --- | --- | --- | --- |
| **LBW** | 14.1 (8.5,19.7) | 3.2 | 14.4 (3.5,30.5) | 37.5 (9.3,79.8) | 19.8 (4.9,42.1) |
| **PTB** | 12.0 (8.6,16.7) | 5.5 | 9.5 (1.0,21.8) | 24.0 (2.4,56.0) | 12.7 (1.3,29.6) |
| **SGA** | 23.1 (18.2,27.5) | 10 | 25.5 (11.3,46.0) | 64.8 (28.4,119.9) | 34.2 (15.0,63.3) |

Impact of sub-optimal prevalences in low birthweight (LBW), preterm birth (PTB), or small for gestational age (SGA) on human capital and labor market outcomes in Eritrea.

^1^ TMRED = Theoretical Minimum Risk Exposure Distribution (TMRED)

^2^ Source: United National Population Division World Population Prospects 2019.

^3^ Country specific annual wage data from World Indicators Database. Average yearly wage was estimated to be 2/3 of the gross domestic product in 2010 constant US dollars and 2011 International dollars, adjusted for purchasing power parity.

**Eswatini**

**Super region:** Sub-Saharan Africa**; Sub region:** Southern Sub-Saharan Africa

**Number of live births^3^:** 151,000

**Probability of survival to age 25^2^**: 0.89

**GDP per capita 2010 US dollars (estimated annual wage)^3^:**  4,688

**GDP per capita 2011 International dollars (estimated annual wage)^3^:**  9,269

| **Birth outcome** | **Current**  **prevalence (%)** | **TMRED^1^ (%)** | **Total school years gained per birth cohort**  **(in 1000s)** | **Increase in lifetime earnings in USD per child (20 to 59yrs)** | **Benefits by cohorts (20 to 59yrs)** **Lifetime wages**  **(in USD millions)** |
| --- | --- | --- | --- | --- | --- |
| **LBW** | 10.3 (7.9,13.7) | 3.2 | 2.7 (0.7,5.3) | 240.0 (62.9,465.5) | 36.2 (9.5,70.3) |
| **PTB** | 12.1 (10.2,13.9) | 5.5 | 2.8 (0.4,5.5) | 250.0 (32.5,488.6) | 37.7 (4.9,73.8) |
| **SGA** | 19.2 (15.9,23.0) | 10 | 5.0 (2.2,8.5) | 447.1 (201.4,766.4) | 67.5 (30.4,115.7) |

Impact of sub-optimal prevalences in low birthweight (LBW), preterm birth (PTB), or small for gestational age (SGA) on human capital and labor market outcomes in Eswatini.

^1^ TMRED = Theoretical Minimum Risk Exposure Distribution (TMRED)

^2^ Source: United National Population Division World Population Prospects 2019.

^3^ Country specific annual wage data from World Indicators Database. Average yearly wage was estimated to be 2/3 of the gross domestic product in 2010 constant US dollars and 2011 International dollars, adjusted for purchasing power parity.

**Ethiopia**

**Super region:** Sub-Saharan Africa**; Sub region:** Eastern Sub-Saharan Africa

**Number of live births^3^:** 17,572,000

**Probability of survival to age 25^2^**: 0.90

**GDP per capita 2010 US dollars (estimated annual wage)^3^:**  483

**GDP per capita 2011 International dollars (estimated annual wage)^3^:**  1,518

| **Birth outcome** | **Current**  **prevalence (%)** | **TMRED^1^ (%)** | **Total school years gained per birth cohort**  **(in 1000s)** | **Increase in lifetime earnings in USD per child (20 to 59yrs)** | **Benefits by cohorts (20 to 59yrs)** **Lifetime wages**  **(in USD millions)** |
| --- | --- | --- | --- | --- | --- |
| **LBW** | 20.3 (14.7,25.9) | 3.2 | 746.9 (218.4,1412.7) | 44.7 (12.6,92.6) | 785.6 (221.8,1628.0) |
| **PTB** | 12.0 (8.6,16.7) | 5.5 | 303.4 (41.8,739.2) | 18.3 (2.3,44.6) | 321.4 (41.2,783.9) |
| **SGA** | 32.1 (26.9,36.8) | 10 | 1412.8 (668.8,2413.1) | 85.6 (36.6,156.7) | 1504.3 (642.9,2752.8) |

Impact of sub-optimal prevalences in low birthweight (LBW), preterm birth (PTB), or small for gestational age (SGA) on human capital and labor market outcomes in Ethiopia.

^1^ TMRED = Theoretical Minimum Risk Exposure Distribution (TMRED)

^2^ Source: United National Population Division World Population Prospects 2019.

^3^ Country specific annual wage data from World Indicators Database. Average yearly wage was estimated to be 2/3 of the gross domestic product in 2010 constant US dollars and 2011 International dollars, adjusted for purchasing power parity.

**Fiji**

**Super region:** Southeast Asia, East Asia, and Oceania**; Sub region:** Oceania

**Number of live births^3^:** 95,000

**Probability of survival to age 25^2^**: 0.94

**GDP per capita 2010 US dollars (estimated annual wage)^3^:**  4,359

**GDP per capita 2011 International dollars (estimated annual wage)^3^:**  8,776

| **Birth outcome** | **Current**  **prevalence (%)** | **TMRED^1^ (%)** | **Total school years gained per birth cohort**  **(in 1000s)** | **Increase in lifetime earnings in USD per child (20 to 59yrs)** | **Benefits by cohorts (20 to 59yrs)** **Lifetime wages**  **(in USD millions)** |
| --- | --- | --- | --- | --- | --- |
| **LBW** | 8.1 (5.5,10.8) | 3.2 | 1.2 (0.3,2.6) | 58.8 (12.1,151.5) | 5.6 (1.2,14.4) |
| **PTB** | 10.0 (9.2,10.8) | 5.5 | 1.3 (0.2,2.4) | 60.4 (7.0,142.8) | 5.7 (0.7,13.6) |
| **SGA** | 18.0 (13.0,24.5) | 10 | 2.8 (0.7,6.3) | 138.9 (25.4,360.2) | 13.2 (2.4,34.2) |

Impact of sub-optimal prevalences in low birthweight (LBW), preterm birth (PTB), or small for gestational age (SGA) on human capital and labor market outcomes in Fiji.

^1^ TMRED = Theoretical Minimum Risk Exposure Distribution (TMRED)

^2^ Source: United National Population Division World Population Prospects 2019.

^3^ Country specific annual wage data from World Indicators Database. Average yearly wage was estimated to be 2/3 of the gross domestic product in 2010 constant US dollars and 2011 International dollars, adjusted for purchasing power parity.

**Gabon**

**Super region:** Sub-Saharan Africa**; Sub region:** Central Sub-Saharan Africa

**Number of live births^3^:** 334,000

**Probability of survival to age 25^2^**: 0.91

**GDP per capita 2010 US dollars (estimated annual wage)^3^:**  9,512

**GDP per capita 2011 International dollars (estimated annual wage)^3^:**  16,685

| **Birth outcome** | **Current**  **prevalence (%)** | **TMRED^1^ (%)** | **Total school years gained per birth cohort**  **(in 1000s)** | **Increase in lifetime earnings in USD per child (20 to 59yrs)** | **Benefits by cohorts (20 to 59yrs)** **Lifetime wages**  **(in USD millions)** |
| --- | --- | --- | --- | --- | --- |
| **LBW** | 14.2 (11.2,18.8) | 3.2 | 9.3 (2.8,18.1) | 460.6 (137.7,939.6) | 153.8 (46.0,313.8) |
| **PTB** | 12.0 (8.6,16.7) | 5.5 | 5.8 (0.8,13.7) | 288.0 (33.8,699.4) | 96.2 (11.3,233.6) |
| **SGA** | 24.6 (19.7,28.7) | 10 | 17.8 (8.0,30.0) | 892.0 (367.8,1609.9) | 297.9 (122.8,537.7) |

Impact of sub-optimal prevalences in low birthweight (LBW), preterm birth (PTB), or small for gestational age (SGA) on human capital and labor market outcomes in Gabon.

^1^ TMRED = Theoretical Minimum Risk Exposure Distribution (TMRED)

^2^ Source: United National Population Division World Population Prospects 2019.

^3^ Country specific annual wage data from World Indicators Database. Average yearly wage was estimated to be 2/3 of the gross domestic product in 2010 constant US dollars and 2011 International dollars, adjusted for purchasing power parity.

**Gambia**

**Super region:** Sub-Saharan Africa**; Sub region:** Western Sub-Saharan Africa

**Number of live births^3^:** 436,000

**Probability of survival to age 25^2^**: 0.87

**GDP per capita 2010 US dollars (estimated annual wage)^3^:**  507

**GDP per capita 2011 International dollars (estimated annual wage)^3^:**  1,481

| **Birth outcome** | **Current**  **prevalence (%)** | **TMRED^1^ (%)** | **Total school years gained per birth cohort**  **(in 1000s)** | **Increase in lifetime earnings in USD per child (20 to 59yrs)** | **Benefits by cohorts (20 to 59yrs)** **Lifetime wages**  **(in USD millions)** |
| --- | --- | --- | --- | --- | --- |
| **LBW** | 16.8 (13.5,21.0) | 3.2 | 14.5 (4.2,27.4) | 20.6 (5.7,40.5) | 9.0 (2.5,17.7) |
| **PTB** | 12.0 (8.6,16.7) | 5.5 | 7.1 (0.9,17.1) | 10.1 (1.3,25.5) | 4.4 (0.6,11.1) |
| **SGA** | 32.8 (27.5,37.8) | 10 | 34.8 (15.4,58.0) | 49.3 (21.7,83.0) | 21.5 (9.5,36.2) |

Impact of sub-optimal prevalences in low birthweight (LBW), preterm birth (PTB), or small for gestational age (SGA) on human capital and labor market outcomes in Gambia.

^1^ TMRED = Theoretical Minimum Risk Exposure Distribution (TMRED)

^2^ Source: United National Population Division World Population Prospects 2019.

^3^ Country specific annual wage data from World Indicators Database. Average yearly wage was estimated to be 2/3 of the gross domestic product in 2010 constant US dollars and 2011 International dollars, adjusted for purchasing power parity.

**Georgia**

**Super region:** Central Europe, Eastern Europe, Central Asia**; Sub region:** Central Asia

**Number of live births^3^:** 272,000

**Probability of survival to age 25^2^**: 0.98

**GDP per capita 2010 US dollars (estimated annual wage)^3^:**  3,965

**GDP per capita 2011 International dollars (estimated annual wage)^3^:**  9,005

| **Birth outcome** | **Current**  **prevalence (%)** | **TMRED^1^ (%)** | **Total school years gained per birth cohort**  **(in 1000s)** | **Increase in lifetime earnings in USD per child (20 to 59yrs)** | **Benefits by cohorts (20 to 59yrs)** **Lifetime wages**  **(in USD millions)** |
| --- | --- | --- | --- | --- | --- |
| **LBW** | 6.0 (4.6,7.5) | 3.2 | 2.1 (0.5,4.5) | 27.0 (5.3,69.0) | 7.4 (1.4,18.8) |
| **PTB** | 10.0 (9.2,10.9) | 5.5 | 3.8 (0.5,7.5) | 48.7 (7.1,116.2) | 13.3 (1.9,31.6) |
| **SGA** | 15.4 (10.7,21.6) | 10 | 5.5 (0.2,13.1) | 69.8 (1.8,205.0) | 19.0 (0.5,55.8) |

Impact of sub-optimal prevalences in low birthweight (LBW), preterm birth (PTB), or small for gestational age (SGA) on human capital and labor market outcomes in Georgia.

^1^ TMRED = Theoretical Minimum Risk Exposure Distribution (TMRED)

^2^ Source: United National Population Division World Population Prospects 2019.

^3^ Country specific annual wage data from World Indicators Database. Average yearly wage was estimated to be 2/3 of the gross domestic product in 2010 constant US dollars and 2011 International dollars, adjusted for purchasing power parity.

**Ghana**

**Super region:** Sub-Saharan Africa**; Sub region:** Western Sub-Saharan Africa

**Number of live births^3^:** 4,356,000

**Probability of survival to age 25^2^**: 0.89

**GDP per capita 2010 US dollars (estimated annual wage)^3^:**  1,625

**GDP per capita 2011 International dollars (estimated annual wage)^3^:**  3,787

| **Birth outcome** | **Current**  **prevalence (%)** | **TMRED^1^ (%)** | **Total school years gained per birth cohort**  **(in 1000s)** | **Increase in lifetime earnings in USD per child (20 to 59yrs)** | **Benefits by cohorts (20 to 59yrs)** **Lifetime wages**  **(in USD millions)** |
| --- | --- | --- | --- | --- | --- |
| **LBW** | 14.2 (11.3,18.5) | 3.2 | 118.5 (33.4,225.6) | 42.4 (10.9,86.1) | 184.6 (47.5,375.1) |
| **PTB** | 12.0 (8.6,16.7) | 5.5 | 74.7 (9.5,184.1) | 26.1 (3.6,68.7) | 113.6 (15.7,299.4) |
| **SGA** | 24.2 (20.1,28.3) | 10 | 221.9 (95.5,371.9) | 80.2 (34.2,143.0) | 349.1 (148.8,622.8) |

Impact of sub-optimal prevalences in low birthweight (LBW), preterm birth (PTB), or small for gestational age (SGA) on human capital and labor market outcomes in Ghana.

^1^ TMRED = Theoretical Minimum Risk Exposure Distribution (TMRED)

^2^ Source: United National Population Division World Population Prospects 2019.

^3^ Country specific annual wage data from World Indicators Database. Average yearly wage was estimated to be 2/3 of the gross domestic product in 2010 constant US dollars and 2011 International dollars, adjusted for purchasing power parity.

**Grenada**

**Super region:** Latin America and Caribbean**; Sub region:** Caribbean

**Number of live births^3^:** 9,000

**Probability of survival to age 25^2^**: 0.96

**GDP per capita 2010 US dollars (estimated annual wage)^3^:**  8,194

**GDP per capita 2011 International dollars (estimated annual wage)^3^:**  12,436

| **Birth outcome** | **Current**  **prevalence (%)** | **TMRED^1^ (%)** | **Total school years gained per birth cohort**  **(in 1000s)** | **Increase in lifetime earnings in USD per child (20 to 59yrs)** | **Benefits by cohorts (20 to 59yrs)** **Lifetime wages**  **(in USD millions)** |
| --- | --- | --- | --- | --- | --- |
| **LBW** | 7.5 (3.8,11.2) | 3.2 | 0.1 (0.0,0.3) | 162.1 (14.5,405.3) | 1.5 (0.1,3.6) |
| **PTB** | 9.8 (8.6,16.7) | 5.5 | 0.1 (0.0,0.3) | 175.1 (0.2,496.2) | 1.6 (0.0,4.5) |
| **SGA** | 12.1 (8.8,16.5) | 10 | 0.1 (0.0,0.2) | 111.3 (0.0,390.9) | 1.0 (0.0,3.5) |

Impact of sub-optimal prevalences in low birthweight (LBW), preterm birth (PTB), or small for gestational age (SGA) on human capital and labor market outcomes in Grenada.

^1^ TMRED = Theoretical Minimum Risk Exposure Distribution (TMRED)

^2^ Source: United National Population Division World Population Prospects 2019.

^3^ Country specific annual wage data from World Indicators Database. Average yearly wage was estimated to be 2/3 of the gross domestic product in 2010 constant US dollars and 2011 International dollars, adjusted for purchasing power parity.

**Guatemala**

**Super region:** Latin America and Caribbean**; Sub region:** Central Latin America

**Number of live births^3^:** 2,115,000

**Probability of survival to age 25^2^**: 0.94

**GDP per capita 2010 US dollars (estimated annual wage)^3^:**  3,069

**GDP per capita 2011 International dollars (estimated annual wage)^3^:**  7,293

| **Birth outcome** | **Current**  **prevalence (%)** | **TMRED^1^ (%)** | **Total school years gained per birth cohort**  **(in 1000s)** | **Increase in lifetime earnings in USD per child (20 to 59yrs)** | **Benefits by cohorts (20 to 59yrs)** **Lifetime wages**  **(in USD millions)** |
| --- | --- | --- | --- | --- | --- |
| **LBW** | 11.0 (8.3,14.0) | 3.2 | 43.2 (12.4,85.2) | 104.1 (26.6,218.2) | 220.1 (56.3,461.5) |
| **PTB** | 9.8 (8.6,11.3) | 5.5 | 26.0 (3.5,52.1) | 62.6 (8.1,132.4) | 132.5 (17.2,280.1) |
| **SGA** | 13.6 (10.4,17.4) | 10 | 28.0 (3.1,69.0) | 68.0 (6.5,177.4) | 143.9 (13.8,375.3) |

Impact of sub-optimal prevalences in low birthweight (LBW), preterm birth (PTB), or small for gestational age (SGA) on human capital and labor market outcomes in Guatemala.

^1^ TMRED = Theoretical Minimum Risk Exposure Distribution (TMRED)

^2^ Source: United National Population Division World Population Prospects 2019.

^3^ Country specific annual wage data from World Indicators Database. Average yearly wage was estimated to be 2/3 of the gross domestic product in 2010 constant US dollars and 2011 International dollars, adjusted for purchasing power parity.

**Guinea**

**Super region:** Sub-Saharan Africa**; Sub region:** Western Sub-Saharan Africa

**Number of live births^3^:** 2,245,000

**Probability of survival to age 25^2^**: 0.86

**GDP per capita 2010 US dollars (estimated annual wage)^3^:**  750

**GDP per capita 2011 International dollars (estimated annual wage)^3^:**  1,860

| **Birth outcome** | **Current**  **prevalence (%)** | **TMRED^1^ (%)** | **Total school years gained per birth cohort**  **(in 1000s)** | **Increase in lifetime earnings in USD per child (20 to 59yrs)** | **Benefits by cohorts (20 to 59yrs)** **Lifetime wages**  **(in USD millions)** |
| --- | --- | --- | --- | --- | --- |
| **LBW** | 12.2 (6.8,17.6) | 3.2 | 49.0 (9.8,98.4) | 17.9 (3.6,40.0) | 40.3 (8.0,89.8) |
| **PTB** | 12.0 (8.6,16.7) | 5.5 | 36.6 (4.8,86.0) | 13.1 (1.8,34.4) | 29.4 (4.2,77.2) |
| **SGA** | 24.7 (21.0,28.7) | 10 | 115.5 (52.6,187.1) | 43.1 (17.0,72.9) | 96.9 (38.2,163.7) |

Impact of sub-optimal prevalences in low birthweight (LBW), preterm birth (PTB), or small for gestational age (SGA) on human capital and labor market outcomes in Guinea.

^1^ TMRED = Theoretical Minimum Risk Exposure Distribution (TMRED)

^2^ Source: United National Population Division World Population Prospects 2019.

^3^ Country specific annual wage data from World Indicators Database. Average yearly wage was estimated to be 2/3 of the gross domestic product in 2010 constant US dollars and 2011 International dollars, adjusted for purchasing power parity.

**Guinea-Bissau**

**Super region:** Sub-Saharan Africa**; Sub region:** Western Sub-Saharan Africa

**Number of live births^3^:** 328,000

**Probability of survival to age 25^2^**: 0.84

**GDP per capita 2010 US dollars (estimated annual wage)^3^:**  574

**GDP per capita 2011 International dollars (estimated annual wage)^3^:**  1,474

| **Birth outcome** | **Current**  **prevalence (%)** | **TMRED^1^ (%)** | **Total school years gained per birth cohort**  **(in 1000s)** | **Increase in lifetime earnings in USD per child (20 to 59yrs)** | **Benefits by cohorts (20 to 59yrs)** **Lifetime wages**  **(in USD millions)** |
| --- | --- | --- | --- | --- | --- |
| **LBW** | 21.1 (16.7,27.4) | 3.2 | 14.0 (3.9,25.5) | 27.1 (7.4,54.4) | 8.9 (2.4,17.8) |
| **PTB** | 12.0 (8.6,16.7) | 5.5 | 5.1 (0.6,13.1) | 9.7 (1.3,26.5) | 3.2 (0.4,8.7) |
| **SGA** | 39.1 (33.5,45.0) | 10 | 32.6 (15.6,50.7) | 63.7 (29.6,105.3) | 20.9 (9.7,34.5) |

Impact of sub-optimal prevalences in low birthweight (LBW), preterm birth (PTB), or small for gestational age (SGA) on human capital and labor market outcomes in Guinea-Bissau.

^1^ TMRED = Theoretical Minimum Risk Exposure Distribution (TMRED)

^2^ Source: United National Population Division World Population Prospects 2019.

^3^ Country specific annual wage data from World Indicators Database. Average yearly wage was estimated to be 2/3 of the gross domestic product in 2010 constant US dollars and 2011 International dollars, adjusted for purchasing power parity.

**Guyana**

**Super region:** Latin America and Caribbean**; Sub region:** Caribbean

**Number of live births^3^:** 78,000

**Probability of survival to age 25^2^**: 0.92

**GDP per capita 2010 US dollars (estimated annual wage)^3^:**  3,690

**GDP per capita 2011 International dollars (estimated annual wage)^3^:**  7,087

| **Birth outcome** | **Current**  **prevalence (%)** | **TMRED^1^ (%)** | **Total school years gained per birth cohort**  **(in 1000s)** | **Increase in lifetime earnings in USD per child (20 to 59yrs)** | **Benefits by cohorts (20 to 59yrs)** **Lifetime wages**  **(in USD millions)** |
| --- | --- | --- | --- | --- | --- |
| **LBW** | 15.6 (12.2,19.6) | 3.2 | 2.5 (0.7,4.6) | 211.5 (55.2,394.9) | 16.5 (4.3,30.8) |
| **PTB** | 9.8 (8.6,11.3) | 5.5 | 0.9 (0.1,1.9) | 78.4 (12.3,165.7) | 6.1 (1.0,12.9) |
| **SGA** | 19.1 (15.1,24.2) | 10 | 2.6 (1.1,4.8) | 219.6 (89.7,410.2) | 17.1 (7.0,32.0) |

Impact of sub-optimal prevalences in low birthweight (LBW), preterm birth (PTB), or small for gestational age (SGA) on human capital and labor market outcomes in Guyana.

^1^ TMRED = Theoretical Minimum Risk Exposure Distribution (TMRED)

^2^ Source: United National Population Division World Population Prospects 2019.

^3^ Country specific annual wage data from World Indicators Database. Average yearly wage was estimated to be 2/3 of the gross domestic product in 2010 constant US dollars and 2011 International dollars, adjusted for purchasing power parity.

**Haiti**

**Super region:** Latin America and Caribbean**; Sub region:** Caribbean

**Number of live births^3^:** 1,355,000

**Probability of survival to age 25^2^**: 0.86

**GDP per capita 2010 US dollars (estimated annual wage)^3^:**  729

**GDP per capita 2011 International dollars (estimated annual wage)^3^:**  1,654

| **Birth outcome** | **Current**  **prevalence (%)** | **TMRED^1^ (%)** | **Total school years gained per birth cohort**  **(in 1000s)** | **Increase in lifetime earnings in USD per child (20 to 59yrs)** | **Benefits by cohorts (20 to 59yrs)** **Lifetime wages**  **(in USD millions)** |
| --- | --- | --- | --- | --- | --- |
| **LBW** | 24.6 (20.9,28.4) | 3.2 | 70.5 (21.5,123.7) | 66.6 (19.7,119.0) | 90.2 (26.7,161.2) |
| **PTB** | 9.8 (8.6,11.3) | 5.5 | 15.2 (2.3,30.6) | 14.6 (2.1,29.4) | 19.7 (2.9,39.9) |
| **SGA** | 34.8 (28.2,42.3) | 10 | 115.9 (53.3,198.3) | 109.1 (50.4,191.2) | 147.8 (68.2,259.1) |

Impact of sub-optimal prevalences in low birthweight (LBW), preterm birth (PTB), or small for gestational age (SGA) on human capital and labor market outcomes in Haiti.

^1^ TMRED = Theoretical Minimum Risk Exposure Distribution (TMRED)

^2^ Source: United National Population Division World Population Prospects 2019.

^3^ Country specific annual wage data from World Indicators Database. Average yearly wage was estimated to be 2/3 of the gross domestic product in 2010 constant US dollars and 2011 International dollars, adjusted for purchasing power parity.

**Honduras**

**Super region:** Latin America and Caribbean**; Sub region:** Central Latin America

**Number of live births^3^:** 1,037,000

**Probability of survival to age 25^2^**: 0.95

**GDP per capita 2010 US dollars (estimated annual wage)^3^:**  2,053

**GDP per capita 2011 International dollars (estimated annual wage)^3^:**  4,247

| **Birth outcome** | **Current**  **prevalence (%)** | **TMRED^1^ (%)** | **Total school years gained per birth cohort**  **(in 1000s)** | **Increase in lifetime earnings in USD per child (20 to 59yrs)** | **Benefits by cohorts (20 to 59yrs)** **Lifetime wages**  **(in USD millions)** |
| --- | --- | --- | --- | --- | --- |
| **LBW** | 10.9 (8.6,13.8) | 3.2 | 21.5 (6.4,39.6) | 76.3 (14.5,194.1) | 79.1 (15.0,201.3) |
| **PTB** | 9.8 (8.6,11.3) | 5.5 | 13.0 (1.9,26.2) | 47.8 (5.5,123.0) | 49.5 (5.7,127.5) |
| **SGA** | 16.2 (12.3,21.6) | 10 | 23.7 (5.7,51.2) | 88.2 (14.3,254.5) | 91.5 (14.8,263.9) |

Impact of sub-optimal prevalences in low birthweight (LBW), preterm birth (PTB), or small for gestational age (SGA) on human capital and labor market outcomes in Honduras.

^1^ TMRED = Theoretical Minimum Risk Exposure Distribution (TMRED)

^2^ Source: United National Population Division World Population Prospects 2019.

^3^ Country specific annual wage data from World Indicators Database. Average yearly wage was estimated to be 2/3 of the gross domestic product in 2010 constant US dollars and 2011 International dollars, adjusted for purchasing power parity.

**India**

**Super region:** South Asia**; Sub region:** South Asia

**Number of live births^3^:** 121,189,000

**Probability of survival to age 25^2^**: 0.94

**GDP per capita 2010 US dollars (estimated annual wage)^3^:**  1,752

**GDP per capita 2011 International dollars (estimated annual wage)^3^:**  5,743

| **Birth outcome** | **Current**  **prevalence (%)** | **TMRED^1^ (%)** | **Total school years gained per birth cohort**  **(in 1000s)** | **Increase in lifetime earnings in USD per child (20 to 59yrs)** | **Benefits by cohorts (20 to 59yrs)** **Lifetime wages**  **(in USD millions)** |
| --- | --- | --- | --- | --- | --- |
| **LBW** | 27.6 (18.1,37.0) | 3.2 | 7817.5 (2063.6,15025.7) | 163.1 (43.0,313.4) | 19769.6 (5215.5,37975.1) |
| **PTB** | 13.6 (11.1,16.1) | 5.5 | 2801.7 (417.2,5655.8) | 58.5 (8.6,119.0) | 7083.9 (1041.3,14426.9) |
| **SGA** | 46.9 (42.6,53.0) | 10 | 17010.8 (8307.5,26585.9) | 355.8 (169.5,557.4) | 43113.6 (20544.5,67549.8) |

Impact of sub-optimal prevalences in low birthweight (LBW), preterm birth (PTB), or small for gestational age (SGA) on human capital and labor market outcomes in India.

^1^ TMRED = Theoretical Minimum Risk Exposure Distribution (TMRED)

^2^ Source: United National Population Division World Population Prospects 2019.

^3^ Country specific annual wage data from World Indicators Database. Average yearly wage was estimated to be 2/3 of the gross domestic product in 2010 constant US dollars and 2011 International dollars, adjusted for purchasing power parity.

**Indonesia**

**Super region:** Southeast Asia, East Asia, and Oceania**; Sub region:** Southeast Asia

**Number of live births^3^:** 24,208,000

**Probability of survival to age 25^2^**: 0.95

**GDP per capita 2010 US dollars (estimated annual wage)^3^:**  3,824

**GDP per capita 2011 International dollars (estimated annual wage)^3^:**  10,359

| **Birth outcome** | **Current**  **prevalence (%)** | **TMRED^1^ (%)** | **Total school years gained per birth cohort**  **(in 1000s)** | **Increase in lifetime earnings in USD per child (20 to 59yrs)** | **Benefits by cohorts (20 to 59yrs)** **Lifetime wages**  **(in USD millions)** |
| --- | --- | --- | --- | --- | --- |
| **LBW** | 10.0 (7.4,12.7) | 3.2 | 444.7 (123.5,841.7) | 96.7 (19.0,233.7) | 2339.9 (458.9,5657.1) |
| **PTB** | 10.4 (8.7,11.9) | 5.5 | 352.7 (52.1,722.7) | 75.2 (8.4,200.3) | 1819.8 (204.4,4848.7) |
| **SGA** | 23.8 (18.5,29.8) | 10 | 1278.9 (508.8,2265.7) | 284.1 (78.3,623.0) | 6877.0 (1895.5,15081.2) |

Impact of sub-optimal prevalences in low birthweight (LBW), preterm birth (PTB), or small for gestational age (SGA) on human capital and labor market outcomes in Indonesia.

^1^ TMRED = Theoretical Minimum Risk Exposure Distribution (TMRED)

^2^ Source: United National Population Division World Population Prospects 2019.

^3^ Country specific annual wage data from World Indicators Database. Average yearly wage was estimated to be 2/3 of the gross domestic product in 2010 constant US dollars and 2011 International dollars, adjusted for purchasing power parity.

**Iran**

**Super region:** North Africa and Middle East**; Sub region:** North Africa and Middle East

**Number of live births^3^:** 7,758,000

**Probability of survival to age 25^2^**: 0.97

**GDP per capita 2010 US dollars (estimated annual wage)^3^:**  6,073

**GDP per capita 2011 International dollars (estimated annual wage)^3^:**  16,683

| **Birth outcome** | **Current**  **prevalence (%)** | **TMRED^1^ (%)** | **Total school years gained per birth cohort**  **(in 1000s)** | **Increase in lifetime earnings in USD per child (20 to 59yrs)** | **Benefits by cohorts (20 to 59yrs)** **Lifetime wages**  **(in USD millions)** |
| --- | --- | --- | --- | --- | --- |
| **LBW** | 7.2 (3.2,11.2) | 3.2 | 79.6 (0.0,215.3) | 87.4 (0.0,242.1) | 678.0 (0.0,1878.3) |
| **PTB** | 6.0 (4.0,8.7) | 5.5 | 7.1 (0.0,72.0) | 7.7 (0.0,82.1) | 59.8 (0.0,637.2) |
| **SGA** | 16.9 (12.0,22.3) | 10 | 204.1 (45.9,433.6) | 222.1 (50.0,482.3) | 1723.3 (387.9,3741.7) |

Impact of sub-optimal prevalences in low birthweight (LBW), preterm birth (PTB), or small for gestational age (SGA) on human capital and labor market outcomes in Iran.

^1^ TMRED = Theoretical Minimum Risk Exposure Distribution (TMRED)

^2^ Source: United National Population Division World Population Prospects 2019.

^3^ Country specific annual wage data from World Indicators Database. Average yearly wage was estimated to be 2/3 of the gross domestic product in 2010 constant US dollars and 2011 International dollars, adjusted for purchasing power parity.

**Iraq**

**Super region:** North Africa and Middle East**; Sub region:** North Africa and Middle East

**Number of live births^3^:** 5,520,000

**Probability of survival to age 25^2^**: 0.95

**GDP per capita 2010 US dollars (estimated annual wage)^3^:**  5,298

**GDP per capita 2011 International dollars (estimated annual wage)^3^:**  14,964

| **Birth outcome** | **Current**  **prevalence (%)** | **TMRED^1^ (%)** | **Total school years gained per birth cohort**  **(in 1000s)** | **Increase in lifetime earnings in USD per child (20 to 59yrs)** | **Benefits by cohorts (20 to 59yrs)** **Lifetime wages**  **(in USD millions)** |
| --- | --- | --- | --- | --- | --- |
| **LBW** | 5.6 (1.6,9.6) | 3.2 | 34.0 (0.0,111.9) | 3.7 (0.0,16.0) | 20.5 (0.0,88.4) |
| **PTB** | 10.4 (8.7,11.9) | 5.5 | 79.1 (11.7,154.8) | 8.8 (0.8,22.1) | 48.3 (4.3,122.2) |
| **SGA** | 13.5 (9.3,20.3) | 10 | 70.1 (0.0,217.6) | 7.5 (0.0,30.3) | 41.6 (0.0,167.4) |

Impact of sub-optimal prevalences in low birthweight (LBW), preterm birth (PTB), or small for gestational age (SGA) on human capital and labor market outcomes in Iraq.

^1^ TMRED = Theoretical Minimum Risk Exposure Distribution (TMRED)

^2^ Source: United National Population Division World Population Prospects 2019.

^3^ Country specific annual wage data from World Indicators Database. Average yearly wage was estimated to be 2/3 of the gross domestic product in 2010 constant US dollars and 2011 International dollars, adjusted for purchasing power parity.

**Jamaica**

**Super region:** Latin America and Caribbean**; Sub region:** Caribbean

**Number of live births^3^:** 237,000

**Probability of survival to age 25^2^**: 0.97

**GDP per capita 2010 US dollars (estimated annual wage)^3^:**  4,714

**GDP per capita 2011 International dollars (estimated annual wage)^3^:**  8,047

| **Birth outcome** | **Current**  **prevalence (%)** | **TMRED^1^ (%)** | **Total school years gained per birth cohort**  **(in 1000s)** | **Increase in lifetime earnings in USD per child (20 to 59yrs)** | **Benefits by cohorts (20 to 59yrs)** **Lifetime wages**  **(in USD millions)** |
| --- | --- | --- | --- | --- | --- |
| **LBW** | 14.6 (11.4,18.9) | 3.2 | 7.5 (2.2,13.7) | 259.2 (76.0,503.2) | 61.4 (18.0,119.3) |
| **PTB** | 9.8 (8.6,11.3) | 5.5 | 3.0 (0.5,6.0) | 104.8 (17.9,221.5) | 24.8 (4.2,52.5) |
| **SGA** | 18.1 (14.0,23.4) | 10 | 7.4 (2.4,14.7) | 255.6 (81.6,531.9) | 60.6 (19.3,126.0) |

Impact of sub-optimal prevalences in low birthweight (LBW), preterm birth (PTB), or small for gestational age (SGA) on human capital and labor market outcomes in Jamaica.

^1^ TMRED = Theoretical Minimum Risk Exposure Distribution (TMRED)

^2^ Source: United National Population Division World Population Prospects 2019.

^3^ Country specific annual wage data from World Indicators Database. Average yearly wage was estimated to be 2/3 of the gross domestic product in 2010 constant US dollars and 2011 International dollars, adjusted for purchasing power parity.

**Jordan**

**Super region:** North Africa and Middle East**; Sub region:** North Africa and Middle East

**Number of live births^3^:** 1,073,000

**Probability of survival to age 25^2^**: 0.97

**GDP per capita 2010 US dollars (estimated annual wage)^3^:**  3,275

**GDP per capita 2011 International dollars (estimated annual wage)^3^:**  8,408

| **Birth outcome** | **Current**  **prevalence (%)** | **TMRED^1^ (%)** | **Total school years gained per birth cohort**  **(in 1000s)** | **Increase in lifetime earnings in USD per child (20 to 59yrs)** | **Benefits by cohorts (20 to 59yrs)** **Lifetime wages**  **(in USD millions)** |
| --- | --- | --- | --- | --- | --- |
| **LBW** | 13.8 (11.0,18.0) | 3.2 | 31.4 (8.6,57.1) | 112.1 (32.4,234.1) | 120.3 (34.8,251.1) |
| **PTB** | 17.8 (12.6,23.9) | 5.5 | 38.5 (5.4,85.7) | 141.1 (19.0,349.3) | 151.4 (20.3,374.8) |
| **SGA** | 18.9 (13.4,25.2) | 10 | 35.6 (11.2,74.7) | 130.7 (37.3,311.3) | 140.3 (40.0,334.1) |

Impact of sub-optimal prevalences in low birthweight (LBW), preterm birth (PTB), or small for gestational age (SGA) on human capital and labor market outcomes in Jordan.

^1^ TMRED = Theoretical Minimum Risk Exposure Distribution (TMRED)

^2^ Source: United National Population Division World Population Prospects 2019.

^3^ Country specific annual wage data from World Indicators Database. Average yearly wage was estimated to be 2/3 of the gross domestic product in 2010 constant US dollars and 2011 International dollars, adjusted for purchasing power parity.

**Kazakhstan**

**Super region:** Central Europe, Eastern Europe, Central Asia**; Sub region:** Central Asia

**Number of live births^3^:** 1,943,000

**Probability of survival to age 25^2^**: 0.97

**GDP per capita 2010 US dollars (estimated annual wage)^3^:**  10,617

**GDP per capita 2011 International dollars (estimated annual wage)^3^:**  23,524

| **Birth outcome** | **Current**  **prevalence (%)** | **TMRED^1^ (%)** | **Total school years gained per birth cohort**  **(in 1000s)** | **Increase in lifetime earnings in USD per child (20 to 59yrs)** | **Benefits by cohorts (20 to 59yrs)** **Lifetime wages**  **(in USD millions)** |
| --- | --- | --- | --- | --- | --- |
| **LBW** | 5.4 (5.0,5.9) | 3.2 | 12.0 (3.7,21.6) | 94.0 (28.0,171.0) | 182.6 (54.3,332.2) |
| **PTB** | 5.2 (2.8,8.8) | 5.5 | 0.0 (0.0,19.1) | 0.0 (0.0,152.1) | 0.0 (0.0,295.5) |
| **SGA** | 16.6 (11.8,22.8) | 10 | 49.7 (7.3,107.5) | 395.3 (56.7,846.8) | 768.1 (110.1,1645.3) |

Impact of sub-optimal prevalences in low birthweight (LBW), preterm birth (PTB), or small for gestational age (SGA) on human capital and labor market outcomes in Kazakhstan.

^1^ TMRED = Theoretical Minimum Risk Exposure Distribution (TMRED)

^2^ Source: United National Population Division World Population Prospects 2019.

^3^ Country specific annual wage data from World Indicators Database. Average yearly wage was estimated to be 2/3 of the gross domestic product in 2010 constant US dollars and 2011 International dollars, adjusted for purchasing power parity.

**Kenya**

**Super region:** Sub-Saharan Africa**; Sub region:** Eastern Sub-Saharan Africa

**Number of live births^3^:** 7,345,000

**Probability of survival to age 25^2^**: 0.92

**GDP per capita 2010 US dollars (estimated annual wage)^3^:**  1,093

**GDP per capita 2011 International dollars (estimated annual wage)^3^:**  2,798

| **Birth outcome** | **Current**  **prevalence (%)** | **TMRED^1^ (%)** | **Total school years gained per birth cohort**  **(in 1000s)** | **Increase in lifetime earnings in USD per child (20 to 59yrs)** | **Benefits by cohorts (20 to 59yrs)** **Lifetime wages**  **(in USD millions)** |
| --- | --- | --- | --- | --- | --- |
| **LBW** | 11.5 (8.9,14.5) | 3.2 | 156.9 (46.9,290.6) | 47.3 (14.4,87.2) | 347.7 (105.9,640.6) |
| **PTB** | 8.6 (6.3,11.3) | 5.5 | 59.8 (2.4,163.8) | 18.1 (0.8,50.0) | 132.7 (5.5,367.3) |
| **SGA** | 15.5 (12.7,19.5) | 10 | 146.7 (44.8,304.1) | 44.0 (14.4,91.1) | 323.4 (105.7,669.2) |

Impact of sub-optimal prevalences in low birthweight (LBW), preterm birth (PTB), or small for gestational age (SGA) on human capital and labor market outcomes in Kenya.

^1^ TMRED = Theoretical Minimum Risk Exposure Distribution (TMRED)

^2^ Source: United National Population Division World Population Prospects 2019.

^3^ Country specific annual wage data from World Indicators Database. Average yearly wage was estimated to be 2/3 of the gross domestic product in 2010 constant US dollars and 2011 International dollars, adjusted for purchasing power parity.

**Kiribati**

**Super region:** Southeast Asia, East Asia, and Oceania**; Sub region:** Oceania

**Number of live births^3^:** 16,000

**Probability of survival to age 25^2^**: 0.91

**GDP per capita 2010 US dollars (estimated annual wage)^3^:**  1,711

**GDP per capita 2011 International dollars (estimated annual wage)^3^:**  1,976

| **Birth outcome** | **Current**  **prevalence (%)** | **TMRED^1^ (%)** | **Total school years gained per birth cohort**  **(in 1000s)** | **Increase in lifetime earnings in USD per child (20 to 59yrs)** | **Benefits by cohorts (20 to 59yrs)** **Lifetime wages**  **(in USD millions)** |
| --- | --- | --- | --- | --- | --- |
| **LBW** | 8.2 (5.5,10.8) | 3.2 | 0.2 (0.1,0.4) | 22.1 (4.9,57.0) | 0.4 (0.1,0.9) |
| **PTB** | 10.0 (7.9,12.7) | 5.5 | 0.2 (0.0,0.4) | 22.6 (2.4,58.0) | 0.4 (0.0,0.9) |
| **SGA** | 18.7 (13.7,25.3) | 10 | 0.5 (0.1,1.0) | 56.3 (14.4,133.2) | 0.9 (0.2,2.1) |

Impact of sub-optimal prevalences in low birthweight (LBW), preterm birth (PTB), or small for gestational age (SGA) on human capital and labor market outcomes in Kiribati.

^1^ TMRED = Theoretical Minimum Risk Exposure Distribution (TMRED)

^2^ Source: United National Population Division World Population Prospects 2019.

^3^ Country specific annual wage data from World Indicators Database. Average yearly wage was estimated to be 2/3 of the gross domestic product in 2010 constant US dollars and 2011 International dollars, adjusted for purchasing power parity.

**Kyrgyzstan**

**Super region:** Central Europe, Eastern Europe, Central Asia**; Sub region:** Central Asia

**Number of live births^3^:** 774,000

**Probability of survival to age 25^2^**: 0.97

**GDP per capita 2010 US dollars (estimated annual wage)^3^:**  1,020

**GDP per capita 2011 International dollars (estimated annual wage)^3^:**  3,234

| **Birth outcome** | **Current**  **prevalence (%)** | **TMRED^1^ (%)** | **Total school years gained per birth cohort**  **(in 1000s)** | **Increase in lifetime earnings in USD per child (20 to 59yrs)** | **Benefits by cohorts (20 to 59yrs)** **Lifetime wages**  **(in USD millions)** |
| --- | --- | --- | --- | --- | --- |
| **LBW** | 5.5 (5.2,5.8) | 3.2 | 5.0 (1.5,8.5) | 5.7 (1.6,12.0) | 4.4 (1.2,9.3) |
| **PTB** | 10.4 (8.7,11.9) | 5.5 | 11.3 (1.7,23.6) | 13.1 (1.7,32.4) | 10.2 (1.3,25.1) |
| **SGA** | 14.9 (10.0,21.5) | 10 | 14.2 (0.0,35.3) | 16.4 (0.0,46.2) | 12.7 (0.0,35.7) |

Impact of sub-optimal prevalences in low birthweight (LBW), preterm birth (PTB), or small for gestational age (SGA) on human capital and labor market outcomes in Kyrgyzstan.

^1^ TMRED = Theoretical Minimum Risk Exposure Distribution (TMRED)

^2^ Source: United National Population Division World Population Prospects 2019.

^3^ Country specific annual wage data from World Indicators Database. Average yearly wage was estimated to be 2/3 of the gross domestic product in 2010 constant US dollars and 2011 International dollars, adjusted for purchasing power parity.

**Lao People's Democratic Republic**

**Super region:** Southeast Asia, East Asia, and Oceania**; Sub region:** Southeast Asia

**Number of live births^3^:** 835,000

**Probability of survival to age 25^2^**: 0.92

**GDP per capita 2010 US dollars (estimated annual wage)^3^:**  1,539

**GDP per capita 2011 International dollars (estimated annual wage)^3^:**  5,689

| **Birth outcome** | **Current**  **prevalence (%)** | **TMRED^1^ (%)** | **Total school years gained per birth cohort**  **(in 1000s)** | **Increase in lifetime earnings in USD per child (20 to 59yrs)** | **Benefits by cohorts (20 to 59yrs)** **Lifetime wages**  **(in USD millions)** |
| --- | --- | --- | --- | --- | --- |
| **LBW** | 17.3 (7.3,11.7) | 3.2 | 31.2 (9.0,55.6) | 64.0 (11.8,149.5) | 53.4 (9.9,124.8) |
| **PTB** | 10.4 (8.7,11.9) | 5.5 | 11.7 (1.7,23.6) | 24.1 (1.9,62.7) | 20.2 (1.6,52.4) |
| **SGA** | 22.8 (17.5,29.5) | 10 | 38.9 (16.3,72.6) | 82.2 (18.4,197.1) | 68.6 (15.4,164.6) |

Impact of sub-optimal prevalences in low birthweight (LBW), preterm birth (PTB), or small for gestational age (SGA) on human capital and labor market outcomes in Lao People's Democratic Republic.

^1^ TMRED = Theoretical Minimum Risk Exposure Distribution (TMRED)

^2^ Source: United National Population Division World Population Prospects 2019.

^3^ Country specific annual wage data from World Indicators Database. Average yearly wage was estimated to be 2/3 of the gross domestic product in 2010 constant US dollars and 2011 International dollars, adjusted for purchasing power parity.

**Lebanon**

**Super region:** North Africa and Middle East**; Sub region:** North Africa and Middle East

**Number of live births^3^:** 587,000

**Probability of survival to age 25^2^**: 0.98

**GDP per capita 2010 US dollars (estimated annual wage)^3^:**  6,401

**GDP per capita 2011 International dollars (estimated annual wage)^3^:**  11,887

| **Birth outcome** | **Current**  **prevalence (%)** | **TMRED^1^ (%)** | **Total school years gained per birth cohort**  **(in 1000s)** | **Increase in lifetime earnings in USD per child (20 to 59yrs)** | **Benefits by cohorts (20 to 59yrs)** **Lifetime wages**  **(in USD millions)** |
| --- | --- | --- | --- | --- | --- |
| **LBW** | 9.2 (7.3,11.7) | 3.2 | 9.8 (2.9,18.9) | 127.0 (36.2,274.3) | 74.6 (21.2,161.0) |
| **PTB** | 9.0 (5.3,14.3) | 5.5 | 5.7 (0.0,19.1) | 73.4 (0.0,272.3) | 43.1 (0.0,159.8) |
| **SGA** | 12.6 (8.4,18.6) | 10 | 5.6 (0.0,19.6) | 71.5 (0.0,269.0) | 41.9 (0.0,157.9) |

Impact of sub-optimal prevalences in low birthweight (LBW), preterm birth (PTB), or small for gestational age (SGA) on human capital and labor market outcomes in Lebanon.

^1^ TMRED = Theoretical Minimum Risk Exposure Distribution (TMRED)

^2^ Source: United National Population Division World Population Prospects 2019.

^3^ Country specific annual wage data from World Indicators Database. Average yearly wage was estimated to be 2/3 of the gross domestic product in 2010 constant US dollars and 2011 International dollars, adjusted for purchasing power parity.

**Lesotho**

**Super region:** Sub-Saharan Africa**; Sub region:** Southern Sub-Saharan Africa

**Number of live births^3^:** 284,000

**Probability of survival to age 25^2^**: 0.84

**GDP per capita 2010 US dollars (estimated annual wage)^3^:**  1,403

**GDP per capita 2011 International dollars (estimated annual wage)^3^:**  2,867

| **Birth outcome** | **Current**  **prevalence (%)** | **TMRED^1^ (%)** | **Total school years gained per birth cohort**  **(in 1000s)** | **Increase in lifetime earnings in USD per child (20 to 59yrs)** | **Benefits by cohorts (20 to 59yrs)** **Lifetime wages**  **(in USD millions)** |
| --- | --- | --- | --- | --- | --- |
| **LBW** | 14.6 (11.5,19.3) | 3.2 | 7.9 (2.2,14.1) | 110.8 (30.9,199.6) | 31.5 (8.8,56.7) |
| **PTB** | 12.0 (8.6,16.7) | 5.5 | 4.5 (0.6,10.7) | 64.1 (8.3,150.2) | 18.2 (2.4,42.6) |
| **SGA** | 21.7 (18.2,25.5) | 10 | 11.0 (5.0,18.8) | 156.0 (69.4,266.1) | 44.3 (19.7,75.6) |

Impact of sub-optimal prevalences in low birthweight (LBW), preterm birth (PTB), or small for gestational age (SGA) on human capital and labor market outcomes in Lesotho.

^1^ TMRED = Theoretical Minimum Risk Exposure Distribution (TMRED)

^2^ Source: United National Population Division World Population Prospects 2019.

^3^ Country specific annual wage data from World Indicators Database. Average yearly wage was estimated to be 2/3 of the gross domestic product in 2010 constant US dollars and 2011 International dollars, adjusted for purchasing power parity.

**Liberia**

**Super region:** Sub-Saharan Africa**; Sub region:** Western Sub-Saharan Africa

**Number of live births^3^:** 791,000

**Probability of survival to age 25^2^**: 0.88

**GDP per capita 2010 US dollars (estimated annual wage)^3^:**  571

**GDP per capita 2011 International dollars (estimated annual wage)^3^:**  1,226

| **Birth outcome** | **Current**  **prevalence (%)** | **TMRED^1^ (%)** | **Total school years gained per birth cohort**  **(in 1000s)** | **Increase in lifetime earnings in USD per child (20 to 59yrs)** | **Benefits by cohorts (20 to 59yrs)** **Lifetime wages**  **(in USD millions)** |
| --- | --- | --- | --- | --- | --- |
| **LBW** | 13.7 (8.3,19.1) | 3.2 | 20.1 (5.7,40.2) | 16.3 (4.2,34.9) | 12.9 (3.3,27.6) |
| **PTB** | 12.0 (8.6,16.7) | 5.5 | 13.6 (1.6,31.7) | 11.0 (1.3,26.7) | 8.7 (1.0,21.1) |
| **SGA** | 24.2 (19.9,28.6) | 10 | 39.9 (17.1,67.5) | 32.0 (13.1,58.1) | 25.3 (10.3,45.9) |

Impact of sub-optimal prevalences in low birthweight (LBW), preterm birth (PTB), or small for gestational age (SGA) on human capital and labor market outcomes in Liberia.

^1^ TMRED = Theoretical Minimum Risk Exposure Distribution (TMRED)

^2^ Source: United National Population Division World Population Prospects 2019.

^3^ Country specific annual wage data from World Indicators Database. Average yearly wage was estimated to be 2/3 of the gross domestic product in 2010 constant US dollars and 2011 International dollars, adjusted for purchasing power parity.

**Libya**

**Super region:** North Africa and Middle East**; Sub region:** North Africa and Middle East

**Number of live births^3^:** 631,000

**Probability of survival to age 25^2^**: 0.97

**GDP per capita 2010 US dollars (estimated annual wage)^3^:**  5,900

**GDP per capita 2011 International dollars (estimated annual wage)^3^:**  14,423

| **Birth outcome** | **Current**  **prevalence (%)** | **TMRED^1^ (%)** | **Total school years gained per birth cohort**  **(in 1000s)** | **Increase in lifetime earnings in USD per child (20 to 59yrs)** | **Benefits by cohorts (20 to 59yrs)** **Lifetime wages**  **(in USD millions)** |
| --- | --- | --- | --- | --- | --- |
| **LBW** | 4.0 (0.0,8.0) | 3.2 | 0.9 (0.0,9.0) | 10.5 (0.0,103.9) | 6.6 (0.0,65.5) |
| **PTB** | 10.3 (9.1,11.6) | 5.5 | 9.1 (1.2,17.4) | 100.2 (13.0,221.1) | 63.2 (8.2,139.5) |
| **SGA** | 7.5 (5.0,11.2) | 10 | 0.0 (0.0,1.3) | 0.0 (0.0,14.6) | 0.0 (0.0,9.2) |

Impact of sub-optimal prevalences in low birthweight (LBW), preterm birth (PTB), or small for gestational age (SGA) on human capital and labor market outcomes in Libya.

^1^ TMRED = Theoretical Minimum Risk Exposure Distribution (TMRED)

^2^ Source: United National Population Division World Population Prospects 2019.

^3^ Country specific annual wage data from World Indicators Database. Average yearly wage was estimated to be 2/3 of the gross domestic product in 2010 constant US dollars and 2011 International dollars, adjusted for purchasing power parity.

**Madagascar**

**Super region:** Sub-Saharan Africa**; Sub region:** Eastern Sub-Saharan Africa

**Number of live births^3^:** 4,260,000

**Probability of survival to age 25^2^**: 0.91

**GDP per capita 2010 US dollars (estimated annual wage)^3^:**  410

**GDP per capita 2011 International dollars (estimated annual wage)^3^:**  1,377

| **Birth outcome** | **Current**  **prevalence (%)** | **TMRED^1^ (%)** | **Total school years gained per birth cohort**  **(in 1000s)** | **Increase in lifetime earnings in USD per child (20 to 59yrs)** | **Benefits by cohorts (20 to 59yrs)** **Lifetime wages**  **(in USD millions)** |
| --- | --- | --- | --- | --- | --- |
| **LBW** | 17.1 (11.7,22.9) | 3.2 | 154.0 (39.4,293.5) | 29.9 (7.5,58.6) | 127.4 (32.1,249.5) |
| **PTB** | 12.0 (8.6,16.7) | 5.5 | 75.0 (10.3,168.3) | 14.7 (1.9,33.0) | 62.6 (8.2,140.6) |
| **SGA** | 25.5 (20.2,30.0) | 10 | 239.7 (104.8,416.0) | 46.5 (20.4,84.2) | 198.2 (86.8,358.9) |

Impact of sub-optimal prevalences in low birthweight (LBW), preterm birth (PTB), or small for gestational age (SGA) on human capital and labor market outcomes in Madagascar.

^1^ TMRED = Theoretical Minimum Risk Exposure Distribution (TMRED)

^2^ Source: United National Population Division World Population Prospects 2019.

^3^ Country specific annual wage data from World Indicators Database. Average yearly wage was estimated to be 2/3 of the gross domestic product in 2010 constant US dollars and 2011 International dollars, adjusted for purchasing power parity.

**Malawi**

**Super region:** Sub-Saharan Africa**; Sub region:** Eastern Sub-Saharan Africa

**Number of live births^3^:** 3,072,000

**Probability of survival to age 25^2^**: 0.90

**GDP per capita 2010 US dollars (estimated annual wage)^3^:**  508

**GDP per capita 2011 International dollars (estimated annual wage)^3^:**  1,143

| **Birth outcome** | **Current**  **prevalence (%)** | **TMRED^1^ (%)** | **Total school years gained per birth cohort**  **(in 1000s)** | **Increase in lifetime earnings in USD per child (20 to 59yrs)** | **Benefits by cohorts (20 to 59yrs)** **Lifetime wages**  **(in USD millions)** |
| --- | --- | --- | --- | --- | --- |
| **LBW** | 14.5 (11.3,18.5) | 3.2 | 86.9 (25.9,162.2) | 31.2 (9.1,59.6) | 95.7 (28.1,183.1) |
| **PTB** | 10.5 (7.4,14.3) | 5.5 | 40.9 (4.6,97.8) | 14.7 (1.6,36.6) | 45.3 (5.0,112.3) |
| **SGA** | 23.2 (19.1,27.0) | 10 | 147.3 (67.1,243.3) | 53.0 (23.5,90.1) | 162.7 (72.2,276.8) |

Impact of sub-optimal prevalences in low birthweight (LBW), preterm birth (PTB), or small for gestational age (SGA) on human capital and labor market outcomes in Malawi.

^1^ TMRED = Theoretical Minimum Risk Exposure Distribution (TMRED)

^2^ Source: United National Population Division World Population Prospects 2019.

^3^ Country specific annual wage data from World Indicators Database. Average yearly wage was estimated to be 2/3 of the gross domestic product in 2010 constant US dollars and 2011 International dollars, adjusted for purchasing power parity.

**Malaysia**

**Super region:** Southeast Asia, East Asia, and Oceania**; Sub region:** Southeast Asia

**Number of live births^3^:** 2,636,000

**Probability of survival to age 25^2^**: 0.98

**GDP per capita 2010 US dollars (estimated annual wage)^3^:**  10,912

**GDP per capita 2011 International dollars (estimated annual wage)^3^:**  25,390

| **Birth outcome** | **Current**  **prevalence (%)** | **TMRED^1^ (%)** | **Total school years gained per birth cohort**  **(in 1000s)** | **Increase in lifetime earnings in USD per child (20 to 59yrs)** | **Benefits by cohorts (20 to 59yrs)** **Lifetime wages**  **(in USD millions)** |
| --- | --- | --- | --- | --- | --- |
| **LBW** | 11.3 (11.1,11.6) | 3.2 | 61.1 (18.2,101.8) | 275.2 (42.9,621.0) | 725.5 (113.1,1637.0) |
| **PTB** | 10.4 (8.7,11.9) | 5.5 | 39.4 (5.3,79.5) | 181.4 (17.3,474.0) | 478.2 (45.6,1249.5) |
| **SGA** | 20.8 (15.3,27.9) | 10 | 110.3 (39.6,214.4) | 508.2 (115.8,1266.0) | 1339.5 (305.3,3337.2) |

Impact of sub-optimal prevalences in low birthweight (LBW), preterm birth (PTB), or small for gestational age (SGA) on human capital and labor market outcomes in Malaysia.

^1^ TMRED = Theoretical Minimum Risk Exposure Distribution (TMRED)

^2^ Source: United National Population Division World Population Prospects 2019.

^3^ Country specific annual wage data from World Indicators Database. Average yearly wage was estimated to be 2/3 of the gross domestic product in 2010 constant US dollars and 2011 International dollars, adjusted for purchasing power parity.

**Maldives**

**Super region:** Southeast Asia, East Asia, and Oceania**; Sub region:** Southeast Asia

**Number of live births^3^:** 36,000

**Probability of survival to age 25^2^**: 0.98

**GDP per capita 2010 US dollars (estimated annual wage)^3^:**  7,502

**GDP per capita 2011 International dollars (estimated annual wage)^3^:**  12,684

| **Birth outcome** | **Current**  **prevalence (%)** | **TMRED^1^ (%)** | **Total school years gained per birth cohort**  **(in 1000s)** | **Increase in lifetime earnings in USD per child (20 to 59yrs)** | **Benefits by cohorts (20 to 59yrs)** **Lifetime wages**  **(in USD millions)** |
| --- | --- | --- | --- | --- | --- |
| **LBW** | 11.7 (8.0,17.9) | 3.2 | 0.8 (0.2,1.8) | 192.9 (22.5,507.4) | 6.9 (0.8,18.3) |
| **PTB** | 6.2 (3.7,9.8) | 5.5 | 0.1 (0.0,0.5) | 12.1 (0.0,142.0) | 0.4 (0.0,5.1) |
| **SGA** | 18.7 (13.9,25.0) | 10 | 1.2 (0.4,2.5) | 282.5 (46.6,763.8) | 10.2 (1.7,27.5) |

Impact of sub-optimal prevalences in low birthweight (LBW), preterm birth (PTB), or small for gestational age (SGA) on human capital and labor market outcomes in Maldives.

^1^ TMRED = Theoretical Minimum Risk Exposure Distribution (TMRED)

^2^ Source: United National Population Division World Population Prospects 2019.

^3^ Country specific annual wage data from World Indicators Database. Average yearly wage was estimated to be 2/3 of the gross domestic product in 2010 constant US dollars and 2011 International dollars, adjusted for purchasing power parity.

**Mali**

**Super region:** Sub-Saharan Africa**; Sub region:** Western Sub-Saharan Africa

**Number of live births^3^:** 3,937,000

**Probability of survival to age 25^2^**: 0.82

**GDP per capita 2010 US dollars (estimated annual wage)^3^:**  727

**GDP per capita 2011 International dollars (estimated annual wage)^3^:**  1,922

| **Birth outcome** | **Current**  **prevalence (%)** | **TMRED^1^ (%)** | **Total school years gained per birth cohort**  **(in 1000s)** | **Increase in lifetime earnings in USD per child (20 to 59yrs)** | **Benefits by cohorts (20 to 59yrs)** **Lifetime wages**  **(in USD millions)** |
| --- | --- | --- | --- | --- | --- |
| **LBW** | 18.7 (13.3,24.1) | 3.2 | 142.6 (39.2,259.1) | 29.7 (7.4,58.2) | 116.8 (29.1,229.0) |
| **PTB** | 12.0 (8.6,16.7) | 5.5 | 61.5 (7.5,140.4) | 12.5 (1.4,31.3) | 49.3 (5.3,123.4) |
| **SGA** | 35.2 (30.2,40.9) | 10 | 332.0 (140.4,525.5) | 68.3 (27.3,121.0) | 269.1 (107.5,476.6) |

Impact of sub-optimal prevalences in low birthweight (LBW), preterm birth (PTB), or small for gestational age (SGA) on human capital and labor market outcomes in Mali.

^1^ TMRED = Theoretical Minimum Risk Exposure Distribution (TMRED)

^2^ Source: United National Population Division World Population Prospects 2019.

^3^ Country specific annual wage data from World Indicators Database. Average yearly wage was estimated to be 2/3 of the gross domestic product in 2010 constant US dollars and 2011 International dollars, adjusted for purchasing power parity.

**Mauritania**

**Super region:** Sub-Saharan Africa**; Sub region:** Western Sub-Saharan Africa

**Number of live births^3^:** 736,000

**Probability of survival to age 25^2^**: 0.89

**GDP per capita 2010 US dollars (estimated annual wage)^3^:**  1,349

**GDP per capita 2011 International dollars (estimated annual wage)^3^:**  3,723

| **Birth outcome** | **Current**  **prevalence (%)** | **TMRED^1^ (%)** | **Total school years gained per birth cohort**  **(in 1000s)** | **Increase in lifetime earnings in USD per child (20 to 59yrs)** | **Benefits by cohorts (20 to 59yrs)** **Lifetime wages**  **(in USD millions)** |
| --- | --- | --- | --- | --- | --- |
| **LBW** | 33.7 (28.4,39.0) | 3.2 | 57.0 (17.2,100.2) | 115.9 (34.3,212.9) | 85.3 (25.2,156.7) |
| **PTB** | 12.0 (8.6,16.7) | 5.5 | 12.7 (1.5,29.1) | 25.4 (2.8,62.4) | 18.7 (2.1,45.9) |
| **SGA** | 47.0 (39.1,54.9) | 10 | 98.0 (45.3,158.6) | 198.8 (87.6,349.1) | 146.3 (64.5,257.0) |

Impact of sub-optimal prevalences in low birthweight (LBW), preterm birth (PTB), or small for gestational age (SGA) on human capital and labor market outcomes in Mauritania.

^1^ TMRED = Theoretical Minimum Risk Exposure Distribution (TMRED)

^2^ Source: United National Population Division World Population Prospects 2019.

^3^ Country specific annual wage data from World Indicators Database. Average yearly wage was estimated to be 2/3 of the gross domestic product in 2010 constant US dollars and 2011 International dollars, adjusted for purchasing power parity.

**Mauritius**

**Super region:** Southeast Asia, East Asia, and Oceania**; Sub region:** Southeast Asia

**Number of live births^3^:** 65,000

**Probability of survival to age 25^2^**: 0.97

**GDP per capita 2010 US dollars (estimated annual wage)^3^:**  9,477

**GDP per capita 2011 International dollars (estimated annual wage)^3^:**  18,879

| **Birth outcome** | **Current**  **prevalence (%)** | **TMRED^1^ (%)** | **Total school years gained per birth cohort**  **(in 1000s)** | **Increase in lifetime earnings in USD per child (20 to 59yrs)** | **Benefits by cohorts (20 to 59yrs)** **Lifetime wages**  **(in USD millions)** |
| --- | --- | --- | --- | --- | --- |
| **LBW** | 13.9 (8.0,19.9) | 3.2 | 1.9 (0.4,3.9) | 292.7 (34.3,869.6) | 19.0 (2.2,56.5) |
| **PTB** | 12.0 (8.6,16.7) | 5.5 | 1.2 (0.2,3.0) | 199.2 (18.6,641.0) | 12.9 (1.2,41.7) |
| **SGA** | 20.4 (14.7,25.9) | 10 | 2.6 (0.9,5.1) | 437.2 (100.0,1037.7) | 28.4 (6.5,67.4) |

Impact of sub-optimal prevalences in low birthweight (LBW), preterm birth (PTB), or small for gestational age (SGA) on human capital and labor market outcomes in Mauritius.

^1^ TMRED = Theoretical Minimum Risk Exposure Distribution (TMRED)

^2^ Source: United National Population Division World Population Prospects 2019.

^3^ Country specific annual wage data from World Indicators Database. Average yearly wage was estimated to be 2/3 of the gross domestic product in 2010 constant US dollars and 2011 International dollars, adjusted for purchasing power parity.

**Mexico**

**Super region:** Latin America and Caribbean**; Sub region:** Central Latin America

**Number of live births^3^:** 11,120,000

**Probability of survival to age 25^2^**: 0.96

**GDP per capita 2010 US dollars (estimated annual wage)^3^:**  10,037

**GDP per capita 2011 International dollars (estimated annual wage)^3^:**  17,495

| **Birth outcome** | **Current**  **prevalence (%)** | **TMRED^1^ (%)** | **Total school years gained per birth cohort**  **(in 1000s)** | **Increase in lifetime earnings in USD per child (20 to 59yrs)** | **Benefits by cohorts (20 to 59yrs)** **Lifetime wages**  **(in USD millions)** |
| --- | --- | --- | --- | --- | --- |
| **LBW** | 7.9 (6.2,10.2) | 3.2 | 141.9 (40.8,285.9) | 317.1 (82.6,736.7) | 3525.9 (918.4,8192.3) |
| **PTB** | 7.0 (5.6,8.7) | 5.5 | 46.6 (0.0,135.0) | 108.1 (0.0,344.5) | 1202.0 (0.0,3830.4) |
| **SGA** | 9.9 (7.1,13.5) | 10 | 0.0 (0.0,146.6) | 0.0 (0.0,344.1) | 0.0 (0.0,3826.7) |

Impact of sub-optimal prevalences in low birthweight (LBW), preterm birth (PTB), or small for gestational age (SGA) on human capital and labor market outcomes in Mexico.

^1^ TMRED = Theoretical Minimum Risk Exposure Distribution (TMRED)

^2^ Source: United National Population Division World Population Prospects 2019.

^3^ Country specific annual wage data from World Indicators Database. Average yearly wage was estimated to be 2/3 of the gross domestic product in 2010 constant US dollars and 2011 International dollars, adjusted for purchasing power parity.

**Micronesia**

**Super region:** Southeast Asia, East Asia, and Oceania**; Sub region:** Oceania

**Number of live births^3^:** 13,000

**Probability of survival to age 25^2^**: 0.94

**GDP per capita 2010 US dollars (estimated annual wage)^3^:**  2,723

**GDP per capita 2011 International dollars (estimated annual wage)^3^:**  3,137

| **Birth outcome** | **Current**  **prevalence (%)** | **TMRED^1^ (%)** | **Total school years gained per birth cohort**  **(in 1000s)** | **Increase in lifetime earnings in USD per child (20 to 59yrs)** | **Benefits by cohorts (20 to 59yrs)** **Lifetime wages**  **(in USD millions)** |
| --- | --- | --- | --- | --- | --- |
| **LBW** | 11.1 (8.4,13.8) | 3.2 | 0.3 (0.1,0.5) | 61.2 (13.3,139.3) | 0.8 (0.2,1.8) |
| **PTB** | 10.0 (7.9,12.7) | 5.5 | 0.2 (0.0,0.3) | 36.7 (4.6,90.5) | 0.5 (0.1,1.2) |
| **SGA** | 23.2 (17.9,29.7) | 10 | 0.6 (0.3,1.2) | 142.6 (45.0,326.9) | 1.9 (0.6,4.2) |

Impact of sub-optimal prevalences in low birthweight (LBW), preterm birth (PTB), or small for gestational age (SGA) on human capital and labor market outcomes in Micronesia.

^1^ TMRED = Theoretical Minimum Risk Exposure Distribution (TMRED)

^2^ Source: United National Population Division World Population Prospects 2019.

^3^ Country specific annual wage data from World Indicators Database. Average yearly wage was estimated to be 2/3 of the gross domestic product in 2010 constant US dollars and 2011 International dollars, adjusted for purchasing power parity.

**Mongolia**

**Super region:** Central Europe, Eastern Europe, Central Asia**; Sub region:** Central Asia

**Number of live births^3^:** 384,000

**Probability of survival to age 25^2^**: 0.96

**GDP per capita 2010 US dollars (estimated annual wage)^3^:**  3,895

**GDP per capita 2011 International dollars (estimated annual wage)^3^:**  11,330

| **Birth outcome** | **Current**  **prevalence (%)** | **TMRED^1^ (%)** | **Total school years gained per birth cohort**  **(in 1000s)** | **Increase in lifetime earnings in USD per child (20 to 59yrs)** | **Benefits by cohorts (20 to 59yrs)** **Lifetime wages**  **(in USD millions)** |
| --- | --- | --- | --- | --- | --- |
| **LBW** | 5.4 (4.2,6.9) | 3.2 | 2.3 (0.4,4.6) | 20.0 (2.9,49.3) | 7.7 (1.1,18.9) |
| **PTB** | 10.4 (8.7,11.9) | 5.5 | 5.5 (0.9,11.3) | 49.8 (6.5,118.3) | 19.1 (2.5,45.4) |
| **SGA** | 10.8 (7.1,16.1) | 10 | 1.1 (0.0,9.5) | 8.7 (0.0,91.4) | 3.3 (0.0,35.1) |

Impact of sub-optimal prevalences in low birthweight (LBW), preterm birth (PTB), or small for gestational age (SGA) on human capital and labor market outcomes in Mongolia.

^1^ TMRED = Theoretical Minimum Risk Exposure Distribution (TMRED)

^2^ Source: United National Population Division World Population Prospects 2019.

^3^ Country specific annual wage data from World Indicators Database. Average yearly wage was estimated to be 2/3 of the gross domestic product in 2010 constant US dollars and 2011 International dollars, adjusted for purchasing power parity.

**Morocco**

**Super region:** North Africa and Middle East**; Sub region:** North Africa and Middle East

**Number of live births^3^:** 3,409,000

**Probability of survival to age 25^2^**: 0.97

**GDP per capita 2010 US dollars (estimated annual wage)^3^:**  3,222

**GDP per capita 2011 International dollars (estimated annual wage)^3^:**  7,325

| **Birth outcome** | **Current**  **prevalence (%)** | **TMRED^1^ (%)** | **Total school years gained per birth cohort**  **(in 1000s)** | **Increase in lifetime earnings in USD per child (20 to 59yrs)** | **Benefits by cohorts (20 to 59yrs)** **Lifetime wages**  **(in USD millions)** |
| --- | --- | --- | --- | --- | --- |
| **LBW** | 17.3 (13.9,21.9) | 3.2 | 133.4 (38.8,237.9) | 358.6 (106.8,652.2) | 1222.3 (364.1,2223.3) |
| **PTB** | 13.4 (6.3,30.9) | 5.5 | 70.6 (0.0,266.7) | 189.2 (0.0,714.1) | 645.0 (0.0,2434.3) |
| **SGA** | 7.6 (5.2,11.1) | 10 | 0.0 (0.0,5.3) | 0.0 (0.0,14.7) | 0.0 (0.0,50.0) |

Impact of sub-optimal prevalences in low birthweight (LBW), preterm birth (PTB), or small for gestational age (SGA) on human capital and labor market outcomes in Morocco.

^1^ TMRED = Theoretical Minimum Risk Exposure Distribution (TMRED)

^2^ Source: United National Population Division World Population Prospects 2019.

^3^ Country specific annual wage data from World Indicators Database. Average yearly wage was estimated to be 2/3 of the gross domestic product in 2010 constant US dollars and 2011 International dollars, adjusted for purchasing power parity.

**Mozambique**

**Super region:** Sub-Saharan Africa**; Sub region:** Eastern Sub-Saharan Africa

**Number of live births^3^:** 5,495,000

**Probability of survival to age 25^2^**: 0.87

**GDP per capita 2010 US dollars (estimated annual wage)^3^:**  529

**GDP per capita 2011 International dollars (estimated annual wage)^3^:**  1,158

| **Birth outcome** | **Current**  **prevalence (%)** | **TMRED^1^ (%)** | **Total school years gained per birth cohort**  **(in 1000s)** | **Increase in lifetime earnings in USD per child (20 to 59yrs)** | **Benefits by cohorts (20 to 59yrs)** **Lifetime wages**  **(in USD millions)** |
| --- | --- | --- | --- | --- | --- |
| **LBW** | 13.8 (10.9,17.5) | 3.2 | 144.5 (42.2,260.5) | 27.9 (8.5,51.0) | 153.2 (46.6,280.2) |
| **PTB** | 12.0 (8.6,16.7) | 5.5 | 92.3 (15.8,208.2) | 17.6 (3.0,41.7) | 96.8 (16.4,229.3) |
| **SGA** | 27.9 (23.6,31.7) | 10 | 349.1 (153.8,566.8) | 66.9 (29.5,115.4) | 367.4 (162.1,634.1) |

Impact of sub-optimal prevalences in low birthweight (LBW), preterm birth (PTB), or small for gestational age (SGA) on human capital and labor market outcomes in Mozambique.

^1^ TMRED = Theoretical Minimum Risk Exposure Distribution (TMRED)

^2^ Source: United National Population Division World Population Prospects 2019.

^3^ Country specific annual wage data from World Indicators Database. Average yearly wage was estimated to be 2/3 of the gross domestic product in 2010 constant US dollars and 2011 International dollars, adjusted for purchasing power parity.

**Myanmar**

**Super region:** Southeast Asia, East Asia, and Oceania**; Sub region:** Southeast Asia

**Number of live births^3^:** 4,738,000

**Probability of survival to age 25^2^**: 0.92

**GDP per capita 2010 US dollars (estimated annual wage)^3^:**  1,335

**GDP per capita 2011 International dollars (estimated annual wage)^3^:**  5,030

| **Birth outcome** | **Current**  **prevalence (%)** | **TMRED^1^ (%)** | **Total school years gained per birth cohort**  **(in 1000s)** | **Increase in lifetime earnings in USD per child (20 to 59yrs)** | **Benefits by cohorts (20 to 59yrs)** **Lifetime wages**  **(in USD millions)** |
| --- | --- | --- | --- | --- | --- |
| **LBW** | 12.3 (8.5,15.9) | 3.2 | 111.3 (29.9,213.9) | 34.7 (5.4,86.3) | 164.5 (25.5,409.0) |
| **PTB** | 10.4 (8.7,11.9) | 5.5 | 65.6 (9.7,135.6) | 20.5 (2.4,53.6) | 97.0 (11.2,254.0) |
| **SGA** | 32.1 (27.0,38.3) | 10 | 388.0 (174.3,627.0) | 124.7 (33.1,271.6) | 590.7 (156.9,1286.7) |

Impact of sub-optimal prevalences in low birthweight (LBW), preterm birth (PTB), or small for gestational age (SGA) on human capital and labor market outcomes in Myanmar.

^1^ TMRED = Theoretical Minimum Risk Exposure Distribution (TMRED)

^2^ Source: United National Population Division World Population Prospects 2019.

^3^ Country specific annual wage data from World Indicators Database. Average yearly wage was estimated to be 2/3 of the gross domestic product in 2010 constant US dollars and 2011 International dollars, adjusted for purchasing power parity.

**Namibia**

**Super region:** Sub-Saharan Africa**; Sub region:** Southern Sub-Saharan Africa

**Number of live births^3^:** 350,000

**Probability of survival to age 25^2^**: 0.91

**GDP per capita 2010 US dollars (estimated annual wage)^3^:**  6,412

**GDP per capita 2011 International dollars (estimated annual wage)^3^:**  10,450

| **Birth outcome** | **Current**  **prevalence (%)** | **TMRED^1^ (%)** | **Total school years gained per birth cohort**  **(in 1000s)** | **Increase in lifetime earnings in USD per child (20 to 59yrs)** | **Benefits by cohorts (20 to 59yrs)** **Lifetime wages**  **(in USD millions)** |
| --- | --- | --- | --- | --- | --- |
| **LBW** | 15.5 (12.3,19.7) | 3.2 | 11.2 (3.4,20.5) | 592.2 (175.1,1080.2) | 207.3 (61.3,378.1) |
| **PTB** | 12.0 (8.6,16.7) | 5.5 | 6.1 (0.9,14.0) | 322.0 (44.3,732.2) | 112.7 (15.5,256.3) |
| **SGA** | 24.6 (18.9,29.4) | 10 | 18.5 (8.2,31.1) | 975.8 (429.0,1645.8) | 341.5 (150.1,576.0) |

Impact of sub-optimal prevalences in low birthweight (LBW), preterm birth (PTB), or small for gestational age (SGA) on human capital and labor market outcomes in Namibia.

^1^ TMRED = Theoretical Minimum Risk Exposure Distribution (TMRED)

^2^ Source: United National Population Division World Population Prospects 2019.

^3^ Country specific annual wage data from World Indicators Database. Average yearly wage was estimated to be 2/3 of the gross domestic product in 2010 constant US dollars and 2011 International dollars, adjusted for purchasing power parity.

**Nepal**

**Super region:** South Asia**; Sub region:** South Asia

**Number of live births^3^:** 2,810,000

**Probability of survival to age 25^2^**: 0.95

**GDP per capita 2010 US dollars (estimated annual wage)^3^:**  732

**GDP per capita 2011 International dollars (estimated annual wage)^3^:**  2,455

| **Birth outcome** | **Current**  **prevalence (%)** | **TMRED^1^ (%)** | **Total school years gained per birth cohort**  **(in 1000s)** | **Increase in lifetime earnings in USD per child (20 to 59yrs)** | **Benefits by cohorts (20 to 59yrs)** **Lifetime wages**  **(in USD millions)** |
| --- | --- | --- | --- | --- | --- |
| **LBW** | 21.8 (15.2,30.3) | 3.2 | 140.1 (43.1,276.9) | 51.1 (15.2,102.9) | 143.5 (42.8,289.1) |
| **PTB** | 5.3 (3.6,7.4) | 5.5 | 0.0 (0.0,16.2) | 0.0 (0.0,5.9) | 0.0 (0.0,16.7) |
| **SGA** | 39.3 (34.7,44.4) | 10 | 314.4 (149.0,494.2) | 116.6 (54.3,182.0) | 327.7 (152.5,511.4) |

Impact of sub-optimal prevalences in low birthweight (LBW), preterm birth (PTB), or small for gestational age (SGA) on human capital and labor market outcomes in Nepal.

^1^ TMRED = Theoretical Minimum Risk Exposure Distribution (TMRED)

^2^ Source: United National Population Division World Population Prospects 2019.

^3^ Country specific annual wage data from World Indicators Database. Average yearly wage was estimated to be 2/3 of the gross domestic product in 2010 constant US dollars and 2011 International dollars, adjusted for purchasing power parity.

**Nicaragua**

**Super region:** Latin America and Caribbean**; Sub region:** Central Latin America

**Number of live births^3^:** 670,000

**Probability of survival to age 25^2^**: 0.95

**GDP per capita 2010 US dollars (estimated annual wage)^3^:**  1,836

**GDP per capita 2011 International dollars (estimated annual wage)^3^:**  4,846

| **Birth outcome** | **Current**  **prevalence (%)** | **TMRED^1^ (%)** | **Total school years gained per birth cohort**  **(in 1000s)** | **Increase in lifetime earnings in USD per child (20 to 59yrs)** | **Benefits by cohorts (20 to 59yrs)** **Lifetime wages**  **(in USD millions)** |
| --- | --- | --- | --- | --- | --- |
| **LBW** | 10.7 (8.4,13.2) | 3.2 | 13.5 (3.9,25.2) | 47.9 (13.5,90.1) | 32.1 (9.1,60.4) |
| **PTB** | 9.8 (8.6,11.3) | 5.5 | 8.5 (1.1,16.9) | 30.3 (4.0,60.1) | 20.3 (2.7,40.3) |
| **SGA** | 14.5 (10.8,19.5) | 10 | 11.4 (0.3,26.8) | 39.8 (1.1,95.6) | 26.7 (0.7,64.0) |

Impact of sub-optimal prevalences in low birthweight (LBW), preterm birth (PTB), or small for gestational age (SGA) on human capital and labor market outcomes in Nicaragua.

^1^ TMRED = Theoretical Minimum Risk Exposure Distribution (TMRED)

^2^ Source: United National Population Division World Population Prospects 2019.

^3^ Country specific annual wage data from World Indicators Database. Average yearly wage was estimated to be 2/3 of the gross domestic product in 2010 constant US dollars and 2011 International dollars, adjusted for purchasing power parity.

**Niger**

**Super region:** Sub-Saharan Africa**; Sub region:** Western Sub-Saharan Africa

**Number of live births^3^:** 5,114,000

**Probability of survival to age 25^2^**: 0.86

**GDP per capita 2010 US dollars (estimated annual wage)^3^:**  386

**GDP per capita 2011 International dollars (estimated annual wage)^3^:**  903

| **Birth outcome** | **Current**  **prevalence (%)** | **TMRED^1^ (%)** | **Total school years gained per birth cohort**  **(in 1000s)** | **Increase in lifetime earnings in USD per child (20 to 59yrs)** | **Benefits by cohorts (20 to 59yrs)** **Lifetime wages**  **(in USD millions)** |
| --- | --- | --- | --- | --- | --- |
| **LBW** | 26.7 (21.4,32.0) | 3.2 | 291.3 (87.7,523.4) | 33.5 (10.0,62.2) | 171.2 (51.1,318.0) |
| **PTB** | 12.0 (8.6,16.7) | 5.5 | 85.0 (11.2,200.1) | 9.7 (1.3,24.7) | 49.8 (6.6,126.4) |
| **SGA** | 38.1 (31.2,44.9) | 10 | 495.2 (223.3,814.9) | 57.3 (26.7,95.7) | 292.8 (136.7,489.6) |

Impact of sub-optimal prevalences in low birthweight (LBW), preterm birth (PTB), or small for gestational age (SGA) on human capital and labor market outcomes in Niger.

^1^ TMRED = Theoretical Minimum Risk Exposure Distribution (TMRED)

^2^ Source: United National Population Division World Population Prospects 2019.

^3^ Country specific annual wage data from World Indicators Database. Average yearly wage was estimated to be 2/3 of the gross domestic product in 2010 constant US dollars and 2011 International dollars, adjusted for purchasing power parity.

**Nigeria**

**Super region:** Sub-Saharan Africa**; Sub region:** Western Sub-Saharan Africa

**Number of live births^3^:** 36,886,000

**Probability of survival to age 25^2^**: 0.78

**GDP per capita 2010 US dollars (estimated annual wage)^3^:**  2,563

**GDP per capita 2011 International dollars (estimated annual wage)^3^:**  5,686

| **Birth outcome** | **Current**  **prevalence (%)** | **TMRED^1^ (%)** | **Total school years gained per birth cohort**  **(in 1000s)** | **Increase in lifetime earnings in USD per child (20 to 59yrs)** | **Benefits by cohorts (20 to 59yrs)** **Lifetime wages**  **(in USD millions)** |
| --- | --- | --- | --- | --- | --- |
| **LBW** | 11.7 (6.3,17.1) | 3.2 | 677.2 (133.1,1502.3) | 34.5 (7.5,75.9) | 1272.5 (277.8,2800.4) |
| **PTB** | 11.4 (8.0,15.7) | 5.5 | 494.0 (40.8,1206.9) | 25.5 (2.0,61.1) | 940.4 (73.6,2252.7) |
| **SGA** | 23.7 (20.2,27.1) | 10 | 1595.8 (738.1,2569.0) | 80.4 (35.9,136.7) | 2964.5 (1322.7,5043.4) |

Impact of sub-optimal prevalences in low birthweight (LBW), preterm birth (PTB), or small for gestational age (SGA) on human capital and labor market outcomes in Nigeria  .

^1^ TMRED = Theoretical Minimum Risk Exposure Distribution (TMRED)

^2^ Source: United National Population Division World Population Prospects 2019.

^3^ Country specific annual wage data from World Indicators Database. Average yearly wage was estimated to be 2/3 of the gross domestic product in 2010 constant US dollars and 2011 International dollars, adjusted for purchasing power parity.

**Pakistan**

**Super region:** South Asia**; Sub region:** South Asia

**Number of live births^3^:** 29,970,000

**Probability of survival to age 25^2^**: 0.90

**GDP per capita 2010 US dollars (estimated annual wage)^3^:**  1,083

**GDP per capita 2011 International dollars (estimated annual wage)^3^:**  4,459

| **Birth outcome** | **Current**  **prevalence (%)** | **TMRED^1^ (%)** | **Total school years gained per birth cohort**  **(in 1000s)** | **Increase in lifetime earnings in USD per child (20 to 59yrs)** | **Benefits by cohorts (20 to 59yrs)** **Lifetime wages**  **(in USD millions)** |
| --- | --- | --- | --- | --- | --- |
| **LBW** | 26.0 (16.6,35.5) | 3.2 | 1736.3 (410.0,3272.0) | 93.5 (24.4,183.8) | 2803.1 (731.3,5507.9) |
| **PTB** | 8.4 (5.6,11.9) | 5.5 | 215.8 (0.0,695.5) | 11.7 (0.0,38.6) | 351.9 (0.0,1158.1) |
| **SGA** | 47.0 (42.8,53.8) | 10 | 4057.6 (1868.4,6425.9) | 222.3 (104.6,358.0) | 6661.1 (3134.3,10730.0) |

Impact of sub-optimal prevalences in low birthweight (LBW), preterm birth (PTB), or small for gestational age (SGA) on human capital and labor market outcomes in Pakistan  .

^1^ TMRED = Theoretical Minimum Risk Exposure Distribution (TMRED)

^2^ Source: United National Population Division World Population Prospects 2019.

^3^ Country specific annual wage data from World Indicators Database. Average yearly wage was estimated to be 2/3 of the gross domestic product in 2010 constant US dollars and 2011 International dollars, adjusted for purchasing power parity.

**Palestine**

**Super region:** North Africa and Middle East**; Sub region:** North Africa and Middle East

**Number of live births^3^:** 708,000

**Probability of survival to age 25^2^**: 0.97

**GDP per capita 2010 US dollars (estimated annual wage)^3^:**  2,632

**GDP per capita 2011 International dollars (estimated annual wage)^3^:**  4,494

| **Birth outcome** | **Current**  **prevalence (%)** | **TMRED^1^ (%)** | **Total school years gained per birth cohort**  **(in 1000s)** | **Increase in lifetime earnings in USD per child (20 to 59yrs)** | **Benefits by cohorts (20 to 59yrs)** **Lifetime wages**  **(in USD millions)** |
| --- | --- | --- | --- | --- | --- |
| **LBW** | 10.9 (6.9,14.9) | 3.2 | 14.4 (4.3,30.1) | 25.9 (7.6,53.8) | 18.3 (5.4,38.1) |
| **PTB** | 10.3 (9.1,11.6) | 5.5 | 10.1 (1.3,20.3) | 17.9 (2.4,36.3) | 12.7 (1.7,25.7) |
| **SGA** | 13.2 (9.7,17.4) | 10 | 8.8 (0.0,23.3) | 15.6 (0.0,41.7) | 11.0 (0.0,29.6) |

Impact of sub-optimal prevalences in low birthweight (LBW), preterm birth (PTB), or small for gestational age (SGA) on human capital and labor market outcomes in Palestine.

^1^ TMRED = Theoretical Minimum Risk Exposure Distribution (TMRED)

^2^ Source: United National Population Division World Population Prospects 2019.

^3^ Country specific annual wage data from World Indicators Database. Average yearly wage was estimated to be 2/3 of the gross domestic product in 2010 constant US dollars and 2011 International dollars, adjusted for purchasing power parity.

**Papua New Guinea**

**Super region:** Southeast Asia, East Asia, and Oceania**; Sub region:** Oceania

**Number of live births^3^:** 1,161,000

**Probability of survival to age 25^2^**: 0.91

**GDP per capita 2010 US dollars (estimated annual wage)^3^:**  2,400

**GDP per capita 2011 International dollars (estimated annual wage)^3^:**  3,820

| **Birth outcome** | **Current**  **prevalence (%)** | **TMRED^1^ (%)** | **Total school years gained per birth cohort**  **(in 1000s)** | **Increase in lifetime earnings in USD per child (20 to 59yrs)** | **Benefits by cohorts (20 to 59yrs)** **Lifetime wages**  **(in USD millions)** |
| --- | --- | --- | --- | --- | --- |
| **LBW** | 10.0 (7.3,12.7) | 3.2 | 20.3 (5.8,37.1) | 44.4 (10.9,105.4) | 51.6 (12.6,122.3) |
| **PTB** | 10.0 (7.9,12.7) | 5.5 | 14.1 (1.6,32.2) | 30.9 (3.3,83.7) | 35.9 (3.8,97.2) |
| **SGA** | 21.6 (16.6,28.2) | 10 | 48.3 (18.3,91.7) | 108.3 (31.1,253.2) | 125.8 (36.1,293.9) |

Impact of sub-optimal prevalences in low birthweight (LBW), preterm birth (PTB), or small for gestational age (SGA) on human capital and labor market outcomes in Papua New Guinea  .

^1^ TMRED = Theoretical Minimum Risk Exposure Distribution (TMRED)

^2^ Source: United National Population Division World Population Prospects 2019.

^3^ Country specific annual wage data from World Indicators Database. Average yearly wage was estimated to be 2/3 of the gross domestic product in 2010 constant US dollars and 2011 International dollars, adjusted for purchasing power parity.

**Paraguay**

**Super region:** Latin America and Caribbean**; Sub region:** Tropical Latin America

**Number of live births^3^:** 716,000

**Probability of survival to age 25^2^**: 0.95

**GDP per capita 2010 US dollars (estimated annual wage)^3^:**  4,944

**GDP per capita 2011 International dollars (estimated annual wage)^3^:**  11,056

| **Birth outcome** | **Current**  **prevalence (%)** | **TMRED^1^ (%)** | **Total school years gained per birth cohort**  **(in 1000s)** | **Increase in lifetime earnings in USD per child (20 to 59yrs)** | **Benefits by cohorts (20 to 59yrs)** **Lifetime wages**  **(in USD millions)** |
| --- | --- | --- | --- | --- | --- |
| **LBW** | 8.1 (6.5,10.6) | 3.2 | 9.3 (2.5,17.9) | 152.6 (40.6,303.2) | 109.2 (29.1,217.1) |
| **PTB** | 8.1 (6.4,9.9) | 5.5 | 5.1 (0.5,12.6) | 83.3 (8.3,213.4) | 59.6 (6.0,152.8) |
| **SGA** | 10.8 (7.7,14.9) | 10 | 2.2 (0.0,13.7) | 35.7 (0.0,234.8) | 25.6 (0.0,168.1) |

Impact of sub-optimal prevalences in low birthweight (LBW), preterm birth (PTB), or small for gestational age (SGA) on human capital and labor market outcomes in Paraguay.

^1^ TMRED = Theoretical Minimum Risk Exposure Distribution (TMRED)

^2^ Source: United National Population Division World Population Prospects 2019.

^3^ Country specific annual wage data from World Indicators Database. Average yearly wage was estimated to be 2/3 of the gross domestic product in 2010 constant US dollars and 2011 International dollars, adjusted for purchasing power parity.

**Peru**

**Super region:** Latin America and Caribbean**; Sub region:** Andean Latin America

**Number of live births^3^:** 2,870,000

**Probability of survival to age 25^2^**: 0.96

**GDP per capita 2010 US dollars (estimated annual wage)^3^:**  6,114

**GDP per capita 2011 International dollars (estimated annual wage)^3^:**  12,121

| **Birth outcome** | **Current**  **prevalence (%)** | **TMRED^1^ (%)** | **Total school years gained per birth cohort**  **(in 1000s)** | **Increase in lifetime earnings in USD per child (20 to 59yrs)** | **Benefits by cohorts (20 to 59yrs)** **Lifetime wages**  **(in USD millions)** |
| --- | --- | --- | --- | --- | --- |
| **LBW** | 9.4 (7.2,12.2) | 3.2 | 47.7 (13.8,95.0) | 112.9 (11.1,324.4) | 324.1 (31.8,931.1) |
| **PTB** | 8.8 (6.9,11.0) | 5.5 | 26.6 (2.5,66.3) | 62.6 (3.4,224.2) | 179.8 (9.6,643.5) |
| **SGA** | 11.4 (8.1,15.1) | 10 | 13.6 (0.0,61.1) | 28.9 (0.0,198.4) | 82.9 (0.0,569.5) |

Impact of sub-optimal prevalences in low birthweight (LBW), preterm birth (PTB), or small for gestational age (SGA) on human capital and labor market outcomes in Peru  .

^1^ TMRED = Theoretical Minimum Risk Exposure Distribution (TMRED)

^2^ Source: United National Population Division World Population Prospects 2019.

^3^ Country specific annual wage data from World Indicators Database. Average yearly wage was estimated to be 2/3 of the gross domestic product in 2010 constant US dollars and 2011 International dollars, adjusted for purchasing power parity.

**Philippines**

**Super region:** Southeast Asia, East Asia, and Oceania**; Sub region:** Southeast Asia

**Number of live births^3^:** 10,889,000

**Probability of survival to age 25^2^**: 0.95

**GDP per capita 2010 US dollars (estimated annual wage)^3^:**  2,605

**GDP per capita 2011 International dollars (estimated annual wage)^3^:**  6,848

| **Birth outcome** | **Current**  **prevalence (%)** | **TMRED^1^ (%)** | **Total school years gained per birth cohort**  **(in 1000s)** | **Increase in lifetime earnings in USD per child (20 to 59yrs)** | **Benefits by cohorts (20 to 59yrs)** **Lifetime wages**  **(in USD millions)** |
| --- | --- | --- | --- | --- | --- |
| **LBW** | 20.1 (14.2,26.1) | 3.2 | 497.7 (138.9,909.9) | 184.9 (45.5,412.7) | 2012.8 (495.1,4494.1) |
| **PTB** | 13.3 (9.1,18.5) | 5.5 | 232.0 (32.0,548.3) | 88.3 (12.5,231.5) | 961.3 (136.0,2520.9) |
| **SGA** | 33.6 (27.9,40.8) | 10 | 982.7 (415.6,1613.6) | 371.9 (153.6,700.9) | 4049.1 (1672.9,7631.6) |

Impact of sub-optimal prevalences in low birthweight (LBW), preterm birth (PTB), or small for gestational age (SGA) on human capital and labor market outcomes in Philippines.

^1^ TMRED = Theoretical Minimum Risk Exposure Distribution (TMRED)

^2^ Source: United National Population Division World Population Prospects 2019.

^3^ Country specific annual wage data from World Indicators Database. Average yearly wage was estimated to be 2/3 of the gross domestic product in 2010 constant US dollars and 2011 International dollars, adjusted for purchasing power parity.

**Rwanda**

**Super region:** Sub-Saharan Africa**; Sub region:** Eastern Sub-Saharan Africa

**Number of live births^3^:** 1,951,000

**Probability of survival to age 25^2^**: 0.93

**GDP per capita 2010 US dollars (estimated annual wage)^3^:**  732

**GDP per capita 2011 International dollars (estimated annual wage)^3^:**  1,774

| **Birth outcome** | **Current**  **prevalence (%)** | **TMRED^1^ (%)** | **Total school years gained per birth cohort**  **(in 1000s)** | **Increase in lifetime earnings in USD per child (20 to 59yrs)** | **Benefits by cohorts (20 to 59yrs)** **Lifetime wages**  **(in USD millions)** |
| --- | --- | --- | --- | --- | --- |
| **LBW** | 7.9 (6.2,10.1) | 3.2 | 23.3 (6.7,46.9) | 17.9 (4.9,35.8) | 34.9 (9.6,69.9) |
| **PTB** | 12.0 (8.6,16.7) | 5.5 | 35.0 (3.2,83.4) | 26.9 (2.4,66.9) | 52.4 (4.7,130.4) |
| **SGA** | 12.3 (9.4,16.4) | 10 | 15.5 (0.0,46.9) | 11.9 (0.0,36.4) | 23.2 (0.0,70.9) |

Impact of sub-optimal prevalences in low birthweight (LBW), preterm birth (PTB), or small for gestational age (SGA) on human capital and labor market outcomes in Rwanda.

^1^ TMRED = Theoretical Minimum Risk Exposure Distribution (TMRED)

^2^ Source: United National Population Division World Population Prospects 2019.

^3^ Country specific annual wage data from World Indicators Database. Average yearly wage was estimated to be 2/3 of the gross domestic product in 2010 constant US dollars and 2011 International dollars, adjusted for purchasing power parity.

**Saint Vincent and the Grenadines**

**Super region:** Latin America and Caribbean**; Sub region:** Caribbean

**Number of live births^3^:** 8,000

**Probability of survival to age 25^2^**: 0.96

**GDP per capita 2010 US dollars (estimated annual wage)^3^:**  6,582

**GDP per capita 2011 International dollars (estimated annual wage)^3^:**  10,472

| **Birth outcome** | **Current**  **prevalence (%)** | **TMRED^1^ (%)** | **Total school years gained per birth cohort**  **(in 1000s)** | **Increase in lifetime earnings in USD per child (20 to 59yrs)** | **Benefits by cohorts (20 to 59yrs)** **Lifetime wages**  **(in USD millions)** |
| --- | --- | --- | --- | --- | --- |
| **LBW** | 7.9 (4.2,11.7) | 3.2 | 0.1 (0.0,0.2) | 139.7 (17.4,332.7) | 1.1 (0.1,2.7) |
| **PTB** | 9.8 (8.6,11.3) | 5.5 | 0.1 (0.0,0.2) | 149.1 (22.0,294.5) | 1.2 (0.2,2.4) |
| **SGA** | 13.4 (10.3,18.2) | 10 | 0.1 (0.0,0.3) | 147.0 (0.0,393.6) | 1.2 (0.0,3.1) |

Impact of sub-optimal prevalences in low birthweight (LBW), preterm birth (PTB), or small for gestational age (SGA) on human capital and labor market outcomes in Saint Vincent and the Grenadines.

^1^ TMRED = Theoretical Minimum Risk Exposure Distribution (TMRED)

^2^ Source: United National Population Division World Population Prospects 2019.

^3^ Country specific annual wage data from World Indicators Database. Average yearly wage was estimated to be 2/3 of the gross domestic product in 2010 constant US dollars and 2011 International dollars, adjusted for purchasing power parity.

**Samoa**

**Super region:** Southeast Asia, East Asia, and Oceania**; Sub region:** Oceania

**Number of live births^3^:** 24,000

**Probability of survival to age 25^2^**: 0.97

**GDP per capita 2010 US dollars (estimated annual wage)^3^:**  3,560

**GDP per capita 2011 International dollars (estimated annual wage)^3^:**  5,567

| **Birth outcome** | **Current**  **prevalence (%)** | **TMRED^1^ (%)** | **Total school years gained per birth cohort**  **(in 1000s)** | **Increase in lifetime earnings in USD per child (20 to 59yrs)** | **Benefits by cohorts (20 to 59yrs)** **Lifetime wages**  **(in USD millions)** |
| --- | --- | --- | --- | --- | --- |
| **LBW** | 1.2 (-1.4,3.9) | 3.2 | 0.0 (0.0,0.0) | 0.0 (0.0,7.6) | 0.0 (0.0,0.2) |
| **PTB** | 10.0 (7.9,12.7) | 5.5 | 0.3 (0.0,0.7) | 47.7 (5.9,130.3) | 1.1 (0.1,3.1) |
| **SGA** | 3.5 (1.9,7.1) | 10 | 0.0 (0.0,0.0) | 0.0 (0.0,0.0) | 0.0 (0.0,0.0) |

Impact of sub-optimal prevalences in low birthweight (LBW), preterm birth (PTB), or small for gestational age (SGA) on human capital and labor market outcomes in Samoa.

^1^ TMRED = Theoretical Minimum Risk Exposure Distribution (TMRED)

^2^ Source: United National Population Division World Population Prospects 2019.

^3^ Country specific annual wage data from World Indicators Database. Average yearly wage was estimated to be 2/3 of the gross domestic product in 2010 constant US dollars and 2011 International dollars, adjusted for purchasing power parity.

**São Tomé and Príncipe**

**Super region:** Sub-Saharan Africa**; Sub region:** Western Sub-Saharan Africa

**Number of live births^3^:** 33,000

**Probability of survival to age 25^2^**: 0.95

**GDP per capita 2010 US dollars (estimated annual wage)^3^:**  1,236

**GDP per capita 2011 International dollars (estimated annual wage)^3^:**  2,890

| **Birth outcome** | **Current**  **prevalence (%)** | **TMRED^1^ (%)** | **Total school years gained per birth cohort**  **(in 1000s)** | **Increase in lifetime earnings in USD per child (20 to 59yrs)** | **Benefits by cohorts (20 to 59yrs)** **Lifetime wages**  **(in USD millions)** |
| --- | --- | --- | --- | --- | --- |
| **LBW** | 6.6 (5.0,8.6) | 3.2 | 0.3 (0.1,0.6) | 11.9 (3.1,26.1) | 0.4 (0.1,0.9) |
| **PTB** | 12.0 (8.6,16.7) | 5.5 | 0.6 (0.1,1.5) | 24.4 (3.6,65.3) | 0.8 (0.1,2.2) |
| **SGA** | 15.5 (12.5,19.4) | 10 | 0.7 (0.2,1.3) | 27.3 (8.1,58.2) | 0.9 (0.3,1.9) |

Impact of sub-optimal prevalences in low birthweight (LBW), preterm birth (PTB), or small for gestational age (SGA) on human capital and labor market outcomes in São Tomé and Príncipe.

^1^ TMRED = Theoretical Minimum Risk Exposure Distribution (TMRED)

^2^ Source: United National Population Division World Population Prospects 2019.

^3^ Country specific annual wage data from World Indicators Database. Average yearly wage was estimated to be 2/3 of the gross domestic product in 2010 constant US dollars and 2011 International dollars, adjusted for purchasing power parity.

**Senegal**

**Super region:** Sub-Saharan Africa**; Sub region:** Western Sub-Saharan Africa

**Number of live births^3^:** 2,720,000

**Probability of survival to age 25^2^**: 0.92

**GDP per capita 2010 US dollars (estimated annual wage)^3^:**  1,383

**GDP per capita 2011 International dollars (estimated annual wage)^3^:**  3,002

| **Birth outcome** | **Current**  **prevalence (%)** | **TMRED^1^ (%)** | **Total school years gained per birth cohort**  **(in 1000s)** | **Increase in lifetime earnings in USD per child (20 to 59yrs)** | **Benefits by cohorts (20 to 59yrs)** **Lifetime wages**  **(in USD millions)** |
| --- | --- | --- | --- | --- | --- |
| **LBW** | 18.5 (14.1,24.0) | 3.2 | 107.4 (29.3,197.4) | 61.2 (16.8,119.9) | 166.4 (45.7,326.1) |
| **PTB** | 12.0 (8.6,16.7) | 5.5 | 47.3 (6.5,116.1) | 27.0 (3.6,68.6) | 73.5 (9.7,186.7) |
| **SGA** | 29.0 (23.5,34.0) | 10 | 194.3 (87.5,320.3) | 109.2 (46.2,194.0) | 297.1 (125.6,527.7) |

Impact of sub-optimal prevalences in low birthweight (LBW), preterm birth (PTB), or small for gestational age (SGA) on human capital and labor market outcomes in Senegal.

^1^ TMRED = Theoretical Minimum Risk Exposure Distribution (TMRED)

^2^ Source: United National Population Division World Population Prospects 2019.

^3^ Country specific annual wage data from World Indicators Database. Average yearly wage was estimated to be 2/3 of the gross domestic product in 2010 constant US dollars and 2011 International dollars, adjusted for purchasing power parity.

**Sierra Leone**

**Super region:** Sub-Saharan Africa**; Sub region:** Western Sub-Saharan Africa

**Number of live births^3^:** 1,277,000

**Probability of survival to age 25^2^**: 0.79

**GDP per capita 2010 US dollars (estimated annual wage)^3^:**  441

**GDP per capita 2011 International dollars (estimated annual wage)^3^:**  1,326

| **Birth outcome** | **Current**  **prevalence (%)** | **TMRED^1^ (%)** | **Total school years gained per birth cohort**  **(in 1000s)** | **Increase in lifetime earnings in USD per child (20 to 59yrs)** | **Benefits by cohorts (20 to 59yrs)** **Lifetime wages**  **(in USD millions)** |
| --- | --- | --- | --- | --- | --- |
| **LBW** | 14.4 (11.4,18.1) | 3.2 | 31.9 (8.6,58.5) | 12.1 (3.3,23.3) | 15.4 (4.2,29.7) |
| **PTB** | 12.0 (8.6,16.7) | 5.5 | 19.7 (2.1,47.6) | 7.5 (0.8,20.0) | 9.6 (1.0,25.6) |
| **SGA** | 28.4 (24.2,32.7) | 10 | 76.1 (34.3,120.5) | 28.9 (13.0,50.2) | 36.9 (16.6,64.1) |

Impact of sub-optimal prevalences in low birthweight (LBW), preterm birth (PTB), or small for gestational age (SGA) on human capital and labor market outcomes in Sierra Leone.

^1^ TMRED = Theoretical Minimum Risk Exposure Distribution (TMRED)

^2^ Source: United National Population Division World Population Prospects 2019.

^3^ Country specific annual wage data from World Indicators Database. Average yearly wage was estimated to be 2/3 of the gross domestic product in 2010 constant US dollars and 2011 International dollars, adjusted for purchasing power parity.

**Solomon Islands**

**Super region:** Southeast Asia, East Asia, and Oceania**; Sub region:** Oceania

**Number of live births^3^:** 105,000

**Probability of survival to age 25^2^**: 0.96

**GDP per capita 2010 US dollars (estimated annual wage)^3^:**  1,444

**GDP per capita 2011 International dollars (estimated annual wage)^3^:**  2,094

| **Birth outcome** | **Current**  **prevalence (%)** | **TMRED^1^ (%)** | **Total school years gained per birth cohort**  **(in 1000s)** | **Increase in lifetime earnings in USD per child (20 to 59yrs)** | **Benefits by cohorts (20 to 59yrs)** **Lifetime wages**  **(in USD millions)** |
| --- | --- | --- | --- | --- | --- |
| **LBW** | 12.5 (9.8,15.2) | 3.2 | 2.6 (0.8,4.8) | 37.7 (7.7,82.7) | 4.0 (0.8,8.7) |
| **PTB** | 10.0 (7.9,12.7) | 5.5 | 1.4 (0.2,3.1) | 19.4 (2.1,55.5) | 2.0 (0.2,5.8) |
| **SGA** | 24.0 (18.9,30.3) | 10 | 5.6 (2.5,10.0) | 81.0 (25.9,177.7) | 8.5 (2.7,18.7) |

Impact of sub-optimal prevalences in low birthweight (LBW), preterm birth (PTB), or small for gestational age (SGA) on human capital and labor market outcomes in Solomon Islands.

^1^ TMRED = Theoretical Minimum Risk Exposure Distribution (TMRED)

^2^ Source: United National Population Division World Population Prospects 2019.

^3^ Country specific annual wage data from World Indicators Database. Average yearly wage was estimated to be 2/3 of the gross domestic product in 2010 constant US dollars and 2011 International dollars, adjusted for purchasing power parity.

**Somalia**

**Super region:** Sub-Saharan Africa**; Sub region:** Eastern Sub-Saharan Africa

**Number of live births^3^:** 3,108,000

**Probability of survival to age 25^2^**: 0.80

**GDP per capita 2010 US dollars (estimated annual wage)^3^:**  269

**GDP per capita 2011 International dollars (estimated annual wage)^3^:**  544

| **Birth outcome** | **Current**  **prevalence (%)** | **TMRED^1^ (%)** | **Total school years gained per birth cohort**  **(in 1000s)** | **Increase in lifetime earnings in USD per child (20 to 59yrs)** | **Benefits by cohorts (20 to 59yrs)** **Lifetime wages**  **(in USD millions)** |
| --- | --- | --- | --- | --- | --- |
| **LBW** | 11.2 (5.6,16.8) | 3.2 | 54.3 (7.6,121.4) | 9.6 (1.2,21.5) | 29.8 (3.7,66.7) |
| **PTB** | 12.0 (8.6,16.7) | 5.5 | 47.1 (5.8,115.1) | 8.3 (1.1,20.9) | 25.9 (3.3,64.8) |
| **SGA** | 25.1 (20.8,29.3) | 10 | 152.0 (71.2,247.4) | 26.8 (12.1,45.5) | 83.2 (37.7,141.4) |

Impact of sub-optimal prevalences in low birthweight (LBW), preterm birth (PTB), or small for gestational age (SGA) on human capital and labor market outcomes in Somalia.

^1^ TMRED = Theoretical Minimum Risk Exposure Distribution (TMRED)

^2^ Source: United National Population Division World Population Prospects 2019.

^3^ Country specific annual wage data from World Indicators Database. Average yearly wage was estimated to be 2/3 of the gross domestic product in 2010 constant US dollars and 2011 International dollars, adjusted for purchasing power parity.

**South Africa**

**Super region:** Sub-Saharan Africa**; Sub region:** Southern Sub-Saharan Africa

**Number of live births^3^:** 5,925,000

**Probability of survival to age 25^2^**: 0.92

**GDP per capita 2010 US dollars (estimated annual wage)^3^:**  7,557

**GDP per capita 2011 International dollars (estimated annual wage)^3^:**  12,346

| **Birth outcome** | **Current**  **prevalence (%)** | **TMRED^1^ (%)** | **Total school years gained per birth cohort**  **(in 1000s)** | **Increase in lifetime earnings in USD per child (20 to 59yrs)** | **Benefits by cohorts (20 to 59yrs)** **Lifetime wages**  **(in USD millions)** |
| --- | --- | --- | --- | --- | --- |
| **LBW** | 14.2 (11.1,18.6) | 3.2 | 168.6 (51.4,316.0) | 693.9 (200.3,1351.8) | 4111.4 (1187.0,8009.3) |
| **PTB** | 12.4 (8.6,17.1) | 5.5 | 113.9 (14.1,261.7) | 464.1 (61.6,1190.2) | 2749.6 (364.9,7051.9) |
| **SGA** | 23.0 (17.1,26.6) | 10 | 284.5 (127.3,495.5) | 1176.9 (483.0,2156.5) | 6973.0 (2861.7,12777.0) |

Impact of sub-optimal prevalences in low birthweight (LBW), preterm birth (PTB), or small for gestational age (SGA) on human capital and labor market outcomes in South Africa.

^1^ TMRED = Theoretical Minimum Risk Exposure Distribution (TMRED)

^2^ Source: United National Population Division World Population Prospects 2019.

^3^ Country specific annual wage data from World Indicators Database. Average yearly wage was estimated to be 2/3 of the gross domestic product in 2010 constant US dollars and 2011 International dollars, adjusted for purchasing power parity.

**South Sudan**

**Super region:** Sub-Saharan Africa**; Sub region:** Eastern Sub-Saharan Africa

**Number of live births^3^:** 1,928,000

**Probability of survival to age 25^2^**: 0.82

**GDP per capita 2010 US dollars (estimated annual wage)^3^:**  787

**GDP per capita 2011 International dollars (estimated annual wage)^3^:**  1,910

| **Birth outcome** | **Current**  **prevalence (%)** | **TMRED^1^ (%)** | **Total school years gained per birth cohort**  **(in 1000s)** | **Increase in lifetime earnings in USD per child (20 to 59yrs)** | **Benefits by cohorts (20 to 59yrs)** **Lifetime wages**  **(in USD millions)** |
| --- | --- | --- | --- | --- | --- |
| **LBW** | 15.4 (9.9,20.9) | 3.2 | 53.6 (13.3,106.7) | 44.6 (11.2,89.9) | 86.0 (21.5,173.3) |
| **PTB** | 12.0 (8.6,16.7) | 5.5 | 30.1 (2.7,72.9) | 25.2 (2.4,58.5) | 48.5 (4.6,112.9) |
| **SGA** | 25.1 (21.6,28.7) | 10 | 95.6 (44.7,159.2) | 80.1 (36.0,133.0) | 154.4 (69.5,256.5) |

Impact of sub-optimal prevalences in low birthweight (LBW), preterm birth (PTB), or small for gestational age (SGA) on human capital and labor market outcomes in South Sudan.

^1^ TMRED = Theoretical Minimum Risk Exposure Distribution (TMRED)

^2^ Source: United National Population Division World Population Prospects 2019.

^3^ Country specific annual wage data from World Indicators Database. Average yearly wage was estimated to be 2/3 of the gross domestic product in 2010 constant US dollars and 2011 International dollars, adjusted for purchasing power parity.

**Sri Lanka**

**Super region:** Southeast Asia, East Asia, and Oceania**; Sub region:** Southeast Asia

**Number of live births^3^:** 1,695,000

**Probability of survival to age 25^2^**: 0.98

**GDP per capita 2010 US dollars (estimated annual wage)^3^:**  3,647

**GDP per capita 2011 International dollars (estimated annual wage)^3^:**  11,078

| **Birth outcome** | **Current**  **prevalence (%)** | **TMRED^1^ (%)** | **Total school years gained per birth cohort**  **(in 1000s)** | **Increase in lifetime earnings in USD per child (20 to 59yrs)** | **Benefits by cohorts (20 to 59yrs)** **Lifetime wages**  **(in USD millions)** |
| --- | --- | --- | --- | --- | --- |
| **LBW** | 15.9 (15.6,16.1) | 3.2 | 60.8 (18.3,102.3) | 45.4 (13.8,77.8) | 76.9 (23.3,131.8) |
| **PTB** | 7.0 (4.2,10.7) | 5.5 | 7.1 (0.0,31.8) | 5.3 (0.0,23.7) | 9.0 (0.0,40.1) |
| **SGA** | 24.3 (18.7,30.8) | 10 | 94.2 (38.7,165.7) | 69.8 (29.8,126.4) | 118.4 (50.5,214.3) |

Impact of sub-optimal prevalences in low birthweight (LBW), preterm birth (PTB), or small for gestational age (SGA) on human capital and labor market outcomes in Sri Lanka.

^1^ TMRED = Theoretical Minimum Risk Exposure Distribution (TMRED)

^2^ Source: United National Population Division World Population Prospects 2019.

^3^ Country specific annual wage data from World Indicators Database. Average yearly wage was estimated to be 2/3 of the gross domestic product in 2010 constant US dollars and 2011 International dollars, adjusted for purchasing power parity.

**Saint Lucia**

**Super region:** Latin America and Caribbean**; Sub region:** Caribbean

**Number of live births^3^:** 11,000

**Probability of survival to age 25^2^**: 0.97

**GDP per capita 2010 US dollars (estimated annual wage)^3^:**  7,969

**GDP per capita 2011 International dollars (estimated annual wage)^3^:**  11,568

| **Birth outcome** | **Current**  **prevalence (%)** | **TMRED^1^ (%)** | **Total school years gained per birth cohort**  **(in 1000s)** | **Increase in lifetime earnings in USD per child (20 to 59yrs)** | **Benefits by cohorts (20 to 59yrs)** **Lifetime wages**  **(in USD millions)** |
| --- | --- | --- | --- | --- | --- |
| **LBW** | 10.1 (6.3,13.8) | 3.2 | 0.2 (0.1,0.4) | 258.1 (67.7,566.3) | 2.8 (0.7,6.2) |
| **PTB** | 9.8 (8.6,11.3) | 5.5 | 0.1 (0.0,0.3) | 179.1 (22.2,379.2) | 2.0 (0.2,4.2) |
| **SGA** | 15.4 (11.7,20.5) | 10 | 0.2 (0.0,0.5) | 296.9 (56.5,654.1) | 3.3 (0.6,7.2) |

Impact of sub-optimal prevalences in low birthweight (LBW), preterm birth (PTB), or small for gestational age (SGA) on human capital and labor market outcomes in Saint Lucia.

^1^ TMRED = Theoretical Minimum Risk Exposure Distribution (TMRED)

^2^ Source: United National Population Division World Population Prospects 2019.

^3^ Country specific annual wage data from World Indicators Database. Average yearly wage was estimated to be 2/3 of the gross domestic product in 2010 constant US dollars and 2011 International dollars, adjusted for purchasing power parity.

**Sudan**

**Super region:** Sub-Saharan Africa**; Sub region:** Eastern Sub-Saharan Africa

**Number of live births^3^:** 6,695,000

**Probability of survival to age 25^2^**: 0.88

**GDP per capita 2010 US dollars (estimated annual wage)^3^:**  1,870

**GDP per capita 2011 International dollars (estimated annual wage)^3^:**  4,262

| **Birth outcome** | **Current**  **prevalence (%)** | **TMRED^1^ (%)** | **Total school years gained per birth cohort**  **(in 1000s)** | **Increase in lifetime earnings in USD per child (20 to 59yrs)** | **Benefits by cohorts (20 to 59yrs)** **Lifetime wages**  **(in USD millions)** |
| --- | --- | --- | --- | --- | --- |
| **LBW** | 30.7 (25.1,36.3) | 3.2 | 466.9 (132.1,816.4) | 262.6 (74.1,472.5) | 1758.3 (496.0,3163.6) |
| **PTB** | 13.4 (6.3,30.9) | 5.5 | 124.0 (0.0,474.8) | 71.4 (0.0,284.1) | 477.8 (0.0,1902.1) |
| **SGA** | 41.7 (34.7,49.8) | 10 | 761.2 (347.9,1232.5) | 429.8 (204.0,708.2) | 2877.6 (1365.7,4741.5) |

Impact of sub-optimal prevalences in low birthweight (LBW), preterm birth (PTB), or small for gestational age (SGA) on human capital and labor market outcomes in Sudan.

^1^ TMRED = Theoretical Minimum Risk Exposure Distribution (TMRED)

^2^ Source: United National Population Division World Population Prospects 2019.

^3^ Country specific annual wage data from World Indicators Database. Average yearly wage was estimated to be 2/3 of the gross domestic product in 2010 constant US dollars and 2011 International dollars, adjusted for purchasing power parity.

**Suriname**

**Super region:** Latin America and Caribbean**; Sub region:** Caribbean

**Number of live births^3^:** 53,000

**Probability of survival to age 25^2^**: 0.96

**GDP per capita 2010 US dollars (estimated annual wage)^3^:**  8,465

**GDP per capita 2011 International dollars (estimated annual wage)^3^:**  14,488

| **Birth outcome** | **Current**  **prevalence (%)** | **TMRED^1^ (%)** | **Total school years gained per birth cohort**  **(in 1000s)** | **Increase in lifetime earnings in USD per child (20 to 59yrs)** | **Benefits by cohorts (20 to 59yrs)** **Lifetime wages**  **(in USD millions)** |
| --- | --- | --- | --- | --- | --- |
| **LBW** | 14.7 (11.4,18.7) | 3.2 | 1.7 (0.5,3.0) | 471.1 (128.5,861.2) | 25.0 (6.8,45.6) |
| **PTB** | 9.8 (8.6,11.3) | 5.5 | 0.7 (0.1,1.3) | 188.8 (27.1,385.8) | 10.0 (1.4,20.4) |
| **SGA** | 17.3 (13.2,21.8) | 10 | 1.4 (0.4,2.9) | 410.5 (119.6,812.8) | 21.8 (6.3,43.1) |

Impact of sub-optimal prevalences in low birthweight (LBW), preterm birth (PTB), or small for gestational age (SGA) on human capital and labor market outcomes in Suriname.

^1^ TMRED = Theoretical Minimum Risk Exposure Distribution (TMRED)

^2^ Source: United National Population Division World Population Prospects 2019.

^3^ Country specific annual wage data from World Indicators Database. Average yearly wage was estimated to be 2/3 of the gross domestic product in 2010 constant US dollars and 2011 International dollars, adjusted for purchasing power parity.

**Syria**

**Super region:** North Africa and Middle East**; Sub region:** North Africa and Middle East

**Number of live births^3^:** 2,130,000

**Probability of survival to age 25^2^**: 0.93

**GDP per capita 2010 US dollars (estimated annual wage)^3^:**  1,864

**GDP per capita 2011 International dollars (estimated annual wage)^3^:**  4,685

| **Birth outcome** | **Current**  **prevalence (%)** | **TMRED^1^ (%)** | **Total school years gained per birth cohort**  **(in 1000s)** | **Increase in lifetime earnings in USD per child (20 to 59yrs)** | **Benefits by cohorts (20 to 59yrs)** **Lifetime wages**  **(in USD millions)** |
| --- | --- | --- | --- | --- | --- |
| **LBW** | 9.4 (5.4,13.4) | 3.2 | 33.9 (6.2,74.8) | 34.6 (6.8,82.8) | 73.7 (14.5,176.3) |
| **PTB** | 10.4 (8.7,11.9) | 5.5 | 30.4 (4.3,62.0) | 30.7 (4.1,71.5) | 65.4 (8.8,152.4) |
| **SGA** | 19.0 (13.9,25.5) | 10 | 71.1 (19.2,152.0) | 70.7 (18.5,177.0) | 150.7 (39.4,377.1) |

Impact of sub-optimal prevalences in low birthweight (LBW), preterm birth (PTB), or small for gestational age (SGA) on human capital and labor market outcomes in Syria.

^1^ TMRED = Theoretical Minimum Risk Exposure Distribution (TMRED)

^2^ Source: United National Population Division World Population Prospects 2019.

^3^ Country specific annual wage data from World Indicators Database. Average yearly wage was estimated to be 2/3 of the gross domestic product in 2010 constant US dollars and 2011 International dollars, adjusted for purchasing power parity.

**Tajikistan**

**Super region:** Central Europe, Eastern Europe, Central Asia**; Sub region:** Central Asia

**Number of live births^3^:** 1,404,000

**Probability of survival to age 25^2^**: 0.96

**GDP per capita 2010 US dollars (estimated annual wage)^3^:**  936

**GDP per capita 2011 International dollars (estimated annual wage)^3^:**  2,670

| **Birth outcome** | **Current**  **prevalence (%)** | **TMRED^1^ (%)** | **Total school years gained per birth cohort**  **(in 1000s)** | **Increase in lifetime earnings in USD per child (20 to 59yrs)** | **Benefits by cohorts (20 to 59yrs)** **Lifetime wages**  **(in USD millions)** |
| --- | --- | --- | --- | --- | --- |
| **LBW** | 5.6 (4.4,7.3) | 3.2 | 9.1 (2.0,18.2) | 3.1 (0.3,8.8) | 4.4 (0.4,12.3) |
| **PTB** | 10.4 (8.7,11.9) | 5.5 | 19.9 (2.7,41.0) | 7.4 (0.6,19.5) | 10.4 (0.8,27.3) |
| **SGA** | 17.0 (12.1,23.9) | 10 | 36.9 (4.0,84.3) | 13.1 (0.6,39.8) | 18.4 (0.9,55.8) |

Impact of sub-optimal prevalences in low birthweight (LBW), preterm birth (PTB), or small for gestational age (SGA) on human capital and labor market outcomes in Tajikistan.

^1^ TMRED = Theoretical Minimum Risk Exposure Distribution (TMRED)

^2^ Source: United National Population Division World Population Prospects 2019.

^3^ Country specific annual wage data from World Indicators Database. Average yearly wage was estimated to be 2/3 of the gross domestic product in 2010 constant US dollars and 2011 International dollars, adjusted for purchasing power parity.

**Tanzania**

**Super region:** Sub-Saharan Africa**; Sub region:** Eastern Sub-Saharan Africa

**Number of live births^3^:** 10,260,000

**Probability of survival to age 25^2^**: 0.90

**GDP per capita 2010 US dollars (estimated annual wage)^3^:**  872

**GDP per capita 2011 International dollars (estimated annual wage)^3^:**  2,613

| **Birth outcome** | **Current**  **prevalence (%)** | **TMRED^1^ (%)** | **Total school years gained per birth cohort**  **(in 1000s)** | **Increase in lifetime earnings in USD per child (20 to 59yrs)** | **Benefits by cohorts (20 to 59yrs)** **Lifetime wages**  **(in USD millions)** |
| --- | --- | --- | --- | --- | --- |
| **LBW** | 10.5 (8.1,13.4) | 3.2 | 190.6 (52.0,360.2) | 23.6 (6.2,49.1) | 242.4 (63.9,503.8) |
| **PTB** | 16.6 (6.5,33.4) | 5.5 | 287.2 (0.0,981.4) | 35.9 (0.0,132.9) | 368.3 (0.0,1363.7) |
| **SGA** | 17.8 (14.1,21.3) | 10 | 286.9 (115.7,529.7) | 35.8 (14.0,70.8) | 366.8 (143.2,726.0) |

Impact of sub-optimal prevalences in low birthweight (LBW), preterm birth (PTB), or small for gestational age (SGA) on human capital and labor market outcomes in Tanzania.

^1^ TMRED = Theoretical Minimum Risk Exposure Distribution (TMRED)

^2^ Source: United National Population Division World Population Prospects 2019.

^3^ Country specific annual wage data from World Indicators Database. Average yearly wage was estimated to be 2/3 of the gross domestic product in 2010 constant US dollars and 2011 International dollars, adjusted for purchasing power parity.

**Thailand**

**Super region:** Southeast Asia, East Asia, and Oceania**; Sub region:** Southeast Asia

**Number of live births^3^:** 3,623,000

**Probability of survival to age 25^2^**: 0.97

**GDP per capita 2010 US dollars (estimated annual wage)^3^:**  5,741

**GDP per capita 2011 International dollars (estimated annual wage)^3^:**  15,256

| **Birth outcome** | **Current**  **prevalence (%)** | **TMRED^1^ (%)** | **Total school years gained per birth cohort**  **(in 1000s)** | **Increase in lifetime earnings in USD per child (20 to 59yrs)** | **Benefits by cohorts (20 to 59yrs)** **Lifetime wages**  **(in USD millions)** |
| --- | --- | --- | --- | --- | --- |
| **LBW** | 10.5 (10.3,10.8) | 3.2 | 74.6 (22.4,125.3) | 233.5 (69.0,397.8) | 845.9 (250.1,1441.2) |
| **PTB** | 12.7 (10.1,15.6) | 5.5 | 78.4 (13.1,164.0) | 244.1 (42.2,516.9) | 884.5 (153.0,1872.9) |
| **SGA** | 18.8 (13.7,25.2) | 10 | 120.5 (39.3,240.7) | 377.8 (120.3,756.3) | 1368.8 (436.0,2740.1) |

Impact of sub-optimal prevalences in low birthweight (LBW), preterm birth (PTB), or small for gestational age (SGA) on human capital and labor market outcomes in Thailand.

^1^ TMRED = Theoretical Minimum Risk Exposure Distribution (TMRED)

^2^ Source: United National Population Division World Population Prospects 2019.

^3^ Country specific annual wage data from World Indicators Database. Average yearly wage was estimated to be 2/3 of the gross domestic product in 2010 constant US dollars and 2011 International dollars, adjusted for purchasing power parity.

**Timor-Leste**

**Super region:** Southeast Asia, East Asia, and Oceania**; Sub region:** Southeast Asia

**Number of live births^3^:** 187,000

**Probability of survival to age 25^2^**: 0.93

**GDP per capita 2010 US dollars (estimated annual wage)^3^:**  3,109

**GDP per capita 2011 International dollars (estimated annual wage)^3^:**  7,657

| **Birth outcome** | **Current**  **prevalence (%)** | **TMRED^1^ (%)** | **Total school years gained per birth cohort**  **(in 1000s)** | **Increase in lifetime earnings in USD per child (20 to 59yrs)** | **Benefits by cohorts (20 to 59yrs)** **Lifetime wages**  **(in USD millions)** |
| --- | --- | --- | --- | --- | --- |
| **LBW** | 12.0 (6.0,17.9) | 3.2 | 4.1 (0.7,9.2) | 53.6 (4.7,170.3) | 10.0 (0.9,31.8) |
| **PTB** | 10.4 (8.7,11.9) | 5.5 | 2.6 (0.4,5.3) | 33.4 (1.9,102.4) | 6.2 (0.4,19.1) |
| **SGA** | 26.3 (21.2,32.8) | 10 | 11.4 (5.0,19.1) | 151.7 (27.6,366.6) | 28.4 (5.2,68.6) |

Impact of sub-optimal prevalences in low birthweight (LBW), preterm birth (PTB), or small for gestational age (SGA) on human capital and labor market outcomes in Timor-Leste.

^1^ TMRED = Theoretical Minimum Risk Exposure Distribution (TMRED)

^2^ Source: United National Population Division World Population Prospects 2019.

^3^ Country specific annual wage data from World Indicators Database. Average yearly wage was estimated to be 2/3 of the gross domestic product in 2010 constant US dollars and 2011 International dollars, adjusted for purchasing power parity.

**Togo**

**Super region:** Sub-Saharan Africa**; Sub region:** Western Sub-Saharan Africa

**Number of live births^3^:** 1,299,000

**Probability of survival to age 25^2^**: 0.85

**GDP per capita 2010 US dollars (estimated annual wage)^3^:**  630

**GDP per capita 2011 International dollars (estimated annual wage)^3^:**  1,467

| **Birth outcome** | **Current**  **prevalence (%)** | **TMRED^1^ (%)** | **Total school years gained per birth cohort**  **(in 1000s)** | **Increase in lifetime earnings in USD per child (20 to 59yrs)** | **Benefits by cohorts (20 to 59yrs)** **Lifetime wages**  **(in USD millions)** |
| --- | --- | --- | --- | --- | --- |
| **LBW** | 16.1 (12.8,20.3) | 3.2 | 40.5 (11.4,73.3) | 21.4 (6.0,43.7) | 27.8 (7.8,56.7) |
| **PTB** | 12.0 (8.6,16.7) | 5.5 | 21.5 (2.5,48.9) | 11.5 (1.5,27.9) | 15.0 (2.0,36.2) |
| **SGA** | 22.8 (19.2,26.5) | 10 | 57.3 (26.7,94.5) | 30.8 (13.5,53.7) | 40.0 (17.6,69.8) |

Impact of sub-optimal prevalences in low birthweight (LBW), preterm birth (PTB), or small for gestational age (SGA) on human capital and labor market outcomes in Togo.

^1^ TMRED = Theoretical Minimum Risk Exposure Distribution (TMRED)

^2^ Source: United National Population Division World Population Prospects 2019.

^3^ Country specific annual wage data from World Indicators Database. Average yearly wage was estimated to be 2/3 of the gross domestic product in 2010 constant US dollars and 2011 International dollars, adjusted for purchasing power parity.

**Tonga**

**Super region:** Southeast Asia, East Asia, and Oceania**; Sub region:** Oceania

**Number of live births^3^:** 13,000

**Probability of survival to age 25^2^**: 0.96

**GDP per capita 2010 US dollars (estimated annual wage)^3^:**  3,899

**GDP per capita 2011 International dollars (estimated annual wage)^3^:**  5,477

| **Birth outcome** | **Current**  **prevalence (%)** | **TMRED^1^ (%)** | **Total school years gained per birth cohort**  **(in 1000s)** | **Increase in lifetime earnings in USD per child (20 to 59yrs)** | **Benefits by cohorts (20 to 59yrs)** **Lifetime wages**  **(in USD millions)** |
| --- | --- | --- | --- | --- | --- |
| **LBW** | 3.0 (0.3,5.7) | 3.2 | 0.0 (0.0,0.1) | 0.0 (0.0,33.6) | 0.0 (0.0,0.4) |
| **PTB** | 10.0 (7.9,12.7) | 5.5 | 0.2 (0.0,0.4) | 53.5 (6.3,143.6) | 0.7 (0.1,1.9) |
| **SGA** | 7.5 (4.4,12.6) | 10 | 0.0 (0.0,0.1) | 0.0 (0.0,22.9) | 0.0 (0.0,0.3) |

Impact of sub-optimal prevalences in low birthweight (LBW), preterm birth (PTB), or small for gestational age (SGA) on human capital and labor market outcomes in Tonga.

^1^ TMRED = Theoretical Minimum Risk Exposure Distribution (TMRED)

^2^ Source: United National Population Division World Population Prospects 2019.

^3^ Country specific annual wage data from World Indicators Database. Average yearly wage was estimated to be 2/3 of the gross domestic product in 2010 constant US dollars and 2011 International dollars, adjusted for purchasing power parity.

**Tunisia**

**Super region:** North Africa and Middle East**; Sub region:** North Africa and Middle East

**Number of live births^3^:** 1,018,000

**Probability of survival to age 25^2^**: 0.98

**GDP per capita 2010 US dollars (estimated annual wage)^3^:**  4,308

**GDP per capita 2011 International dollars (estimated annual wage)^3^:**  10,861

| **Birth outcome** | **Current**  **prevalence (%)** | **TMRED^1^ (%)** | **Total school years gained per birth cohort**  **(in 1000s)** | **Increase in lifetime earnings in USD per child (20 to 59yrs)** | **Benefits by cohorts (20 to 59yrs)** **Lifetime wages**  **(in USD millions)** |
| --- | --- | --- | --- | --- | --- |
| **LBW** | 7.5 (5.9,9.9) | 3.2 | 11.9 (3.3,23.7) | 59.5 (14.5,135.5) | 60.6 (14.8,138.0) |
| **PTB** | 13.4 (6.3,30.9) | 5.5 | 20.8 (0.0,83.8) | 104.6 (0.0,469.7) | 106.5 (0.0,478.1) |
| **SGA** | 9.6 (6.6,13.2) | 10 | 0.0 (0.0,12.9) | 0.0 (0.0,69.5) | 0.0 (0.0,70.7) |

Impact of sub-optimal prevalences in low birthweight (LBW), preterm birth (PTB), or small for gestational age (SGA) on human capital and labor market outcomes in Tunisia.

^1^ TMRED = Theoretical Minimum Risk Exposure Distribution (TMRED)

^2^ Source: United National Population Division World Population Prospects 2019.

^3^ Country specific annual wage data from World Indicators Database. Average yearly wage was estimated to be 2/3 of the gross domestic product in 2010 constant US dollars and 2011 International dollars, adjusted for purchasing power parity.

**Turkey**

**Super region:** North Africa and Middle East**; Sub region:** North Africa and Middle East

**Number of live births^3^:** 6,588,000

**Probability of survival to age 25^2^**: 0.97

**GDP per capita 2010 US dollars (estimated annual wage)^3^:**  13,853

**GDP per capita 2011 International dollars (estimated annual wage)^3^:**  23,312

| **Birth outcome** | **Current**  **prevalence (%)** | **TMRED^1^ (%)** | **Total school years gained per birth cohort**  **(in 1000s)** | **Increase in lifetime earnings in USD per child (20 to 59yrs)** | **Benefits by cohorts (20 to 59yrs)** **Lifetime wages**  **(in USD millions)** |
| --- | --- | --- | --- | --- | --- |
| **LBW** | 11.4 (9.0,14.5) | 3.2 | 148.2 (39.9,265.9) | 477.9 (129.7,878.0) | 3148.5 (854.5,5784.0) |
| **PTB** | 12.4 (8.7,17.0) | 5.5 | 127.3 (16.5,313.2) | 413.7 (47.3,1029.6) | 2725.8 (311.6,6783.1) |
| **SGA** | 21.7 (16.4,28.1) | 10 | 295.4 (109.1,565.8) | 931.4 (342.7,1917.5) | 6136.4 (2257.8,12632.5) |

Impact of sub-optimal prevalences in low birthweight (LBW), preterm birth (PTB), or small for gestational age (SGA) on human capital and labor market outcomes in Turkey.

^1^ TMRED = Theoretical Minimum Risk Exposure Distribution (TMRED)

^2^ Source: United National Population Division World Population Prospects 2019.

^3^ Country specific annual wage data from World Indicators Database. Average yearly wage was estimated to be 2/3 of the gross domestic product in 2010 constant US dollars and 2011 International dollars, adjusted for purchasing power parity.

**Turkmenistan**

**Super region:** Central Europe, Eastern Europe, Central Asia**; Sub region:** Central Asia

**Number of live births^3^:** 695,000

**Probability of survival to age 25^2^**: 0.93

**GDP per capita 2010 US dollars (estimated annual wage)^3^:**  6,694

**GDP per capita 2011 International dollars (estimated annual wage)^3^:**  14,992

| **Birth outcome** | **Current**  **prevalence (%)** | **TMRED^1^ (%)** | **Total school years gained per birth cohort**  **(in 1000s)** | **Increase in lifetime earnings in USD per child (20 to 59yrs)** | **Benefits by cohorts (20 to 59yrs)** **Lifetime wages**  **(in USD millions)** |
| --- | --- | --- | --- | --- | --- |
| **LBW** | 4.9 (3.8,6.2) | 3.2 | 2.9 (0.5,6.7) | 24.2 (3.2,64.7) | 16.8 (2.2,45.0) |
| **PTB** | 10.4 (8.7,11.9) | 5.5 | 9.6 (1.4,19.5) | 81.9 (10.9,202.3) | 56.9 (7.6,140.6) |
| **SGA** | 13.5 (8.9,19.9) | 10 | 9.1 (0.0,27.0) | 74.4 (0.0,276.6) | 51.7 (0.0,192.2) |

Impact of sub-optimal prevalences in low birthweight (LBW), preterm birth (PTB), or small for gestational age (SGA) on human capital and labor market outcomes in Turkmenistan.

^1^ TMRED = Theoretical Minimum Risk Exposure Distribution (TMRED)

^2^ Source: United National Population Division World Population Prospects 2019.

^3^ Country specific annual wage data from World Indicators Database. Average yearly wage was estimated to be 2/3 of the gross domestic product in 2010 constant US dollars and 2011 International dollars, adjusted for purchasing power parity.

**Uganda**

**Super region:** Sub-Saharan Africa**; Sub region:** Eastern Sub-Saharan Africa

**Number of live births^3^:** 8,069,000

**Probability of survival to age 25^2^**: 0.89

**GDP per capita 2010 US dollars (estimated annual wage)^3^:**  687

**GDP per capita 2011 International dollars (estimated annual wage)^3^:**  1,750

| **Birth outcome** | **Current**  **prevalence (%)** | **TMRED^1^ (%)** | **Total school years gained per birth cohort**  **(in 1000s)** | **Increase in lifetime earnings in USD per child (20 to 59yrs)** | **Benefits by cohorts (20 to 59yrs)** **Lifetime wages**  **(in USD millions)** |
| --- | --- | --- | --- | --- | --- |
| **LBW** | 9.3 (3.7,14.9) | 3.2 | 115.4 (0.0,290.3) | 21.0 (0.0,53.2) | 169.7 (0.0,429.2) |
| **PTB** | 6.6 (4.1,10.0) | 5.5 | 20.7 (0.0,105.8) | 3.8 (0.0,19.5) | 30.4 (0.0,157.4) |
| **SGA** | 24.5 (20.3,28.7) | 10 | 422.5 (177.7,688.9) | 77.1 (33.0,126.2) | 622.5 (266.4,1018.1) |

Impact of sub-optimal prevalences in low birthweight (LBW), preterm birth (PTB), or small for gestational age (SGA) on human capital and labor market outcomes in Uganda.

^1^ TMRED = Theoretical Minimum Risk Exposure Distribution (TMRED)

^2^ Source: United National Population Division World Population Prospects 2019.

^3^ Country specific annual wage data from World Indicators Database. Average yearly wage was estimated to be 2/3 of the gross domestic product in 2010 constant US dollars and 2011 International dollars, adjusted for purchasing power parity.

**Uzbekistan**

**Super region:** Central Europe, Eastern Europe, Central Asia**; Sub region:** Central Asia

**Number of live births^3^:** 3,513,000

**Probability of survival to age 25^2^**: 0.96

**GDP per capita 2010 US dollars (estimated annual wage)^3^:**  1,831

**GDP per capita 2011 International dollars (estimated annual wage)^3^:**  5,639

| **Birth outcome** | **Current**  **prevalence (%)** | **TMRED^1^ (%)** | **Total school years gained per birth cohort**  **(in 1000s)** | **Increase in lifetime earnings in USD per child (20 to 59yrs)** | **Benefits by cohorts (20 to 59yrs)** **Lifetime wages**  **(in USD millions)** |
| --- | --- | --- | --- | --- | --- |
| **LBW** | 5.3 (4.1,6.7) | 3.2 | 19.3 (4.0,43.9) | 8.8 (1.6,25.2) | 31.1 (5.6,88.6) |
| **PTB** | 10.4 (8.7,11.9) | 5.5 | 50.7 (7.8,103.9) | 23.0 (2.7,59.0) | 80.8 (9.4,207.3) |
| **SGA** | 12.2 (8.0,18.2) | 10 | 28.6 (0.0,116.8) | 12.7 (0.0,61.1) | 44.8 (0.0,214.6) |

Impact of sub-optimal prevalences in low birthweight (LBW), preterm birth (PTB), or small for gestational age (SGA) on human capital and labor market outcomes in Uzbekistan.

^1^ TMRED = Theoretical Minimum Risk Exposure Distribution (TMRED)

^2^ Source: United National Population Division World Population Prospects 2019.

^3^ Country specific annual wage data from World Indicators Database. Average yearly wage was estimated to be 2/3 of the gross domestic product in 2010 constant US dollars and 2011 International dollars, adjusted for purchasing power parity.

**Vanuatu**

**Super region:** Southeast Asia, East Asia, and Oceania**; Sub region:** Oceania

**Number of live births^3^:** 43,000

**Probability of survival to age 25^2^**: 0.96

**GDP per capita 2010 US dollars (estimated annual wage)^3^:**  2,756

**GDP per capita 2011 International dollars (estimated annual wage)^3^:**  2,739

| **Birth outcome** | **Current**  **prevalence (%)** | **TMRED^1^ (%)** | **Total school years gained per birth cohort**  **(in 1000s)** | **Increase in lifetime earnings in USD per child (20 to 59yrs)** | **Benefits by cohorts (20 to 59yrs)** **Lifetime wages**  **(in USD millions)** |
| --- | --- | --- | --- | --- | --- |
| **LBW** | 10.9 (8.6,13.9) | 3.2 | 0.9 (0.3,1.7) | 57.9 (13.5,136.2) | 2.5 (0.6,5.9) |
| **PTB** | 10.0 (7.9,12.7) | 5.5 | 0.5 (0.1,1.2) | 36.2 (3.7,98.8) | 1.6 (0.2,4.2) |
| **SGA** | 20.2 (14.8,27.0) | 10 | 1.6 (0.6,3.4) | 109.6 (30.6,275.6) | 4.7 (1.3,11.8) |

Impact of sub-optimal prevalences in low birthweight (LBW), preterm birth (PTB), or small for gestational age (SGA) on human capital and labor market outcomes in Vanuatu.

^1^ TMRED = Theoretical Minimum Risk Exposure Distribution (TMRED)

^2^ Source: United National Population Division World Population Prospects 2019.

^3^ Country specific annual wage data from World Indicators Database. Average yearly wage was estimated to be 2/3 of the gross domestic product in 2010 constant US dollars and 2011 International dollars, adjusted for purchasing power parity.

**Venezuela**

**Super region:** Latin America and Caribbean**; Sub region:** Central Latin America

**Number of live births^3^:** 2,638,000

**Probability of survival to age 25^2^**: 0.94

**GDP per capita 2010 US dollars (estimated annual wage)^3^:**  13,825

**GDP per capita 2011 International dollars (estimated annual wage)^3^:**  16,887

| **Birth outcome** | **Current**  **prevalence (%)** | **TMRED^1^ (%)** | **Total school years gained per birth cohort**  **(in 1000s)** | **Increase in lifetime earnings in USD per child (20 to 59yrs)** | **Benefits by cohorts (20 to 59yrs)** **Lifetime wages**  **(in USD millions)** |
| --- | --- | --- | --- | --- | --- |
| **LBW** | 9.1 (8.9,9.3) | 3.2 | 42.4 (12.4,71.3) | 342.8 (92.7,568.1) | 904.4 (244.4,1498.6) |
| **PTB** | 9.7 (7.7,12.1) | 5.5 | 30.6 (3.9,71.2) | 243.1 (33.4,569.4) | 641.3 (88.2,1502.1) |
| **SGA** | 12.9 (11.3,19.7) | 10 | 28.4 (0.0,85.3) | 225.7 (0.0,691.7) | 595.5 (0.0,1824.8) |

Impact of sub-optimal prevalences in low birthweight (LBW), preterm birth (PTB), or small for gestational age (SGA) on human capital and labor market outcomes in Venezuela.

^1^ TMRED = Theoretical Minimum Risk Exposure Distribution (TMRED)

^2^ Source: United National Population Division World Population Prospects 2019.

^3^ Country specific annual wage data from World Indicators Database. Average yearly wage was estimated to be 2/3 of the gross domestic product in 2010 constant US dollars and 2011 International dollars, adjusted for purchasing power parity.

**Vietnam**

**Super region:** Southeast Asia, East Asia, and Oceania**; Sub region:** Southeast Asia

**Number of live births^3^:** 8,050,000

**Probability of survival to age 25^2^**: 0.96

**GDP per capita 2010 US dollars (estimated annual wage)^3^:**  1,667

**GDP per capita 2011 International dollars (estimated annual wage)^3^:**  5,608

| **Birth outcome** | **Current**  **prevalence (%)** | **TMRED^1^ (%)** | **Total school years gained per birth cohort**  **(in 1000s)** | **Increase in lifetime earnings in USD per child (20 to 59yrs)** | **Benefits by cohorts (20 to 59yrs)** **Lifetime wages**  **(in USD millions)** |
| --- | --- | --- | --- | --- | --- |
| **LBW** | 8.2 (5.8,10.6) | 3.2 | 105.2 (27.4,213.9) | 18.7 (4.8,41.4) | 150.7 (39.0,332.9) |
| **PTB** | 6.5 (4.3,9.3) | 5.5 | 22.4 (0.0,103.0) | 3.9 (0.0,19.0) | 31.8 (0.0,152.6) |
| **SGA** | 12.6 (8.2,18.0) | 10 | 76.6 (0.0,257.0) | 13.9 (0.0,47.7) | 112.0 (0.0,384.4) |

Impact of sub-optimal prevalences in low birthweight (LBW), preterm birth (PTB), or small for gestational age (SGA) on human capital and labor market outcomes in Vietnam.

^1^ TMRED = Theoretical Minimum Risk Exposure Distribution (TMRED)

^2^ Source: United National Population Division World Population Prospects 2019.

^3^ Country specific annual wage data from World Indicators Database. Average yearly wage was estimated to be 2/3 of the gross domestic product in 2010 constant US dollars and 2011 International dollars, adjusted for purchasing power parity.

**Yemen**

**Super region:** North Africa and Middle East**; Sub region:** North Africa and Middle East

**Number of live births^3^:** 4,324,000

**Probability of survival to age 25^2^**: 0.91

**GDP per capita 2010 US dollars (estimated annual wage)^3^:**  909

**GDP per capita 2011 International dollars (estimated annual wage)^3^:**  3,109

| **Birth outcome** | **Current**  **prevalence (%)** | **TMRED^1^ (%)** | **Total school years gained per birth cohort**  **(in 1000s)** | **Increase in lifetime earnings in USD per child (20 to 59yrs)** | **Benefits by cohorts (20 to 59yrs)** **Lifetime wages**  **(in USD millions)** |
| --- | --- | --- | --- | --- | --- |
| **LBW** | 23.0 (19.0,27.0) | 3.2 | 223.8 (67.0,388.5) | 55.2 (14.7,110.7) | 238.5 (63.7,478.7) |
| **PTB** | 10.4 (8.7,11.9) | 5.5 | 59.4 (8.8,122.7) | 14.6 (2.2,33.8) | 63.1 (9.3,146.2) |
| **SGA** | 42.3 (37.6,48.3) | 10 | 514.9 (247.4,810.0) | 128.3 (52.3,234.6) | 554.7 (226.2,1014.2) |

Impact of sub-optimal prevalences in low birthweight (LBW), preterm birth (PTB), or small for gestational age (SGA) on human capital and labor market outcomes in Yemen.

^1^ TMRED = Theoretical Minimum Risk Exposure Distribution (TMRED)

^2^ Source: United National Population Division World Population Prospects 2019.

^3^ Country specific annual wage data from World Indicators Database. Average yearly wage was estimated to be 2/3 of the gross domestic product in 2010 constant US dollars and 2011 International dollars, adjusted for purchasing power parity.

**Zambia**

**Super region:** Sub-Saharan Africa**; Sub region:** Eastern Sub-Saharan Africa

**Number of live births^3^:** 3,108,000

**Probability of survival to age 25^2^**: 0.90

**GDP per capita 2010 US dollars (estimated annual wage)^3^:**  1,641

**GDP per capita 2011 International dollars (estimated annual wage)^3^:**  3,678

| **Birth outcome** | **Current**  **prevalence (%)** | **TMRED^1^ (%)** | **Total school years gained per birth cohort**  **(in 1000s)** | **Increase in lifetime earnings in USD per child (20 to 59yrs)** | **Benefits by cohorts (20 to 59yrs)** **Lifetime wages**  **(in USD millions)** |
| --- | --- | --- | --- | --- | --- |
| **LBW** | 11.6 (9.2,14.8) | 3.2 | 66.3 (17.5,122.7) | 70.9 (18.9,132.4) | 220.2 (58.7,411.4) |
| **PTB** | 12.0 (8.6,16.7) | 5.5 | 52.5 (6.8,133.1) | 56.7 (7.1,145.9) | 176.3 (22.0,453.5) |
| **SGA** | 20.5 (16.9,24.6) | 10 | 118.8 (51.5,207.9) | 125.9 (52.3,224.0) | 391.2 (162.4,696.1) |

Impact of sub-optimal prevalences in low birthweight (LBW), preterm birth (PTB), or small for gestational age (SGA) on human capital and labor market outcomes in Zambia.

^1^ TMRED = Theoretical Minimum Risk Exposure Distribution (TMRED)

^2^ Source: United National Population Division World Population Prospects 2019.

^3^ Country specific annual wage data from World Indicators Database. Average yearly wage was estimated to be 2/3 of the gross domestic product in 2010 constant US dollars and 2011 International dollars, adjusted for purchasing power parity.

**Zimbabwe**

**Super region:** Sub-Saharan Africa**; Sub region:** Southern Sub-Saharan Africa

**Number of live births^3^:** 2,211,000

**Probability of survival to age 25^2^**: 0.90

**GDP per capita 2010 US dollars (estimated annual wage)^3^:**  1,234

**GDP per capita 2011 International dollars (estimated annual wage)^3^:**  2,509

| **Birth outcome** | **Current**  **prevalence (%)** | **TMRED^1^ (%)** | **Total school years gained per birth cohort**  **(in 1000s)** | **Increase in lifetime earnings in USD per child (20 to 59yrs)** | **Benefits by cohorts (20 to 59yrs)** **Lifetime wages**  **(in USD millions)** |
| --- | --- | --- | --- | --- | --- |
| **LBW** | 12.6 (10.0,16.5) | 3.2 | 51.2 (15.0,98.9) | 82.3 (24.1,159.1) | 181.9 (53.4,351.8) |
| **PTB** | 12.0 (8.6,16.7) | 5.5 | 38.7 (5.2,88.5) | 62.0 (8.5,141.8) | 137.1 (18.8,313.6) |
| **SGA** | 22.0 (18.3,25.4) | 10 | 95.1 (41.9,160.2) | 153.3 (67.1,262.4) | 338.9 (148.4,580.1) |

Impact of sub-optimal prevalences in low birthweight (LBW), preterm birth (PTB), or small for gestational age (SGA) on human capital and labor market outcomes in Zimbabwe.

^1^ TMRED = Theoretical Minimum Risk Exposure Distribution (TMRED)

^2^ Source: United National Population Division World Population Prospects 2019.

^3^ Country specific annual wage data from World Indicators Database. Average yearly wage was estimated to be 2/3 of the gross domestic product in 2010 constant US dollars and 2011 International dollars, adjusted for purchasing power parity.
