# Supplementary material for: Large gains in schooling and income are possible from minimizing adverse birth outcomes in 121 low- and middle-income countries: A modelling study
Source: PLOS Glob Public Health. 2022 Jun 8;2(6):e0000218. doi: 10.1371/journal.pgph.0000218 (PMC10021521; doi:10.1371/journal.pgph.0000218)
Supplement: Attachment — Submitted filename: TMRED - PLoS GPH - Response to reviewers 21jan22.docx [file pgph.0000218.s005.docx]

**Response to Editor and Reviewer comments:**

**Editor Comments:**

**Thank you for submitting your paper to PGPH for publication. The reviewers felt it presents an interesting piece of work. However, they raised a number of issues (attached) that will need your attention before the paper can be published in PGBH.**

**Further, after going through the same, I have also identified the under-listed issues for your consideration:**

**1. Lines 81-84: A bit more detail explanation of the frameworks will be helpful to the reader---the paper should be self-containing**

Response: Thank you for the review of our manuscript and for consideration for publication after revision. We have added the following to the methods section:

“We used a population attributable risk framework to estimate the potential human capital gains in school years and lifetime income for reducing the excess prevalence of LBW, SGA, PTB from their 2015 national prevalence in 121 LMICs to the theoretical minimum prevalence levels. 13 We used a framework previously conceptualized to estimate the potential human capital benefits of scaling up prenatal nutrition interventions for schooling and lifetime income through reductions in adverse birth outcomes mediators in the pathway (Perumal et al., 2021). While in previous work we have sought to estimate the effect of nutrition interventions with convincing level of evidence for a beneficial effect on reducing the risk of low birthweight or preterm birth, in this study we applied a theoretical approach to estimate the potential benefits of reducing the prevalence of adverse birth outcomes to the minimum prevalence observed in a population of healthy pregnancies.”

**2. Lines 107, 112-118, and 151, need to be referenced**

Response: References have been added for each line according to the request.

**3. Take out the ORs and CIs completely from the discussion section. This section is not meant for the restatement of the results, but to discuss what the results mean, and in the context of the literature.**

Response: We have removed ORs and CIs from the discussion section.

**4. The discussion section needs to be thoroughly revised---in some places it sound more like the restatement of results than discussion**

Response: We have thoroughly revised the discussion section. We now include a first paragraph of the discussion section that serves as a summary of the results. We have revised the subsequent paragraphs of the discussion to include a more detailed comparison to the extant literature and the methods used.

**5. What the results mean and their connection with the extant literature did not come out strongly. As an example, the connection between the findings of your study and the existing literature in lines 260-271 is not clear.**

Response: We have revised the discussion section to make a clearer connection between our findings and the existing literature.

Line 360: “Our findings add to a very limited literature on the potential long-term economic returns of reducing birth outcomes and early life exposures and adversities in LMICs.11,22 To our knowledge, only one other study has looked at the potential economic gains of reducing the prevalence of LBW: Alderman and Behrman estimated that relative to infants born LBW, non-LBW infants may have a greater lifetime earning potential of US $832 (at 3% discounting).11 Alderman and Behrman uses slightly different methods and investigate a wider range of potential gains (including not only cognitive delay, but also reduced costs from infant mortality, neonatal care, sickness, stunting, and chronic diseases). Of that, US $367 were estimated as productivity gains from increased intelligence quotients and schooling attainment, in the absence of LBW.11 In comparison, we estimated a global average potential gain of US$ 717 per LBW case averted. Our results suggest that the returns on wage gains from increased schooling may be almost twice that estimated in their analysis. If returns on investment in reducing adverse birth outcomes like LBW are substantially higher than previously estimated, this analysis may serve as a stronger advocacy tool for increasing investments in reducing the occurrence of LBW pregnancies.

We report the first estimates the potential education and lifetime earnings gains to reducing PTB and SGA It is important to note that the large gains in sub-Saharan Africa and South Asia can in part be attributed to the higher prevalence of PTB and SGA in these regions. This is particularly important to keep in mind when looking at the high returns for SGA in South Asia, where the median prevalence of SGA born babies was 40%. Several studies have found significant associations between prematurity, LBW and SGA with cognitive performance of school-aged children.”

**6. I was expecting to see a bit of discussion on the analytical strategies used in the context of the results (e.g. superior findings, or otherwise, as a result of employing the same)**

Response: Thank you for bringing this to our attention. Our revised discussion of the extant literature and the results and limitation section have attempted to expand on the discussion of analytical strategies used both by our study and those of the extant literature. We have also added more explicit discussion of some of the analytical strategies used in the following paragraph:

Line 415: “Our analytical strategy included analyses based on not just LBW, but also PTB and SGA, and enables a comparison of potential impacts across each type of adverse birth outcome. This was done because LBW does not differentiate between prematurity and intrauterine growth restriction and each mechanism may have different long-term effects on schooling and life income. The inclusion of PTB and SGA is an important addition to the limited literature on potential long-term human capital gains of reducing adverse birth outcomes. It is also important to note that LBW, PTB, and SGA can be coexisting conditions; therefore, the impacts of reducing each on schooling and income should be interpreted independently rather than cumulatively. We were not able to calculate the joint effect of the three outcomes due to lack of country-level estimates of their joint distribution. Nevertheless, the joint effect would be larger than each individual effect, but smaller than the sum of all three. Therefore, the impacts of each birth outcome should be interpreted independent of the other adverse birth outcomes.”

**7. Data quality issues (quality of studies etc) have not been discussed.**

Response: Thank you for bringing this to our attention. We have added the following to the discussion (in the strengths and limitation paragraph):

Line 513: “Our estimates are limited by the availability and quality of relevant data. Estimates of adverse birth outcomes estimates from nationally representative surveys are not available in many LMICs, and we therefore used the latest modeled prevalence estimates of LBW, PTB and SGA, which are limited by the quality of the data used in the models. Further, five countries (Dominica, Republic of Kosovo, Marshall Islands, Nauru, and Tuvalu) were excluded from the analysis because they lacked data on the number of live births and probability of survival.”

**8. What are the strengths and limitations of the study? These should be spelt out clearly in a separate paragraph.**

Response: We have restructured the discussion section to include a separate paragraph on the strengths and limitations of our analysis.

Line 513: “Our study has several limitations and strengths. Our estimates are limited by the availability and quality of relevant data. Estimates of adverse birth outcomes estimates from nationally representative surveys are not available in many LMICs, and we therefore used the latest modeled prevalence estimates of LBW, PTB and SGA, which are limited by the quality of the data used in the models. Further, five countries (Dominica, Republic of Kosovo, Marshall Islands, Nauru, and Tuvalu) were excluded from the analysis because they lacked data on the number of live births and probability of survival. Ultrasound dating to assess gestational age is not common in LMICs and therefore estimation of the national prevalences of PTB and SGA at the population-level is particularly challenging in LMICs. However, our study also has several strengths. We used a first order Monte Carlo simulation propagated uncertainty in LBW, PTB and SGA are were therefore able account for variation in parameters and compute bootstrapped confidence intervals. 31 In addition, we quantified the impact of reducing adverse birth outcomes to their theoretical minima on lifetime earnings exclusively through educational attainment. Therefore, our estimates for lifetime earnings are likely conservative since they do not take into account for additional benefits on lifetime earnings that may be accrued through pathways outside of schooling, such as reductions in morbidity, well-being, and the associated increases in adult productivity and lower healthcare costs.11,32,33 In addition, reductions in adverse birth outcomes would reduce child mortality that would lead to more children surviving, enrolling in school, and leading productive lives which are additional benefits on top of the ones accounted for in our model.”

**9. There should be a paragraph/section on the conclusions/recommendations of the study**

Response: We have added the following paragraph on conclusions/ recommendation as the last paragraph of the discussion:

Line 556: “The potential education and lifetime earnings gains from reducing LBW, PTB, and SGA globally may be substantial. The long-term gains documented in this analysis should be considered in cost-effectiveness evaluations of interventions that improve birth outcomes in addition to more immediate benefits on child mortality, growth, and development. As a result, greater investment in interventions and programs that improve birth outcomes may benefit individuals across the life course and provide substantial population-level human capital returns.”

**The paper appears strong, therefore, addressing the reviewers as well as my comments will bring it to a publishable state.**

Response: We thank you for the thoughtful comments provided on our manuscript and analysis. We have revised according to the comments you and the reviewers provided and hope that the edits have been made to your satisfaction.

**Reviewers' comments:**

**Reviewer #1: The manuscript entitled “Impact of birth outcomes on education and wages in adulthood” was organized based on PLOS Global Public Health’s publication criteria. The manuscript is grammatically well organized. The manuscript was technically sound. The study used meta-analysis; it is a good strength of the manuscript and indicated as it is a methodological manuscript. But the author must re-write the methodology part with a detailed explanation of statistical analysis methods. I have no concern about the dual publication of the manuscript. Generally it is suitable for publication.**

Response: Thank you for your thoughtful review of our manuscript and analysis. We have added a working example calculation to clarify to the reader the statistical analysis performed.

**Reviewer #2: The paper was well thought out and written.**

Response: Thank you for your thoughtful comments and the time you have taken to review our manuscript. We have responded to each of your requests below.

**The explanation on fixing the theoretical minimum prevalence needs additional explanation on parameters included for modelling.**

Response: Thank you for drawing our attention to the somewhat brief description of scientific construct behind the theoretical minimum prevalence. We have revised as follows to make this clearer:

Line 146: “Risk assessment requires a reference scenario, a hypothetical counterfactual comparison group. The theoretical minimum-risk exposure distribution (TMRED) is a distribution of exposures corresponding to the lowest levels of risk. In the case of this analysis, the TMRED represents the theoretical prevalence of adverse birth outcomes in a population of healthy pregnancies in low- and middle-income countries. We used evidence from the INTERGROWTH-21st population-based study to define the theoretical minimal prevalence of LBW, SGA and PTB.12 From a cohort of 59,137 women across eight countries, the project selected 20,486 women meeting strict individual eligibility criteria for pregnancies with low risk of impaired fetal growth to construct normative anthropometric standards for newborn size for use across multiethnic populations. In the absence of socio-economic, health, or nutritional risk factors, LBW prevalence was 3.2% and PTB prevalence was 5.5%.”

**The inclusion criteria for the subjects from the cohort should be given clearly.**

Response: Our analysis is conducted on secondary datasets only. For inclusion criteria of the subjects in the cohorts from which we derive our model parameters, the reader is encouraged to read the references from listed in the original meta analyses.

**The negative sign of the estimates in line 128 and line 130 is missing but presented in Table.1.**

Thank you for bringing this to our attention. We have corrected the omission on both lines.

**The mention of 95% C.I is not there in line 130 and line 131.**

Thank you for bringing this to our attention. We have added the mention of the 95% CI on both lines.

**Recommendations:**

**The low and middle income countries have high prevalence of LBW, closer to 20%. Hence it will be helpful for the policy makers if the potential gain was calculated for higher prevalence of 10% also.**

Response: Thank you to the reviewer for this comment -- we have considered adding an intermediate reduction for LBW (ie. 10% LBW prevalence). However, based on a few issues related to the analysis and interpretation, we believe the addition of an intermediate reduction would be challenging to interpret and would likely make the manuscript more difficult to understand for readers. In terms of analytic issues, an intermediation reduction to 10% LBW will be a different set of countries than the current analysis since any country with less than or equal to 10% LBW would be excluded from the analysis; as for example, large countries like China, Indonesia, and any others with less than or equal to 10% prevalence will be excluded. In addition, the reduction to an intermediate point set by our team (which may be considered slightly arbitrary) is difficult to interpret for countries that are close to the threshold- for example, Tanzania has a 10.5% prevalence of LBW, so we would present an estimate of benefits related to a 0.5% reduction in LBW which is not a meaningful target for policy and programs in the country. Therefore, we believe that using the theoretical minimum alone creates a well-defined and scientifically grounded target for all countries, and importantly with this target the results are also easily interpreted as the schooling and economic benefit to eliminating all excess adverse birth outcomes.

**It will be helpful for the readers if the authors show the calculation of school years gain, increase in life time earning and Benefits by cohort lifetime wages for one country with all needed information.**

Response: Thank you for this suggestion. We have added a working example calculation to the appendix (Supplement 2).

**Journal Requirements:**

**1. Please review your reference list to ensure that it is complete and correct. If you have cited papers that have been retracted, please include the rationale for doing so in the manuscript text, or remove these references and replace them with relevant current references. Any changes to the reference list should be mentioned in the rebuttal letter that accompanies your revised manuscript. If you need to cite a retracted article, indicate the article’s retracted status in the References list and also include a citation and full reference for the retraction notice.**

Response: We confirm that the list of references is complete and correct.

**2. In your ethics statement, please provide additional information about the data used in your retrospective study. Specifically, please ensure that you have discussed whether all data were fully anonymized before you accessed them.**

Response: The manuscript presents the results of a population-level epidemiological modeling study, which does not rely on primary data. All data collected were publicly available in de-identified format. We have revised the methods section to make this clear to the reader, and added the following ethics statement:

Line 220: “This analysis uses secondary datasets that are publicly available through the referenced sources.”

**3. We ask that you please remove citations for unavailable and unpublished work, including manuscripts that have been submitted but not yet accepted (e.g., “unpublished work,” “data not shown”). Instead, include those data as supplementary material or deposit the data in a publicly available database.**

Response: We have updated the references to include only published articles.

**4. Please update the completed 'Competing Interests' statement, including any COIs declared by your co-authors. If you have no competing interests to declare, please state "The authors have declared that no competing interests exist". Otherwise please declare all competing interests beginning with the statement "I have read the journal's policy and the authors of this manuscript have the following competing interests:"**

Response: We have updated the Competing Interests statement to state the following: “The authors have declared that no competing interests exist”

**5. Please ensure that all Supporting Information files are included correctly and that each one has a legend listed in the manuscript after the references list.**

Response: We added the legends for the supporting information in the manuscript after the references list.

**6. Please amend your Data Availability Statement and indicate where the data may be found**

Response: We have added the following Data Availability Statement:

“All data used is publicly available and accessible through the sources referenced in the manuscript. The code used in the analysis is available upon reasonable request.”

**7. Please provide us with a direct link to the base layer of the map used in Figure 1 and ensure this location is also included in the figure legend.**

**Please note that, because all PLOS articles are published under a CC BY license (creativecommons.org/licenses/by/4.0/), we cannot publish proprietary maps such as Google Maps,** **Mapquest or other copyrighted maps. If your map was obtained from a copyrighted source please amend the figure so that the base map used is from an openly available source.**

**Please note that only the following CC BY licences are compatible with PLOS licence: CC BY 4.0, CC BY 2.0 and CC BY 3.0, meanwhile such licences as CC BY-ND 3.0 and others are not compatible due to additional restrictions. If you are unsure whether you can use a map or not, please do reach out and we will be able to help you.**

**The following websites are good examples of where you can source open access or public domain maps:**

*** U.S. Geological Survey (USGS) - All maps are in the public domain. (http://www.usgs.gov)**

*** PlaniGlobe - All maps are published under a Creative Commons license so please cite “PlaniGlobe, http://www.planiglobe.com, CC BY 2.0” in the image credit after the caption. (http://www.planiglobe.com/?lang=enl)**

*** Natural Earth - All maps are public domain. (http://www.naturalearthdata.com/about/terms-of-use/)**

Response: The maps were made using the open-access spData package in R. The maps come from maps come from https://www.naturalearthdata.com/.

**8. Please amend your detailed Financial Disclosure statement. This is published with the article, therefore should be completed in full sentences and contain the exact wording you wish to be published.**

**i) Please include all sources of funding (financial or material support) for your study. List the grants (with grant number) or organizations (with url) that supported your study, including funding received from your institution.**

**ii). State the initials, alongside each funding source, of each author to receive each grant.**

**iii). State what role the funders took in the study. If the funders had no role in your study, please state: “The funders had no role in study design, data collection and analysis, decision to publish, or preparation of the manuscript.”**

Response: We have revised the Financial Disclosure statement as follows: “This study and its authors were funded by the Bill and Melinda Gates Foundation (OPP1198520). NP was partially supported by the Canadian Institutes of Health Research Fellowship. The funders had no role in study design, data collection and analysis, decision to publish, or preparation of the manuscript.”
